# Germline-targeting HIV vaccination induces neutralizing antibodies to the CD4 binding site

**DOI:** 10.1126/sciimmunol.adk9550

**Published:** 2024-08-30

**Authors:** Tom G Caniels, Max Medina-Ramìrez, Shiyu Zhang, Sven Kratochvil, Yuejiao Xian, Ja-Hyun Koo, Ronald Derking, Jakob Samsel, Jelle van Schooten, Simone Pecetta, Edward Lamperti, Meng Yuan, María Ríos Carrasco, Iván del Moral Sanchez, Joel D Allen, Joey H Bouhuijs, Anila Yasmeen, Thomas J Ketas, Jonne L Snitselaar, Tom PL Bijl, Isabel Cuella Martin, Jonathan L Torres, Albert Cupo, Lisa Shirreff, Kenneth Rogers, Rosemarie D Mason, Mario Roederer, Kelli M Greene, Hongmei Gao, Catarina Mendes Silva, Isabel JL Baken, Ming Tian, Frederick W Alt, Bali Pulendran, Michael S Seaman, Max Crispin, Marit J van Gils, David C Montefiori, Adrian B McDermott, François J Villinger, Richard A Koup, John P Moore, Per Johan Klasse, Gabriel Ozorowski, Facundo D Batista, Ian A Wilson, Andrew B Ward, Rogier W Sanders

**Affiliations:** 1 Amsterdam UMC, location AMC, University of Amsterdam, Department of Medical Microbiology and Infection Prevention, Amsterdam, the Netherlands.; 2 Amsterdam Institute for Infection and Immunity, Infectious Diseases, Amsterdam, the Netherlands.; 3 Department of Integrative Structural and Computational Biology, The Scripps Research Institute, La Jolla, CA, USA.; 4 The Ragon Institute of Mass General, MIT and Harvard, Cambridge, MA, USA.; 5 Vaccine Research Center (VRC), NIAID, NIH, Bethesda, MD, USA.; 6 Institute for Biomedical Sciences, The George Washington University, Washington DC, USA.; 7 School of Biological Sciences, University of Southampton, Southampton, United Kingdom.; 8 Department of Microbiology and Immunology, Weill Medical College of Cornell University, New York, NY, USA.; 9 New Iberia Research Center, University of Louisiana at Lafayette, New Iberia, LA, USA.; 10 Duke University Medical Center, Durham, NC, USA.; 11 HHMI, Boston Children’s Hospital, Harvard Medical School, Boston, MA, USA.; 12 Institute for Immunity, Transplantation and Infection, Stanford University, Stanford, CA, USA.; 13 Center for Virology and Vaccine Research, Beth Israel Deaconess Medical Center, Boston, MA, USA.; 14 Department of Biology, Massachusetts Institute of Technology, Cambridge, MA, USA.; 15 The Skaggs Institute for Chemical Biology, The Scripps Research Institute, La Jolla, CA, USA.

## Abstract

Eliciting potent and broad neutralizing antibodies (bnAbs) is a major goal in HIV-1 vaccine development. Here, we describe how germline-targeting immunogen BG505 SOSIP germline trimer 1.1 (GT1.1), generated through structure-based design, engages a diverse range of VRC01-class bnAb precursors. A single immunization with GT1.1 expands CD4 binding site (CD4bs)-specific VRC01-class B cells in knock-in mice and drives VRC01-class maturation. In non-human primates (NHPs), GT1.1 primes CD4bs-specific neutralizing serum responses. Selected monoclonal antibodies (mAbs) isolated from GT1.1-immunized NHPs neutralize fully glycosylated BG505 virus. Two mAbs, 12C11 and 21N13, neutralize subsets of diverse heterologous neutralization-resistant viruses. High resolution structures reveal that 21N13 targets the same conserved residues in the CD4bs as VRC01-class and CH235-class bnAbs despite its low sequence similarity (~40%), while mAb 12C11 binds predominantly through its heavy chain complementary determining region 3. These preclinical data underpin the ongoing evaluation of GT1.1 in a phase I clinical trial in healthy volunteers.

## Introduction

With no approved vaccines 35 years after the first HIV vaccine trial started, a preventive HIV vaccine remains a major unmet healthcare need. It is generally believed that a protective vaccine should induce antibodies that can neutralize a wide variety of HIV-1 strains. Such broadly neutralizing antibodies (bnAbs) target the trimeric envelope glycoprotein (Env), the only virally encoded protein present on the surface of HIV-1 virions. Passively transferred bnAbs provide protection against HIV-1 acquisition in non-human primate (NHP) models (1, 2), while the antibody-mediated protection (AMP) trial showed that passive administration of bnAb VRC01 provided protection in humans against VRC01-sensitive isolates (3). Such studies support efforts to create HIV vaccines that induce bnAbs with similar properties.

A proportion of HIV-1 infected individuals develop bnAbs, but generally only after years of infection (4) because this process requires iterative cycles of B cell affinity maturation in response to virus mutation and escape. bnAbs target conserved Env epitopes, including but not limited to the CD4 receptor binding site (CD4bs), the interface between Env subunits gp120 and gp41 and the trimer apex quaternary epitope (5, 6). To overcome the enormous sequence diversity and the shielding of conserved epitopes on the Env trimer by N-linked glycans, bnAbs generally undergo extensive somatic hypermutation (SHM) of up to 40%. Mimicking the arduous process of bnAb induction is a key, but very challenging focus of HIV-1 vaccine development. An additional complication is that only a subset of naive B cells, with specific genetic features, can become bnAb-producing cells. As an important first step, we therefore need carefully designed HIV-1 Env immunogens to prime naive B cells that have the intrinsic genetic capacity to produce bnAbs. Targeting naive B cells with specific genetic signatures is termed “germline-targeting”, and several HIV-1 germline-targeting immunogens are now in phase I clinical trials (NCT05471076, NCT05001373, (7)), including the one described in this study (NCT04224701). As germline-targeting immunogens are extensively modified forms of Env, they are not expected to induce bnAbs when used as stand-alone immunogens. Instead, they are intended to prime desirable precursor B cells that are subsequently exposed to “shaping” and “polishing” immunogens with properties that increasingly resemble wild-type Env. The goal of this multi-step strategy is to mold antibodies so that they eventually recognize and neutralize diverse primary viruses (8, 9).

The CD4bs on HIV-1 Env is a particularly attractive region for germline-targeting strategies. This region is relatively conserved, since binding to CD4 is imperative for infectious virions to fuse with target cells. Furthermore, the genetic signatures of several subsets of CD4bs-targeting bnAbs have been well-defined. bnAbs against the CD4bs can be divided into at least three distinct classes based on their mode of recognition, angle of approach and usage of germline BCR genes. The most potent CD4bs-targeting bnAbs, those of the VRC01-class, use the immunoglobulin heavy chain (HC) variable gene (IGHV) 1–2 encoding a hydrophobic complementarity-determining region 2 (CDRH2) and are further defined by a conserved tryptophan (W100b) in their CDRH3 and a shorter-than-average five amino acid-long CDRL3 in the light chain (LC) to avoid steric impediments raised by Env glycans, most notably the N276 glycan. Named after its first member, VRC01, the VRC01-class of bnAbs mimics the interaction with CD4 and includes among others, 3BNC60, N49P7, CH31, 12A12 and the most potent and broad CD4bs bnAb identified to date, N6 (2, 10–12). A second class of CD4bs bnAbs uses the closely related IGHV1–46 gene segment (11, 13, 14). These CH235-class bnAbs, which include and include CH235, 8ANC131, 1B2530 and 1–18, have a similar orientation to the VRC01-class and mimic the CD4-Env interaction; however, they do not have the typical tryptophan residue W100b in the CDRH3 or the short five amino acid CDRL3 (14). A third class of CD4bs bnAbs includes CH103, VRC13, VRC16 and HJ16 and predominantly uses the CDRH3 to contact the conserved CD4bs (15, 16).

Eliciting these types of CD4bs bnAbs is challenging due to shielding by glycans, and because wild-type Env proteins do not bind to their germline precursors on naive B cells (17–19). Germline-targeting strategies revolve around removal of steric impediments and addition of contact residues on the germline-targeting Env immunogen, with several distinct approaches showing effective priming of VRC01-class B cells in preclinical animal models and, most recently, in human trials (7, 9, 19–23). One such strategy involves native-like Env trimers, including ones using the SOSIP technology (39), that present epitopes in a similar manner as displayed on infectious virions. In this way, structural constraints are imposed that limit the number of permissible approach angles and that select for B cells able to recognize Env in a natural context. The closely related germline-targeting SOSIP trimers BG505 GT1 and GT1.2 were specifically engineered to engage precursors of both the VRC01-class and the V1/V2 apex bnAb classes (9, 19). While the prototypic BG505 GT1 activated VRC01-class B cells and bound to some inferred VRC01-class precursors in initial studies, its recognition range of VRC01-class precursors was limited and warranted further optimization (19).

## Results

### GT1.1 engages a broad range of VRC01-class bnAb precursors

**Figure 1.**
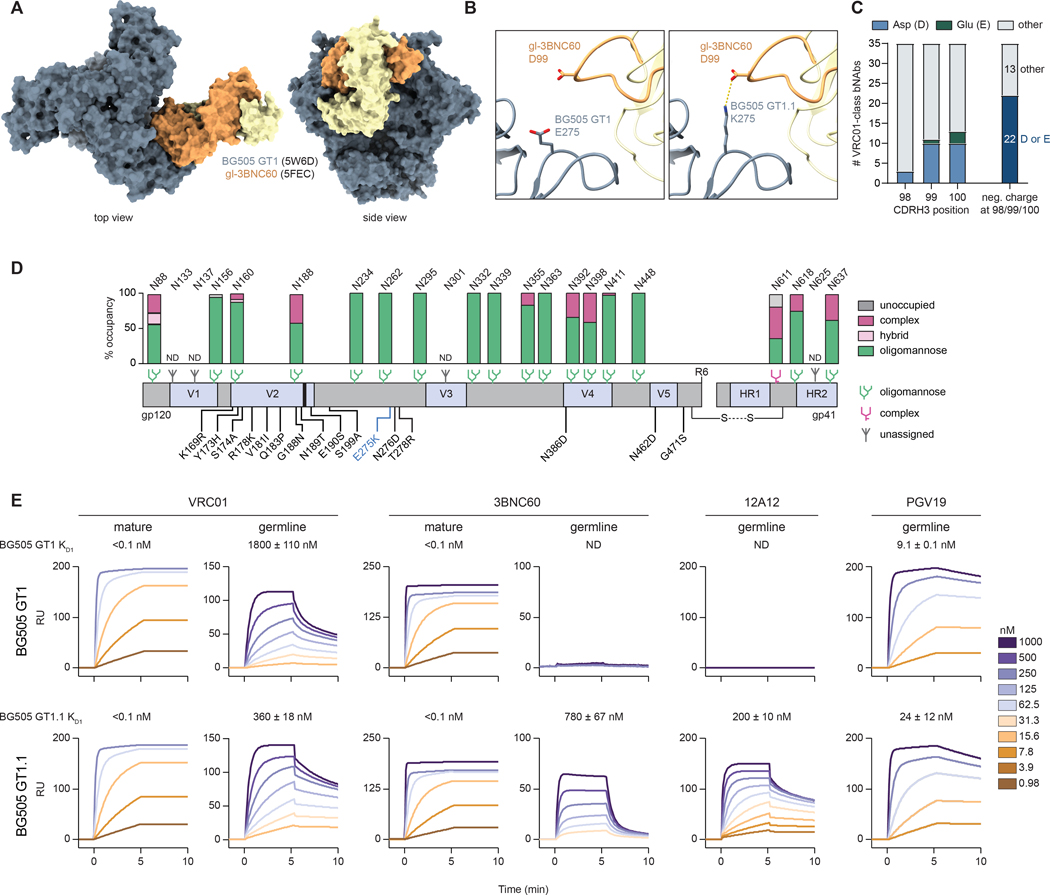
Design and antigenicity of BG505 SOSIP germline trimer 1.1 (GT1.1). (A) Structural overlay of BG505 SOSIPv4.1-GT1 (PDB: 5W6D) and gl-3BNC60 (PDB: 5FEC). (B) Detail of the predicted interaction between GT1 E275 (left) and GT1.1 K275 and the CDRH3 residue D99 of gl-3BNC60. (C) Conservation of the negative CDRH3 charges in VRC01-class bnAbs. (D) Schematic linear representation of GT1.1 with glycan occupancy data. All amino acid mutations compared to BG505 SOSIP are indicated in black, whereas the blue E275K mutation defines GT1.1. The glycan icons represent the predominant type of glycan observed at that N-linked glycosylation site. (E) Surface plasmon resonance (SPR) of antibody binding to GT1 (top panels) and GT1.1 (bottom panels). The sensorgrams show the specific binding signal in response units (RUs) as a function of time. The x axes are truncated at half the dissociation time monitored (Fig. S1C). The *K*_*D1*_ values (nM) fitted by bivalent modeling (Table S1) are indicated on top of the panels. ND, not determined.

The availability of the BG505 GT1 crystal structure enabled precise assessment of VRC01-class precursor contact residues in the context of the full Env trimer (19). We identified a glutamic acid (E) at position 275 that is expected to be disfavored for interaction with negatively charged residues present at positions 98, 99 or 100 (Kabat numbering) in the CDRH3 of 22 VRC01-class bnAbs, including VRC01, 3BNC60 and N6 (Fig. 1A–C). *In silico* substitution of E275 with the positively charged amino acid lysine (K) revealed the potential for an additional interaction between GT1 and VRC01-class precursors (Fig. 1B). Accordingly, we generated the E275K substitution and the resulting trimer, BG505 SOSIP.v4.1 GT1.1, referred to as GT1.1, was expressed successfully in mammalian cells. Moreover, GT1.1 had biochemical and biophysical properties similar to that of other native-like trimers (Fig. S1A) (24). It also exhibited an overall similar glycan profile to that of GT1 and GT1.2 (Fig. 1D) (9, 19), which only differ by one and two residues, respectively, with subtly enhanced glycan processing due to removal of apical glycans compared to the original BG505 SOSIP.664 trimer (termed BG505 SOSIP from hereon), resulting in more complex glycans at the trimer apex (e.g., N188; Fig. 1D and S1B). High resolution cryo-EM structures corroborated that GT1.1 forms native-like trimers with a similar fold as BG505 SOSIP trimers (see below).

Whereas binding of GT1 to the inferred germline precursors gl-3BNC60 and gl-12A12 to GT1 was insufficient to derive a *K*_D1_ value, they bound to GT1.1 with substantial affinity (*K*_D1_ = 780 nM for gl-3BNC60 and 200 nM for gl-12A12) (Fig. 1E, Fig. S1C, Table S1). The E275K substitution also increased the affinity of gl-VRC01 by five-fold (*K*_D1_ value of 360 nM for GT1.1 *versus* 1800 nM for GT1)(Fig. 1E), mainly because of a reduction in the off-rate constant, *k*_off1_ (Table S1). The affinity of gl-PGV19, already high for GT1, was however slightly reduced (*K*_D1_ value of 24 nM for GT1.1 *versus* 9.1 nM for GT1). Moreover, this increase in affinity translated in the ability of GT1.1 to activate gl-VRC01-expressing Ramos B cells more rapidly and robustly compared to GT1 (Fig. S1D). Thus, we validated the E275K substitution present in the GT1.1 trimer as a strategy to increase the range of VRC01-class precursors.

### A single GT1.1 immunization activates diverse VRC01-class precursors *in vivo*

**Figure 2.**
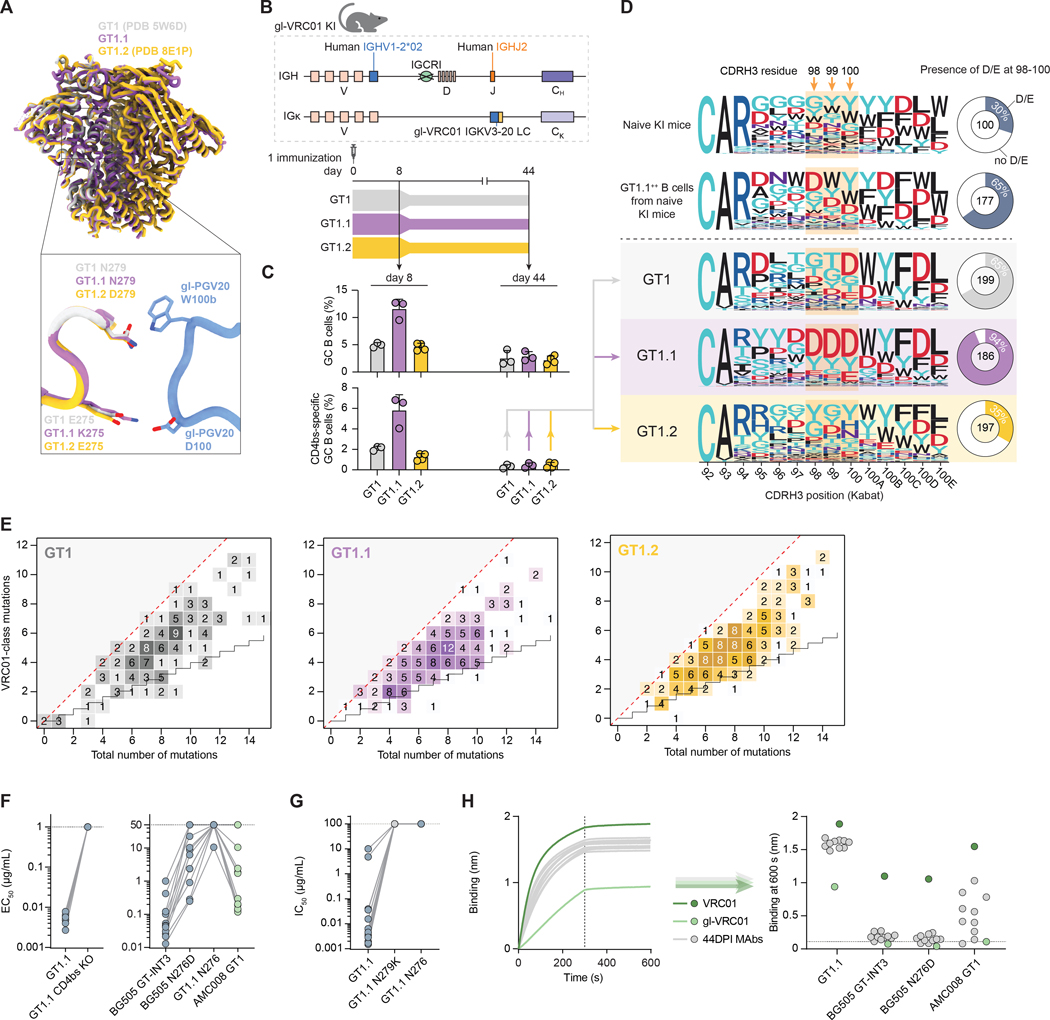
GT1.1 primes diverse VRC01-class precursors in a knock-in mouse model. (A) Schematic showing residues of loop D of GT1, GT1.1 and GT1.2 interacting with gl-PGV20. Note that in contrast to N279D, N279A and N279K reduce VRC01-class binding and are used in knock-out reagents. (B) Schematic of the VRC01-class precursor mouse model (top panel) and the immunization schedule (bottom panel) (n = 3–4 per group). (C) Germinal center (GC) B cell frequency (top panel) and epitope-specific (CD4bs) GC B cell frequency (bottom panel) at day 8 and day 44 post-immunization. (D) Logo plot showing the CDRH3 sequence of knock-in BCRs isolated from each of the groups indicated. The donut plots on the right summarize the percentage of isolated knock-in BCRs with a negative charge at CDRH3 positions 98–100. (E) Total and VRC01-class amino acid mutations in the IGHV1–2 region for recovered knock-in BCR sequences for each immunization group. The staggered black line shows the expected level of VRC01-class mutations as expected to be introduced by random SHM in IGHV1–2 (21). On track VRC01-class mutations are defined as mutations that are shared with VRC01, PGV04, PGV20, VRC-CH31, 3BNC60 or 12A12 (21, 53). (F) Half-maximal effective concentrations (EC_50_s) as a measure for binding for mAbs recovered at 44 days post-immunization (DPI) against the indicated Envs. (G) Half-maximal inhibitory concentrations (IC_50_s) of the mAbs in (D) against the indicated pseudoviruses. (H) Bio-layer interferometry (BLI) sensorgrams (left panel) of isolated mAbs (grey) compared with gl-VRC01 (light green) and VRC01 (dark green) binding to GT1.1 and the binding at 600 s for various other Envs (right panel). Binding was assessed for 300 s, after which the sensors were moved to empty wells to allow dissociation measurement (dotted line). ELISA and neutralization data are representative of at least two independent experiments.

To determine whether GT1.1 could prime VRC01-class precursors *in vivo*, we modified a well-characterized knock-in (KI) mouse model to enable expression of a heavy chain consisting of IGHV1–2*02, mouse D regions, human IGHJ2, and a full gl-VRC01 LC (IGKV3–20*01 with a 5 aa QQYEF CDRL3) (Fig. 2B, Fig. S2A) (25). This model allows for CDRH3 variability through recombination with mouse D regions, allowing *in vivo* assessment whether the E275K substitution allows the recruitment of diverse VRC01-class precursors. Mice were immunized once with soluble trimeric GT1, GT1.1 or GT1.2, each formulated with polyI:C adjuvant (9, 19). GT1, GT1.1 and GT1.2 only differ at positions 275 and 279 in gp120: compared to GT1, GT1.1 has an E275K substitution and GT1.2 has an N279D substitution (Fig. 2A). At day 8 and day 44 post-immunization (Fig. 2B), mice were sacrificed and GT1, GT1.1 or GT1.2-specific splenic B cells were single-cell sorted (Fig. S2B). Antigen-specific germinal center (GC) B cells were detected at both time points (Fig. S2B), but GT1.1 induced a higher frequency of GC B cells compared to GT1 and GT1.2 at day 8 (Fig. 2C), which also resulted in a higher frequency of CD4bs-specific B cells at day 8. At day 44, no differences were observed in GC B cell or epitope-specific GC B cell frequencies (Fig. 2C).

Next, B cell receptors (BCRs) of single-cell sorted epitope-specific splenic B cells were sequenced to assess selection for negatively charged CDRH3 residues as well as (VRC01-class) maturation. Whereas naive KI mice do not generally exhibit any preference for specific amino acids at positions 98, 99 or 100 in the CDRH3, the majority (65%) of GT1.1-specific B cells sorted from these naive mice bore a negatively charged residue at one of these CDRH3 positions (Fig. 2D). After immunization with GT1.1, but not GT1 or GT1.2, such negative charges were enriched, with 94% of splenic GT1.1-specific B cells at day 44 having the intended negative charge in the CDRH3 (Fig. 2D) compared to 30% of total B cells in the naive repertoire and 65% and 35% for GT1 and GT1.2, respectively (Fig. 2D). The CDRH3 length of GT1.1-specific B cells centered around 15 amino acids, similar to VRC01-class bnAbs, whereas GT1.2 appeared to recruit B cells with more diverse CDRH3 lengths (Fig. S2C). Thus, GT1.1 immunization activates diverse VRC01-class precursors and selects for a negative charge in the CDRH3 that is shared among many VRC01-class bnAbs, consistent with the rationale of the E275K mutation.

### A single GT1.1 immunization drives VRC01-class maturation *in vivo*

Next, we evaluated whether SHM was “on-track” and similar to IGHV SHM observed in a representative and diverse set of VRC01-class bnAbs, including VRC01, CH31, PGV04, PGV20, 3BNC60 and 12A12 (21). In all three groups, SHM was high, with up to fifteen amino acid substitutions, and on-track, as indicated by the high numbers of VRC01-class mutations (Fig. 2E). The number of VRC01-class mutations found in these mice, approached the number minimally needed for broad and potent neutralization, which is 11 in the case of minimally mutated (min)VRC01 (26). We also validated GT1.1 in a nanoparticle formulation (Fig. S3). Mice were immunized once with soluble trimeric GT1.1 or two-component I53–50 nanoparticles displaying twenty GT1.1 trimers, each formulated with polyI:C adjuvant (Fig. S3A) (9, 27, 28). At day 8, 16 or 44 post-immunization (Fig. S3A), mice were sacrificed and GT1.1-specific splenic B cells were single-cell sorted (Fig. S3B). GT1.1-specific (germinal center) B cells were detected at all time points (Fig. S3C). On-track SHM and enrichment of GC B cells with a negatively charged CDRH3 was visible as early as day 8 (Fig. S3D–F). Activation of VRC01-class B cells was also observed when GT1.1 was formulated with other adjuvants including alum and Adjuplex (Fig. S3G–K).

To study the functional characteristics of GT1.1-elicted B cells, we expressed ten paired HC/LC sequences from day 44 as IgG1 mAbs and tested their binding characteristics. All 10 mAbs bound strongly to GT1.1, but not to GT1.1 CD4bs KO in which the CD4bs was mutated to prevent binding to VRC01-class Abs by means of the N279A and D368R substitutions, confirming specificity for the CD4bs (Fig. 2F). A GT1.1-based pseudovirus that includes the germline-targeting mutations from GT1.1, but not the stabilizing (SOSIP) mutations (Table S2), was neutralized efficiently by these mAbs, while the CD4bs mutant GT1.1 N279K was not, confirming that these mAbs target the CD4bs (Fig. 2G, Fig. S4A, Table S2). Next, we assessed binding to antigens with increasing resemblance to the fully glycosylated native Env spike, corresponding with candidate shaping and polishing immunogens that could be used in sequential vaccination regimens. All mAbs recognized candidate shaping immunogens BG505 SOSIP INT3 (BG505 SOSIP N276D T278R). 8/10 mAbs also bound to BG505 SOSIP N276D, which is identical to BG505 SOSIP with the exception of the absence of the N276 glycan, indicating an ability to accommodate the majority of glycans surrounding the CD4bs (Fig. 2F). One mAb bound to GT1.1 with the N276 glycan (GT1.1 N276, Fig. 2F, Fig. S4B–D) but none of the mAbs neutralize the GT1.1 N276 pseudovirus (Fig. 2G, Fig. S4A), implying that N276 remains the major bottleneck for Env recognition by these VRC01-class mAbs. All mAbs had a greater affinity for GT1.1 (Fig. 2H) than gl-VRC01, but lower than mature VRC01, properties that might be expected from VRC01-class antibodies early in the maturation pathway towards bnAbs. All mAbs also had greater affinity to candidate shaping and polishing immunogens BG505 SOSIP INT3, BG505 SOSIP N276D and AMC008 GT1 than gl-VRC01 (Fig. 2H). None of the mAbs bound appreciably to fully glycosylated BG505 SOSIP, nor to heterologous SOSIP trimers with or without the N276 glycan. We conclude that GT1.1 priming in gl-VRC01 KI mice activates a diverse set of VRC01-class B cells and drives initial stages of VRC01-class maturation. However, follow-up immunizations with (increasingly) glycosylated trimers are likely needed to facilitate the accommodation of the N276 glycan and acquisition of neutralization breadth.

### GT1.1 priming and SOSIP boosting induces specific antibody responses in NHPs

We next evaluated GT1.1 as a priming immunogen in a non-human primate (NHP) model. NHPs may represent the most appropriate model organism to study HIV-1 Env vaccination regimens because of their close resemblance to humans (29). However, NHPs do not possess an ortholog of human IGHV1–2 and thus are intrinsically unable to generate VRC01-class nAbs, based on the classical genetic signature. Nonetheless, we hypothesized that despite being tailored for VRC01-class B cell activation, a native-like trimer immunogen such as GT1.1 might prime non-VRC01-class CD4bs antibody lineages.

**Figure 3.**
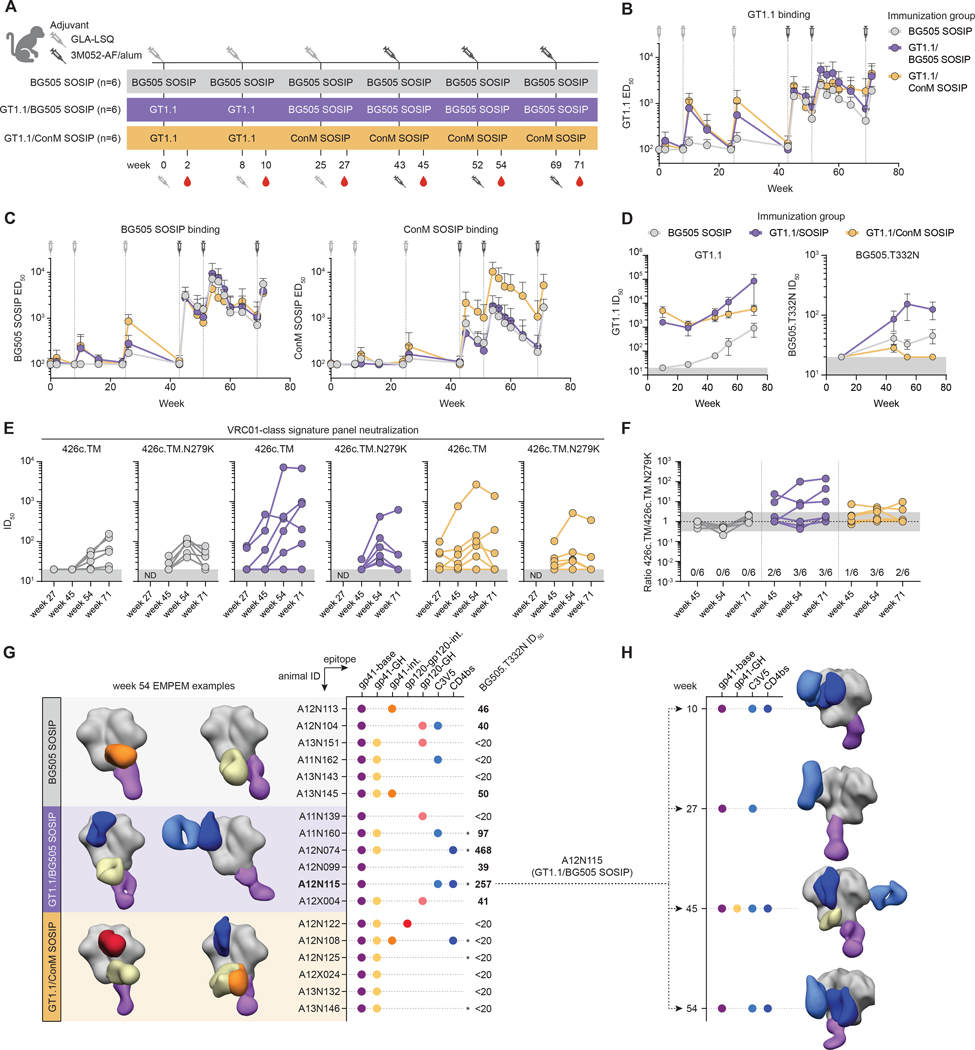
GT1.1 primes CD4bs antibodies in a non-human primate model. (A) Schematic of the immunization regimen (n = 6 animals per group). (B) Mean ± SD half-maximal effective dose titers (ED_50_s) as a measure for binding to GT1.1 of each immunization group. The syringes indicate immunization time points. (C) Mean ± SD ED_50_s to BG505 SOSIP (left panel) and ConM (right panel). (D) Mean ± SD half-maximal inhibitory dose titers (ID_50_s) as a measure for neutralization of each immunization group to GT1.1 (left panel) and BG505.T332N (right panel). The grey boxes represent the minimum titer tested. (E) Mean ID_50_s of each animal to VRC01-class signature viruses 426c.TM and its CD4bs knockout mutant, 426c.TM.N279K. The grey boxes represent the minimum titer tested. ND, not determined. (F) The ratio of ID_50_ titers to 426c.TM and 426c.TM.N279K as in (E). The limits of the grey boxes represent a three-fold increase/decrease. (G) Electron microscopy-based polyclonal epitope mapping (EMPEM) for each immunization group. The polyclonal specificities for two representative animals per group are shown as segmented EM maps. The graph on the right shows positive detection of antibodies to a given epitope by each animal, with the numbers representing the ID_50_ titers to BG505.T332N. (H) Longitudinal EMPEM analysis for animal A12N115. ELISA and neutralization data shown are representative of at least two independent experiments.

We immunized two groups of NHPs (n=6 each) with GT1.1 at weeks 0 and 8, and included a control group that received the archetypal, fully glycosylated BG505 SOSIP (n=6; Fig. 3A). As an attempt to drive the maturation of CD4bs lineages that can accommodate the N276 glycan, the GT1.1 primed animals were boosted at weeks 25, 43, 52 and 69 with either BG505 SOSIP or a consensus-sequence based trimer, ConM SOSIP.v7 (termed ConM SOSIP; Fig. 3A) (31). Both BG505 SOSIP and ConM SOSIP.v7 are being evaluated in clinical trials (NCT03699241, NCT04177355, NCT03816137, NCT03961438), thus providing a translational path forward. The BG505 SOSIP primed control group received additional BG505 SOSIP vaccinations. The adjuvant used for the first three immunizations was GLA-LSQ (32), but the following three immunizations were performed with the Toll-like receptor 7/8 (TLR7/8) agonist 3M-052-AF/alum (Fig. 3A), which proved superior in the context of HIV-1 Env immunization to GLA-LSQ or similar adjuvants (28, 33–35).

Serum binding antibody titers were assessed by ELISA and demonstrated that GT1.1 priming elicits higher binding titers against GT1.1 and weaker responses against BG505 SOSIP and ConM SOSIP trimers at week 10 (Fig. 3B–C). However, serum Ab titers were short-lived and had disappeared by week 25 (Fig. 3B–C). The first boost with either BG505 SOSIP or ConM SOSIP at week 25 elevated serum Abs to titers observed after the first two immunizations, but the response was again short-lived and became undetectable at week 43. After the fourth immunization at week 43, the first immunization using 3M-052-AF/alum as the adjuvant, serum Ab titers were substantially higher and remained high throughout the remainder of the immunization schedule, consistent with the superiority of 3M-052-AF/alum over GLA-LSQ as the adjuvant (Fig. 3B–C). Binding antibody titers against GT1.1 and BG505 SOSIP were comparable between all groups, whereas the ConM SOSIP-boosted animals generally exhibited higher binding against the ConM trimer (Fig. 3B–C).

The neutralizing capacity of the sera largely followed the binding phenotypes. After two immunizations, all GT1.1-recipient animals neutralized the BG505 GT1.1 virus (Fig. 3D, Fig. S5A). In contrast, none of the BG505 SOSIP-primed animals neutralized the GT1.1 virus at week 10 and remained inferior to GT1.1-recipient animals after the booster vaccinations compared to GT1.1-recipient animals (Fig. 3D). BG505 SOSIP boosting further increased GT1.1 neutralization in GT1.1-primed animals, while ConM SOSIP boosting maintained GT1.1 neutralization but did not enhance it (Fig. 3D). BG505.T332N neutralization, albeit weakly, was detected in all groups at week 45, with the GT1.1 prime/BG505 SOSIP-boosted group exhibiting the highest neutralizing titers (Fig. 3D). The ConM SOSIP-boosted animals lost all neutralizing activity against BG505.T332N after their second ConM SOSIP immunization but gained the capacity to neutralize the ConM pseudovirus (Fig. S5B).

### GT1.1 priming and SOSIP boosting elicits CD4bs-directed neutralizing antibodies in NHPs

We next utilized an established VRC01-class signature virus panel (36). The 426c.TM virus lacks glycans situated around the CD4bs (N276/N460/N463) and is more sensitive to CD4bs-directed nAbs, whereas the mutant 426c.TM.N279K virus is insensitive to most CD4bs-directed nAbs, thus presenting a tool to measure CD4bs-directed neutralization. BG505 SOSIP-primed animals did not exhibit high nAb titers against either the signature virus or its epitope knockout, suggestive of the absence of CD4bs-directed nAbs (Fig. 3E). In contrast, animals primed with GT1.1 generally had high titers against 426c.TM and lower titers against 426c.TM.N279K, which was most apparent in animals boosted with BG505 SOSIP (Fig. 3E). We calculated the ratio of neutralization between 426c.TM and its epitope knockout 426c.TM.N279K, where we consider that a ratio >3 (i.e., >3 higher neutralization against 426c.TM than its epitope knockout variant) is strongly indicative for the presence of CD4bs-directed specificities (Fig. 3F). Throughout the study, none of the BG505 SOSIP-primed animals exhibit neutralization mediated by CD4bs nAbs, while this was the case for 50% of the GT1.1-primed animals (Fig. 3F). Notably, the GT1.1 prime/BG505 SOSIP boost group had the highest ratio of 426c.TM/426c.TM.N279K, with one animal having a decrease of >100-fold in neutralization to the 426c.TM.N279K knockout (Fig. 3F). A similar picture was observed for the GT1.1 prime/ConM SOSIP boost group, although the response rate and magnitude of the nAb response were lower (Fig. 3F). We note that the clade C 426c indicator virus is a heterologous virus in the context of this study and might not capture all CD4bs specificities, thereby potentially underestimating the presence of CD4bs specificities. Taken together, these results provide evidence that GT1.1 priming followed by BG505 SOSIP or ConM SOSIP is conducive to the induction of CD4bs-directed antibodies.

We performed electron microscopy-based polyclonal epitope mapping (EMPEM) with GT1.1 to identify the dominant serum antibody responses (Fig. 3G–H, Fig. S5C–D) (37, 38). All animals across the three immunization groups developed antibody responses against the trimer base and 12/16 animals had antibody specificities against the gp41 glycan hole around N611 (Fig. 3G). Both are known off-target responses induced by SOSIP trimers (39, 40). One animal in the GT1.1/ConM SOSIP group developed a detectable response to the gp120-gp120 interface, which involves the V1V2 region. This finding is consistent with previous studies showing the the V1V2 region on ConM is immunodominant on the ConM SOSIP trimer (28, 31). A subset of GT1.1-primed animals showed polyclonal CD4bs specificities among the dominant responses, whereas none of the animals in the BG505 SOSIP group did (Fig. 3G), which is consistent with previous studies demonstrating an absence of any dominant serum CD4bs specificities after BG505 SOSIP immunization (37, 38, 41–44). Strikingly, the CD4bs specificity in animal A12N115 was detectable as soon as week 10, indicating that it was primed by GT1.1 immunization and maintained by SOSIP boosting (Fig. 3H). Moreover, the CD4bs specificity observed in animal A12N074 at week 54 was also visible when the serum was complexed with BG505 SOSIP, demonstrating accommodation of the N276 glycan by CD4bs-specific serum antibodies (Fig. S5D). Two animals had polyclonal specificities to the gp120-gp41-interface (Fig. 3G, orange dots), which may include fusion peptide epitope antibodies and nearby glycan-hole antibodies.

### Monoclonal antibodies reveal the CD4bs and the fusion peptide as targets

**Figure 4.**
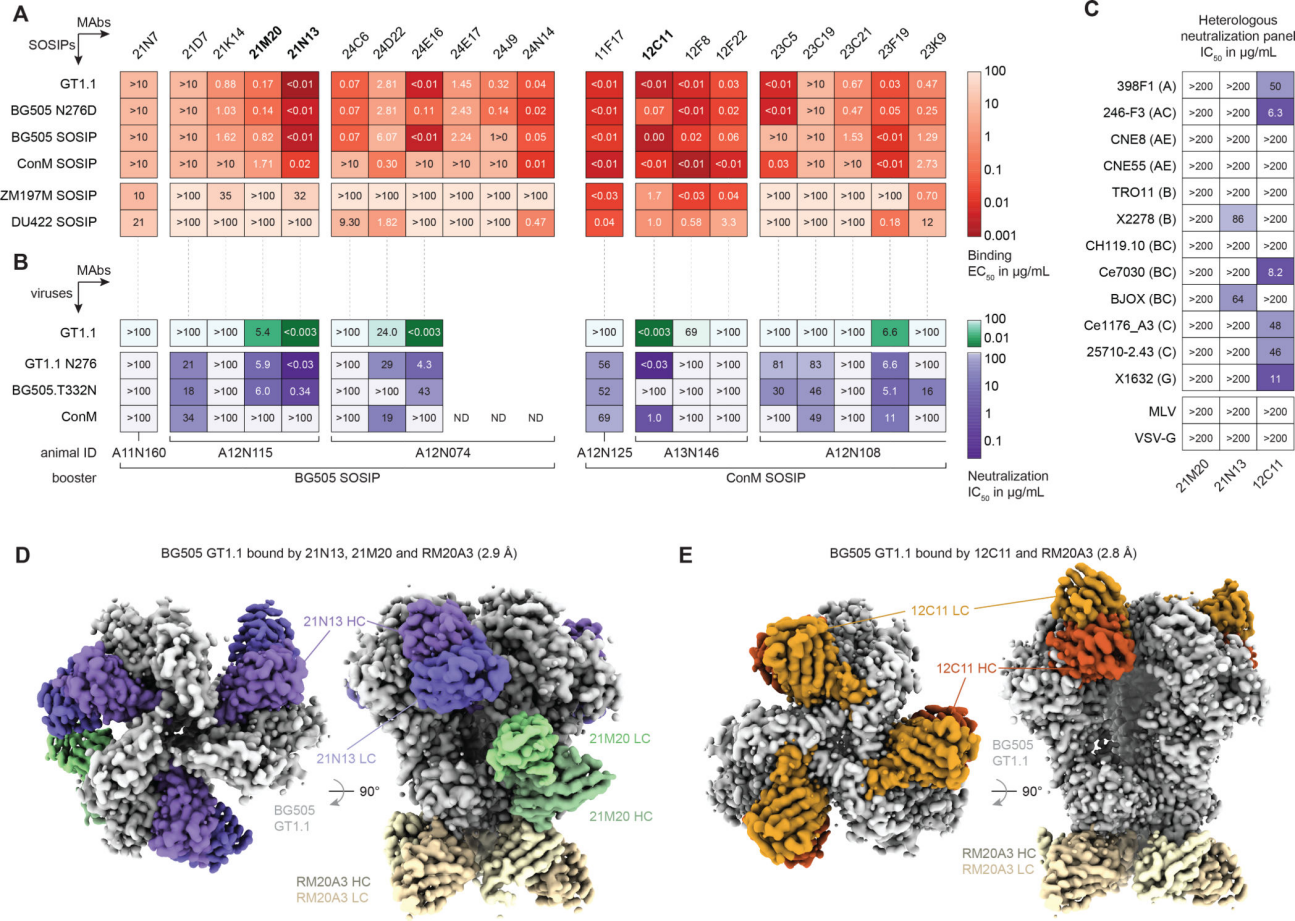
Monoclonal antibodies neutralize fully glycosylated viruses through targeting of conserved (CD4bs) epitopes. (A-B) Overview of the isolated mAbs (label on top) in binding (EC_50_, (A)) and neutralization (IC_50_ (B)). The animal IDs and booster immunogens these animals received are indicated in (B). The dotted lines connecting (A) and (B) depict binding and neutralization of a particular mAb. (A) EC_50_ titers of the isolated mAbs (label on top) to each indicated SOSIP protein (labels on left). (B) IC_50_ titers of the isolated mAbs as in (A) to each indicated pseudovirus. ND, not determined. The first three animals belong to the GT1.1/BG505 SOSIP group, the second three animals to the GT1.1/ConM SOSIP group. (C) Table showing the IC_50_ titers of the isolated mAbs (label on bottom) against the indicated heterologous pseudoviruses. The letter in parentheses represents the HIV-1 clade of a particular pseudovirus. (D) Top (left) and side (right) view of the 2.9 Å cryo-EM reconstruction of the GT1.1–21N13–21M20-RM20A3 complex. (E) Top (left) and side (right) view of the 2.8 Å cryo-EM reconstruction of the GT1.1–12C11-RM20A3 complex. HC, heavy chain; LC, light chain. Data shown in A-C are representative of at least two independent experiments.

Next, we isolated and characterized mAbs from the animals that displayed CD4bs-mediated VRC01-class signature neutralization (Fig. 3F). Briefly, GT1.1-specific B cells were cultured and selected for sequencing based on a combination of *ex vivo* probe binding, B cell growth and microneutralization of 426c.TM (Fig. S6A–B). In total, 20 unique paired HC/LC sequences were obtained and cloned into a human IgG1 vector for characterization by enzyme-linked immunosorbent assay (ELISA) and pseudovirus neutralization assay (Fig. 4A, Fig. S6C, Table S3). Most isolated mAbs exhibited 5–10% of SHM in their HCs, suggesting sustained presence in GCs or re-entry upon boosting with BG505 SOSIP (Fig. S6C). 17/20 (85%) of expressed mAbs showed binding to GT1.1 and BG505 SOSIP N276D; 15/17 (88%) and 11/17 (65%) of those binding to GT1.1 showed binding to BG505 SOSIP and ConM SOSIP, respectively (Fig. 4A). Moreover, mAbs from animal A13N146 showed strong cross-binding to SOSIP trimers of clade C isolates ZM197M and DU422 (Fig. 4A).

A subset of these mAbs was evaluated in neutralization assays. First, we tested the mAbs against an autologous virus panel consisting of GT1.1, GT1.1 N276 and fully glycosylated BG505.T332N and ConM (Fig. 4B). 6/20 mAbs (30%) neutralized GT1.1 at IC_50_ values lower than 100 μg/mL and three did so ultrapotently (IC_50 of_ <3 ng/mL). 10/20 mAbs (50%) neutralized GT1.1 N276, while 9/20 (45%) mAbs neutralized fully glycosylated BG505.T332N and 6/20 (30%) neutralized the ConM virus. Three mAbs were selected for structural analyses (see below). Of these, mAb 21N13, derived from the BG505 SOSIP-boosted animal that demonstrated serum CD4bs specificity as early as week 10 (Fig. 3H), neutralized GT1.1 pseudovirus ultrapotently (IC_50 of_ <3 ng/mL), while also neutralizing GT1.1 N276 (IC_50_ of <0.03 μg/mL), fully glycosylated BG505.T332N (IC_50_ of 0.34 μg/mL), and heterologous viruses BJOX and X2278, albeit weakly (IC_50_s of 64 and 86 μg/mL, respectively; Fig. 4B–C). 12C11, derived from a ConM SOSIP-boosted animal, neutralized GT1.1 ultrapotently (IC_50 of_ <3 ng/mL), also neutralized GT1.1 N276 and ConM (IC_50_s of <0.03 and 1.0 μg/mL, respectively), but not BG505.T332N (Fig. 4B). Furthermore, 12C11 neutralized 6/12 viruses (50%) of a panel of heterologous tier 2 viruses (IC_50_s ranging from 6.3 to 50 μg/mL; Fig. 4C), as well as 6/12 (50%) of the AMP placebo panel that includes contemporaneous circulating viral strains (Fig. S6D). We also tested an extended multiclade panel that represents global HIV-1 diversity (Table S4) (45), of which 15/119 are neutralized by 12C11, bringing its total coverage to 19% (27/143). mAb 21M20 neutralized all tested BG505-derived viruses at similar potencies, suggesting that it might not target the CD4bs (Fig. 4B–C), and did not neutralize any heterologous tier 2 viruses.

**Figure 5.**
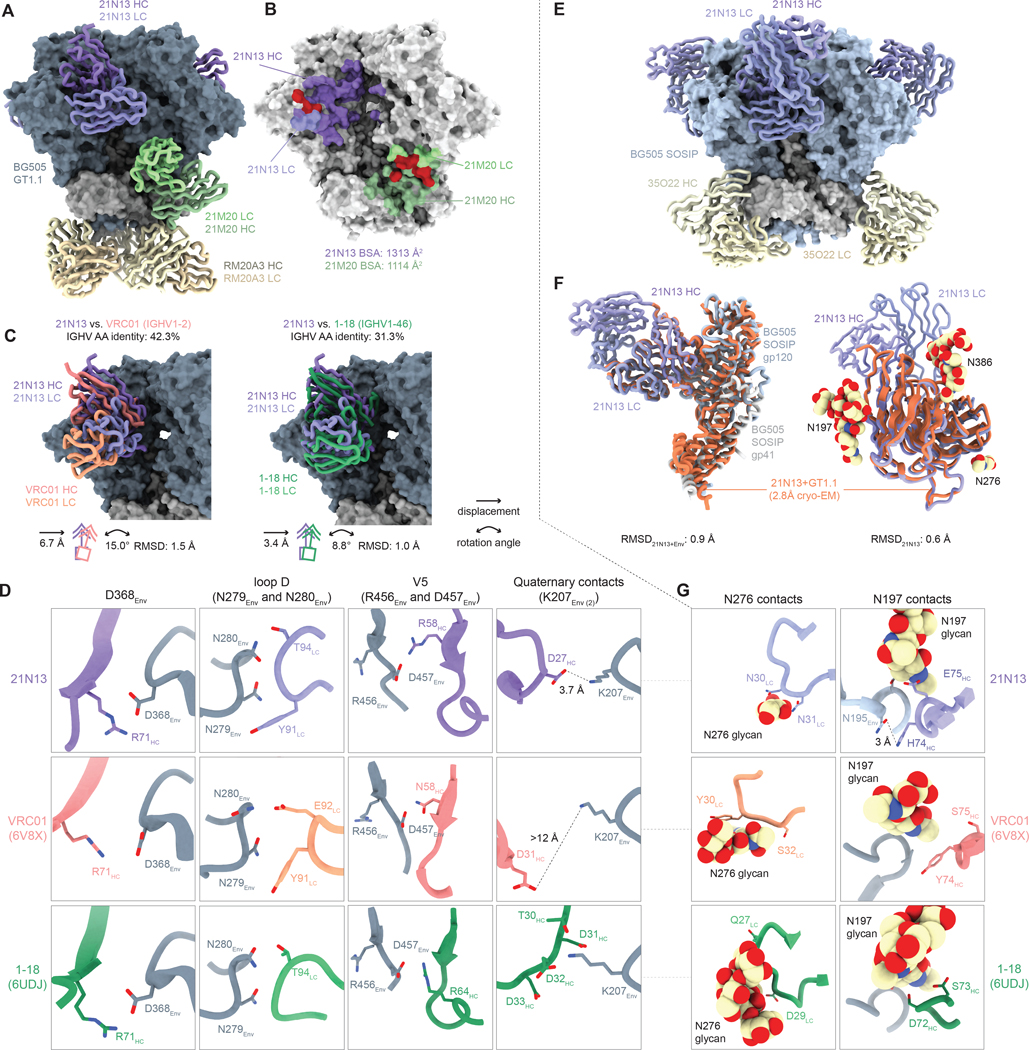
nAb 21N13 contacts a conserved CD4bs epitope and accommodates CD4bs-adjacent glycans. (A) Cryo-EM-derived atomic model of GT1.1 in complex with 21N13, 21M20 and RM20A3 Fabs. (B) Footprint and buried surface area (BSA) of 21N13 and 21M20 Fabs. The red shading indicates Env regions contacted by both heavy chain and light chain. (C) Superimposition of the GT1.1–21N13 structure with published structures of Env-VRC01 (PDB: 6V8X, left panel) and Env-1–18 (PDB: 6UDJ, right panel). The icons below the figure represent the mean displacement and rotation angle as well as the root mean square deviation (RMSD) of VRC01 or 1–18 compared to 21N13. (D) Detailed interactions of 21N13 with GT1.1 (top row) compared to VRC01 (middle row) and 1–18 (bottom row). The structures were aligned on Env fragments and the viewpoints are identical between structures. Dashed lines indicate listed distances. R58_HC_ of 21N13 uses its side chain to form a salt bridge to the conserved D457_Env_. Furthermore, several CD4bs bnAbs such as 1–18, VRC03 and 3BNC117 evolved insertions in their CDRH1 or FWRH3 regions to contact adjacent Env protomers (11, 13, 50). While 21N13 does not bear an insertion, its negatively charged CDRH1 is positioned similarly to that of 1–18, thereby facilitating a contact with K207_Env_ on the adjacent protomer (E) 4.7 Å crystal structure of 21N13 Fabs in complex with BG505 SOSIP and 35O22 Fab. (F) Comparison of the 21N13-GT1.1 cryo-EM structure (orange) with the 21N13-BG505 SOSIP crystal structure (blue/purple), as a whole Env+21N13 comparison (left panel) as well as a 21N13 Fab only comparison, both based on gp120 alignment (right panel). (G) Detailed interactions of 21N13 with BG505 SOSIP around N197 and N276 glycans as in (D).

We selected 21M20, 21N13 and 12C11 for structural characterization. Negative-stain electron microscopy (NS-EM) experiments confirmed that nAbs 21N13 and 12C11 targeted the CD4bs, and revealed that 21M20 bound at the gp120/gp41 interface near the fusion peptide (Fig. S6E) We determined high resolution cryo-EM structures of GT1.1 in complex with nAbs 21M20 and 21N13, as well as GT1.1 in complex with nAb 12C11 (Fig. 4D–E, Fig. S7D–G, Table S5). Both structures also included base-binding mAb RM20A3 to help with particle orientation (46, 47). We also solved crystal structures of fully glycosylated BG505 SOSIP with 21N13 (Fig. 5E, Table S6), and of unliganded 21N13 Fab (Fig. S7A–C, Table S6).

### CD4bs-specific nAb 21N13 shares contacts with human bnAbs VRC01 and 1–18

NAb 21N13 binds the GT1.1 trimer with a 3:1 Ab:Env stoichiometry, similar to known human bnAbs targeting the CD4bs (Fig. 5A) (48). The 21N13 HC contributes most of the buried surface area (BSA) (1138 Å^2^ *versus* 218 Å^2^ by the LC; Fig. 5B). Similar to many CD4bs bnAbs, such as 3BNC117, CH31 and 1–18 (11, 13), 21N13 extends towards a neighboring protomer in the Env trimer, which contributes 73 Å^2^ to its total BSA (Fig. 5B), suggesting that immunization with a native-like Env trimer might have selected for these contacts. 21N13 has low amino acid sequence identity to known CD4bs bnAb classes, including VRC01 (42%), 1–18 (31%) and IGHV4–59-derived CH103 (68%) (16) (Fig. S8A). Compared to VRC01, 21N13 contacted the Env trimer with a rotation angle of 15° and a displacement of 6.7 Å (Fig. 5C). While genetically most distant from 1–18, 21N13 targets a highly similar epitope with a similar approach angle (Fig. 5C).

Despite this low sequence identity, 21N13 shares targets identical residues in the CD4bs epitope as VRC01 and/or 1–18, implying that GT1.1 immunization in NHPs and HIV-1 infection in humans led to convergent evolution to target the CD4bs. A defining feature of VRC01-class and CH235-class bnAbs is a salt bridge between D368 in Env and germline-encoded R71_HC_ in framework region 3 of the heavy chain (FWRH3, Fig. S8B) (10, 13, 49). 21N13 has R71_HC_ which is positioned to form a salt bridge with D368_Env_, in a highly similar way to how VRC01 and 1–18 interact (Fig. 5D). R71_HC_ is germline-encoded in both the inferred germline of 21N13 (macaque IGHV4–93) and that of VRC01 (human IGHV1–2) and 1–18 (human IGHV1–46), suggesting that this initial germline-encoded interaction might be important for the development of CD4bs-directed mAbs (Fig. S8A–B). Furthermore, 21N13 uses CDRL3 Y91_LC_, which is identical to the Y91_LC_ found in the shortened CDRL3 of VRC01-class bnAbs, to contact N279_Env_, while it employs T94_LC_ that is also found in 1–18 and 8ANC131 to contact N280_Env_ (Fig. 5D). A T57V_HC_ somatic mutation in 21N13 allows backbone interactions with S265_Env_, and has previously been shown to be necessary for breadth and potency of both VRC01 and 12A21 (Fig. S8A, C–D) (26).

### 21N13 can accommodate glycans at N197 and N276

We determined the crystal structure of 21N13 in complex with BG505 SOSIP as mentioned above (Fig. 5E, Fig. S8E). BG505 SOSIP has four glycans immediately neighboring the CD4bs that are removed in GT1.1 to facilitate binding and activation of CD4bs bnAb precursors, i.e. those at N197, N276, N386 and N463 (19). Overall, the crystal structure of 21N13 in complex with BG505 SOSIP was in agreement with the cryo-EM structure in complex with GT1.1 (Fig. 5F) and we did not observe substantive changes in Ab orientation or epitope when comparing the two structures or the bound *versus* unliganded 21N13 (Fig. 5F, Fig. S8E). 21N13 contacts two CD4bs glycans present on BG505 SOSIP: N197 with its FWRH3 (82 Å^2^ BSA) and N276 with its CDRL1 (65 Å^2^ BSA) (Fig. 5G), while no contacts are visible with N386 and N463. 21N13 does not have a short or flexible CDRL1 for N276 glycan accommodation as is found in VRC01-class Abs. Instead, the steeper approach angle and more helical structure of the CDRL1 of 21N13 prevents steric clashes with the N276 glycan (Fig. 5G). In fact, two CDRL1 residues, N30_HC_ and N31_HC_, are predicted to contact the N276 glycan (Fig. 5G). 21N13 contacts the N197 glycan through the FWRH3 region where a stretch of five residues appears to be mutated from germline “DTSKN” to “NIHER”. Within this stretch, K75E_HC_ directly contacts the N197 glycan, while S74H_HC_ hydrogen bonds with N195_Env_ (Fig. 5G). Thus, it appears that 21N13 acquired somatic mutations that establish contacts with glycans surrounding the CD4bs, which may have been selected for by exposure to these glycans on the BG505 SOSIP booster immunogen.

### nAb 12C11 binds a CD4bs epitope and contacts neighboring protomers

**Figure 6.**
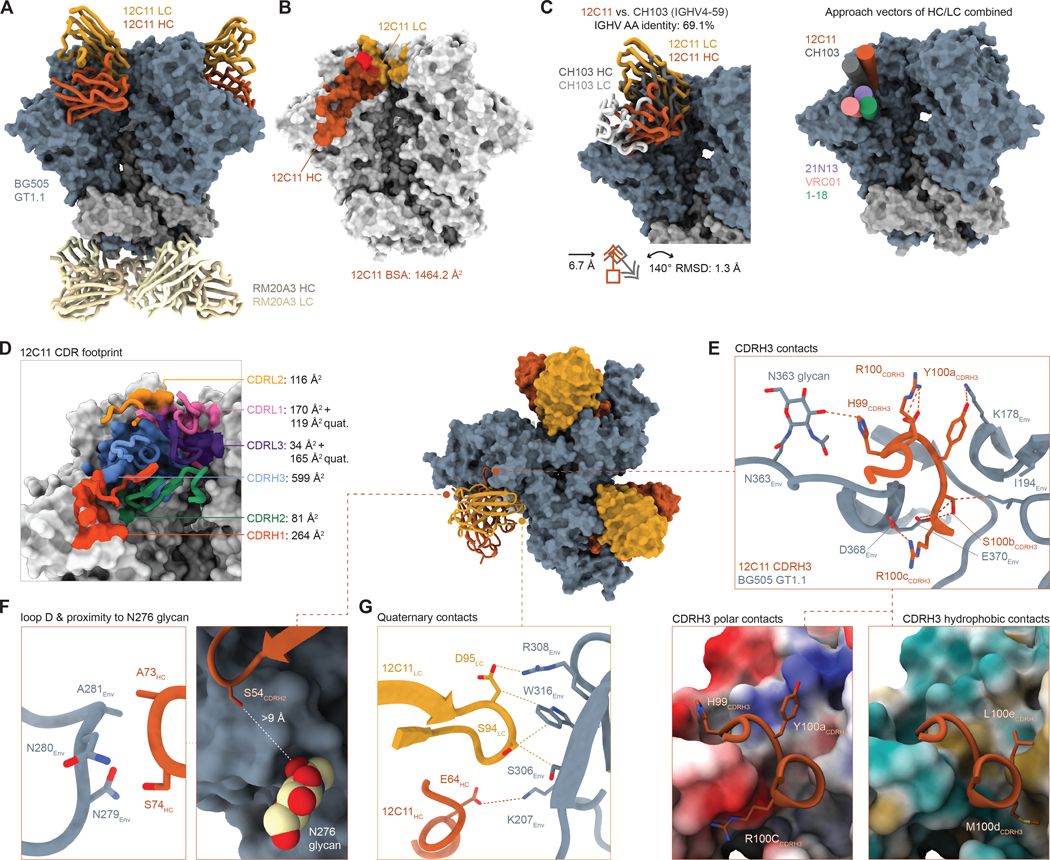
nAb 12C11 binds a quaternary-dependent apical CD4bs epitope. (A) Cryo-EM-derived atomic model of GT1.1 in complex with 12C11 and RM20A3 Fabs. (B) Footprint and buried surface area (BSA) of 12C11 Fab. The red shading indicates Env regions contacted by both heavy chain and light chain. (C) Superimposition of the GT1.1–12C11 structure with a published structure of Env-CH103 (PDB: 4JAN, left panel). The icons below the figure represent the mean displacement and rotation angle as well as the root mean square deviation (RMSD) of CH103 compared to 12C11. The right panel compares the approach vectors of NHP nAbs 21N13 (Fig. 5) and 12C11 with known CD4bs bnAbs CH103, VRC01 and 1–18. (D) Footprint of 12C11 CDRs onto the trimer. The numbers represent the buried surface area (BSA) contributed by each CDR. (E) Detailed interaction of the CDRH3-mediated contacts between GT1.1 and 12C11 (top panel) and an illustration of the polar CDRH3 contacts (bottom left panel) and the hydrophobic contacts (bottom right panel). (F) Inset showing the 12C11-loop D interaction (left panel) and the distance of the closest 12C11 residue to the N276 glycan (right panel). (G) Inset showing the quaternary interactions by 12C11 light chain (mustard) and 12C11 heavy chain (red) with the neighboring V3 region.

Compared to 21N13, nAb 12C11 targets a CD4bs epitope that is located closer to the trimer apex, yet contacts conserved CD4bs regions commonly targeted by CD4bs bnAbs, including C3 residues 365–372, loop D residues 278–281 and V5 residues 456–459 (Fig. 6A–B). Its main epitope on gp120 is contacted by the HC (1017 Å^2^ BSA) and LC (294 Å^2^ BSA), with the LC making extensive contacts with the V3 region on the neighboring protomer (174 Å^2^ BSA, Fig. 6B). Both its angle of approach and overall epitope are substantially different from those of VRC01-class and CH235-class bnAbs as well as 21N13, but more similar to human bnAb CH103 and the CDRH3-dominated class of CD4bs bnAbs (Fig. 6C). However, 12C11 has a flipped HC/LC orientation compared to CH103 (Fig. 6C) (16), and targets a more apical CD4bs epitope than CDRH3-dominated CD4bs bnAbs VRC13 and VRC16 (Fig. S8F) (15). Unlike most VRC01-class CD4bs bnAbs that predominantly use their CDRH2 for CD4bs contacts, the CDRH3 of 12C11 accounts for 64% of HC contacts and is mainly involved in binding the C4 region (Fig. 6B). Moreover, 12C11 is atypical in its ability of both HC and LC to contact a large, relatively conserved portion of the V2 loop (Fig. 6D).

The epitope of 12C11 is characterized by CDRH1 and CDRH3 contacts with C2 and C3 (S265-T372) and the C3 and C4 regions (D368, R419-Q428) (Fig. 6E). Its CDRH3 loop is involved in electrostatic contacts with D368_Env_ and E370_Env_, highly conserved CD4bs residues that are required for interaction with CD4 (Fig. 6D). Moreover, 12C11 accommodates the N-linked glycan at position 363 through H99_HC_ and contacts a conserved positive charge in the form of K174_Env_ in the V2 (~84% K/R) through Y100a_HC_ (Fig. 6E). Apart from these polar contacts, hydrophobic CDRH3 residues L100e_HC_ and M100d_HC_ interact with conserved hydrophobic Env residues V182_Env_, I194_Env_ and I423_Env_ (Fig. 6E, bottom panels), strengthening and adding to the V2 interaction. Moreover, 12C11 residues in the FWRH3 contact loop D residues N279_Env_, N280_Env_ and A281_Env_ (Fig. 6F, left panel). Due to their distance to the N276 glycan, 12C11 is likely not hindered by the N276 glycan, which might at least in part explain its breadth (Fig. 6F, right panel). The light chain of 12C11 predominantly contacts the V2 region on the main protomer, which contributes 294 Å^2^ BSA, and the C2 and V3 regions on an adjacent protomer contributing 174 Å^2^ BSA, with these contacts accounting for 37% of the total LC BSA (Fig. 6D,G). The main residues involved in quaternary contacts reside in the CDRL3 and involve both salt bridges and hydrophobic contacts to the C2/V3 residues in the adjacent protomer (Fig. 6G). Like 21N13, 12C11 extends a negatively charged, somatically mutated E64_HC_ towards highly conserved K207_Env_ as a quaternary interaction (Fig. 6G). Thus, GT1.1 priming followed by boosting with BG505 SOSIP or ConM SOSIP selected for at least two different CD4bs-specificities, both of which have the ability to accommodate the N276 glycan and neutralize diverse HIV-1 strains.

## Discussion

For many viral pathogens, (b)nAbs are the most important correlate of protection (51, 52). However, none of the eight HIV-1 vaccines that have reached efficacy trials in humans induced (b)nAbs against primary, neutralization-resistant (tier 2) viruses (8). Hence, strategies are being pursued that aim to mimic how bnAbs develop during natural HIV-1 infection, aided by structural understanding of the Env complex. Germline-targeting approaches represent such a strategy, one for which proof-of-concept was recently generated in a first-in-human clinical trial of the eOD-GT8 60-mer. That immunogen activated VRC01-class precursors in the large majority (97%) of healthy volunteers (7). However, none of the >200 authentic VRC01-class mAbs isolated from the trial bound to natively glycosylated Env trimers (7), implying that heterologous boosting with native-like Envs is likely necessary to guide B cell maturation. We hypothesized that priming with Env trimers might offer advantages over other germline-targeting using only regions of Env, as they impose structural constraints and will thus only select for B cells with the capacity to bind trimers.

Our initial characterization showed that a single GT1.1 immunization can effectively prime gl-VRC01-class B cells in a KI mouse model, with BCRs accumulating VRC01-class mutations over time. Moreover, B cells showed a propensity towards including a negatively charged amino acid in the CDRH3 of their BCR, thereby validating the design strategy of GT1.1. Isolated mAbs had a higher affinity for GT1.1 than gl-VRC01 and were able to bind to increasingly natively glycosylated trimers such as BG505 SOSIP N276D. However, neither the number nor the relative frequency of VRC01-class B cells in the KI mouse model are the same as in humans (frequency ~1 in 300.000 naive human B cells) (53).

The NHP immunization study yielded important insights on GT1.1 immunogenicity in a high-bar animal model. First, we show that GT1.1 priming followed by BG505 SOSIP boosting induces polyclonal specificities against the CD4bs and the FP, while GT1.1 priming followed by ConM SOSIP boosting led polyclonal specificities against the CD4bs and in one case to the trimer apex. This observation supports our hypothesis that priming with a native-like trimer offers advantages over gp120 core-based immunogens since it can initiate multiple desirable Ab lineages, which is likely imperative for a protective HIV-1 vaccine (8). We note here that GT1.1 was engineered to also bind to inferred germline precursors of V2-apex bnAbs (19), but it was not specifically modified to facilitate the induction of FP-specific antibodies. Nonetheless, several bnAb epitopes, including those proximate to the FP, are presented on GT1.1. Our second insight was that GT1.1 can prime neutralizing CD4bs responses, despite the fact that NHPs lack an IGHV1–2 ortholog and are therefore unable to induce *bona fide* VRC01-class responses. However, despite its low sequence similarity to known CD4bs bnAbs, nAb 21N13 targets the same conserved CD4bs residues in a highly convergent manner. The implication of our findings is that GT1.1 has the ability to prime a broad range of CD4bs precursors, not exclusively those of the VRC01-class.

Our results imply that priming with GT1.1 followed by BG505 SOSIP boosting is not sufficient to drive the development of nAbs and potency. In another KI mouse study, we showed that priming with a related germline-targeting trimer, GT1.2, followed by boosting with diverse shaping and polishing trimer triggered the selection of rare insertions and deletions (indels) in VRC01-class B cells similar to those found in human bnAbs. In contrast, GT1.2 priming followed by BG505 SOSIP boosting, similar to the regimen in this study, did not induce such indels (9). These findings support the inclusion of shaping and polishing immunogens in sequential immunization strategies, which are currently being validated *in vitro*.

In conclusion, we report the design and characterization of a germline-targeting HIV-1 vaccine immunogen, GT1.1. A limitation of our study is that GT1.1 was tested in KI mice and NHPs which have different BCR repertoires compared to humans. Therefore, GT1.1 is currently being assessed in a phase I human clinical trial (NCT04224701).

## Methods

### Study design

The main objective of this study was to evaluate GT1.1 as a priming immunogen in mice expressing a bnAb precursor as well as in non-human primates to evaluate its ability to prime bnAb lineages and mature them into nAbs. The number of animals used in each group was limited by availability and costs of the animals, but was deemed to be sufficient to detect any differences between groups. Animal experiments were conducted once, with group sizes ranging from three to six animals. Group assignment was done randomly; there was no blinding involved.

### Protein design

The BG505 SOSIP v4.1-GT1.1 trimer was conceived from the previously characterized BG505 SOSIP GT1 (19) by introducing a single amino acid substitution at position 275 (E275K) using the QuikChange mutagenesis kit (Agilent Technologies). Epitope knockouts, such as the CD4bs KO (GT1.1 D368R/N279A), glycan introductions such as GT1.1 N276 and glycan-deleted Envs, such as BG505 SOSIP N276D, were created using the same method. Proteins used for immunization did not have a protein tag, while those used for serological analyses and mAb characterization had a hexahistidine (his) tag. All constructs were cotransfected with furin into HEK293F cells (Invitrogen) and purified using a PGT145 or PGT151 affinity chromatography column six days after transfection as previously described (9, 17, 54).

### Glycopeptide analysis by LC-MS and UPLC

Glycopeptide analysis was performed as described in detail in (9). Briefly, GT1.1 were denatured 1 h in 50 mM Tris/HCl, pH 8.0 containing 6 M of urea and 5 mM of dithiothreitol (DTT) and reduced and alkylated by adding 20 mM iodoacetamide (IAA). The alkylated GT1.1 was buffer-exchanged into 50 mM Tris/HCl (pH 8.0) and digested overnight using trypsin or chymotrypsin (Promega). Next, digested peptides were separated using an EasySpray PepMap RSLC C18 column (75 μm × 75 cm) and data were extracted from the raw files (version 3.5, Protein Metrics Inc.) and evaluated manually. Glycans were categorized according to the composition detected.

Ultra-high performance liquid chromatography (UPLC) was performed by excising gel bands corresponding to GT1.1 and releasing N-linked glycans from the protein structure by overnight incubation at 37 °C with PNGaseF (2 μg enzyme in 100 μL H_2_O, New England BioLabs). Released glycans were fluorescently labeled and analyzed on a Waters Acquity H-Class UPLC instrument with a Glycan BEH Amide column (2.1 mm × 150 mm, 1.7 μM, Waters). Oligomannose-type glycans were quantified by digesting an aliquot of released and labelled glycans with endoglycosidase H (endoH) for 2 h at 37 °C 2 hours. EndoH was then removed from the sample using a PVDF membrane and the glycans were reanalyzed and compared to the untreated sample. Data was processed using Empower 3 software (Waters).

### Gel electrophoresis

Sodium dodecyl sulfate polyacrylamide gel electrophoresis (SDS-PAGE) and blue native PAGE (BN-PAGE) were performed as described elsewhere (55). In short, 2 μg of GT1.1 was loaded onto a 4–12% Tris-glycine gel (Invitrogen) or 4–12% Bis-Tris NuPage gel (Invitrogen) for SDS-PAGE and BN-PAGE, respectively. For SDS-PAGE, the gels were run for 1.5 h at 125 V using 50 mM MOPS/50 mM Tris pH 7.7 as running buffer. Gels were stained using the PageBlue Protein Staining Solution (Thermo Fisher). For BN-PAGE, the gels were run for 1.5 h at 200 V at 4 °C using anode buffer (20x NativePAGE buffer, Invitrogen, in MilliQ water) and cathode buffer (1% NativePAGE cathode buffer in anode buffer, Invitrogen) and stained using the Colloidal Blue kit (Life Technologies).

### Surface plasmon resonance (SPR)

The binding of (gl-)VRC01-class bnAbs to BG505 SOSIP v4.1-GT1 and GT1.1 was analyzed by SPR on a Biacore 3000 instrument (Cytiva) as previously described (9, 48). Briefly, His-tagged GT1 and GT1.1 trimers were captured in parallel flow cells, into which IgG of mature and gl-bnAbs was injected, starting at 1 μM in two- or four-fold dilutions. IgG-Env association was measured for 300 s and dissociation for 600 s at a flow rate of 50 uL/min. The sensorgram data were analyzed and validated with the BIAevaluation software v4.4.1 (Cytiva), as described in (9, 48). A bivalent model was fitted to the binding data after reference-channel and zero-analyte subtractions; χ^2^ values were in the range 0.52–2.7; average T values (1/s.e.m.) for measured kinetic constants and *R_max_* were >50; a simple Langmuir model gave unsatisfactory fits. The unit of the second-component on-rate constant, *k_on2_* (1/RUs) was converted to (1/Ms) by the formula, *k_on2_* (1/Ms) = *k_on2_* (1/RUs) **^.^** 1.5 **^.^** 10^5^ (g/mole) **^.^** 100 (56). Evidence against mass-transport limitation from global fits was strong: high (>> 10^8^, typically >10^15^) *k_t_* [RU/Ms] and low (<<10, typically <10^−2^) T(*k_t_*) values, thereby validating the kinetic parameters.

### Calcium flux assay

B cell activation experiments of Ramos B cells were performed as previously described (57, 58). In short, 4 × 10^6^ cells/mL in RPMI10 were loaded with 1.5 μM of the calcium indicator Indo-1 (Invitrogen) for 30 min at 37 °C, washed with Hank’s Balance Salt Solution supplemented with 2 mM CaCl_2_, followed by another incubation of 30 min at 37 °C. Antigen-induced Ca^2+^ influx of B cells was monitored on a LSR Fortessa (BD Biosciences) by measuring the 379/450 nm emission ratio of Indo-1 fluorescence upon UV excitation. Following 30 s of baseline measurement, aliquots of 1 × 10^6^ cells/mL were then stimulated for 210 s at RT with 1, 10 or 100 μg/mL of GT1 or GT1.1 trimers. Ionomycin (Invitrogen) was added to a final concentration of 1 μg/μL to determine the maximum Indo-1-fluorescence. Kinetic analyses were performed using FlowJo v10.7.

### Generation of a knock-in (KI) mouse model

gl-VRC01 KI mice were kindly provided by Dr. Fred Alt and generated as described in (25). Briefly, gl-VRC01 KI mice utilize an obligate human germline IGKV3–20 and the mature CDRL3-containing human Jκ1 in their light chain. The heavy chain encodes obligately expressed human JH2 and a facultatively rearranging human IGHV1–2*02 genomic element, which allows the expression of a heterogeneous, and mostly endogenous, murine IGHD gene repertoire. Mouse breeding was performed at Massachusetts General Hospital (MGH), and experimental procedures were performed at the Ragon Institute of MGH, MIT and Harvard following protocols approved by the Institutional Animal Care and Use Committee (IACUC) of Harvard University and the MGH (2016N000286 and 2016N000022) and conducted in accordance with the regulations of the Association for Assessment and Accreditation of Laboratory Animal Care (AAALAC) International.

### Mouse immunizations

Immunogens (GT1, GT1.1 or GT1.2 trimers or GT1.1-I53–50 NPs at 10 μg/mouse) were diluted in PBS at a volume of 100 μL per mouse and mixed with 100 μL of poly I:C (Invivogen, at 60 μg/mouse). Formulated immunogens were injected intraperitoneally. In the adjuvant comparison studies (Fig. S3), the immunogens were mixed with 2% Alyhydrogel adjuvant, poly I:C (60 μg/mouse) or adjuplex (1% of adjuplex in 200 μL mixture/mouse).

### Flow cytometry on mouse cells

At days 8, 16 or 44 post-immunization, spleens were harvested from mice. Splenocytes were isolated by mechanical dissociation using a cell strainer, red blood cells were removed by ACK lysis buffer (Gibco). Isolated cells were incubated with anti-mouse CD16/32 antibody (BD Biosciences) to block the B cell Fc receptors and Live/Dead Blue (Thermo Fisher) according to manufacturer’s instructions. Subsequently, the cells were stained with GT1.1 probes and surface antibodies. Cells were analyzed by an LSRFortessa (BD Biosciences) and sorted using a FACS Aria II (BD Biosciences). Data were analyzed by FlowJo software (Tree Star). Single dry-sorted cells were frozen on dry ice in 96-well plates and stored at −80°C until B cell receptor sequencing.

### BCR sequencing

Frozen, sorted B cells were lysed using NP-40 and RNA encoding the variable regions of the heavy and light chains of IgG were synthesized to cDNA using SuperScript III Reverse Transcriptase (Thermo Fisher) according to manufacturer’s instructions. Nested PCR reactions consisting of PCR-1 and PCR-2 were performed using HotStarTaq (QIAGEN), 10 mM dNTPs (Thermo Fisher) and cocktails of murine IgG- and IgK-specific primers and thermocycling conditions described previously (59) as well as knockin-sequence-specific primers, including HC forward primers (5’-ATGGACTGGACCTGGAGGAT-3’ for PCR-1 and 5’-ACAGGAGCCCACTCCCAGGTGCAG-3’ for PCR-2) and LC forward primers 5’-CGCAGCTTCTCTTCCTCCTG-3’ for PCR-1 and 5’-ACTCTGGCTCCCAGATACCA-3’ for PCR-2) used previously (25). PCR products were run on a 2% agarose gel with SYBR Safe (Thermo Fisher) and wells with correct bands were analyzed by Sanger sequencing (GeneWiz). HC products were sequenced using the HC reverse primer from PCR-2 (5’-GCTCAGGGAARTAGCCCTTGAC-3’) and the LC was sequenced using the LC reverse primer (5’-TGGGAAGATGGATACAGTT-3’) from PCR-2. Reads were quality-checked, trimmed, aligned and analyzed using the Geneious software (Biomatters). IMGT/V-QUEST (http://www.imgt.org) was used for mouse/human Ig gene assignments, and the Immcantation framework was used to assign clonal groups with a 0.1 threshold (60).

### Mouse serum ELISAs

96-well plates were coated overnight at 4 °C with GT1.1. The coated plates were washed 5 times with 0.05% Tween-20 in PBS, blocked with 100 μL of 3% BSA in PBS for 1 h at RT, and washed prior to incubation with 1:3 serially diluted serum samples for 1 h at RT. The wells were washed and incubated with Alkaline Phosphatase AffiniPure Goat Anti-Mouse IgG (Jackson Immuno Research) at 1:1000 in PBS with 0.5% BSA for 1 h at RT. p-Nitrophenyl phosphate dissolved in ddH2O (50 μL/well, 25 min at RT) was used for detection. The absorbance at 405 nm was determined with a Synergy Neo2 plate reader (BioTek).

### Production of monoclonal antibodies (mAbs)

Sequences obtained were cloned into an IgG1 expression vector and transfected in HEK293F cells (Invitrogen, cat no. R79007) at a density of ~1 million cells/mL in a 1:1 HC:LC ratio by addition of PEImax (1 mg/L). Five days after transfection, cells were harvested by centrifugation (3000G, 30 min) and filtered using 0.22 μm SteriTop filters (Merck). Supernatants were incubated overnight at 4 °C with 2 mL protein A beads (Thermo Fisher)/L supernatant. The following day, the supernatant was run through a column, washed twice with PBS and antibodies were eluted with 0.1 M glycine (pH 2.5) into 1 M Tris (pH 8.7) in a 9:1 ratio to immediately buffer the eluted antibodies. The eluate was buffer-exchanged to PBS using 100 kDa VivaSpin columns (Sartorius) and antibody concentration was measured using a NanoDrop 2000 (Thermo Fisher).

### Biolayer interferometry

BLI experiments were performed as described previously (61, 62). Briefly, mAbs (10 μg/mL) were diluted in running buffer (PBS, 0.02% Tween 20, and 0.1% bovine serum albumin) and subsequently loaded onto protein A sensors (ForteBio) until wavelength reached 2 nm. After the sensors were dipped in a well containing running buffer to remove excess antibody, the sensors loaded with antibodies were dipped into wells containing the protein of interest (10 μg/mL) and allowed binding for 300 s to measure association. Next, the sensors were dipped in a well containing running buffer for 300 s to measure dissociation.

### mAb ELISAs

His-tagged BG505 SOSIP.664, GT1.1, and ConM trimers were produced by transient transfection of 293F cells as described and purified by PGT145-affinity chromatography (63). Trimers and mAbs were characterized by coating 2 μg/mL of his-tagged Env in casein (Thermo Fisher) onto 96-well Ni-NTA ELISA plates (Qiagen) and incubating for 2 h at room temperature. Next, the plates were washed thrice with TBS and incubated with three-fold serial dilutions of mAbs in casein (Thermo Fisher) for 2 h at room temperature before being washed thrice again with TBS. Horseradish peroxidase-labeled goat anti-human secondary antibody was allowed binding for 1 h at room temperature. The plates were washed five times with TBS/0.05% Tween-20 and developed using develop solution (1% 3,3’, 5,5’-tetramethylbenzidine (Sigma-Aldrich), 0.01% hydrogen peroxide, 100 mM sodium acetate and 100 mM citric acid). After the colorimetric endpoint was reached by eye, reactions were stopped by adding 0.8 M sulfuric acid in a ratio of 2:1 and colorimetric changes were measured on a SPECTROstar Nano (BMG Labtech) at a wavelength of 450 nm. mAbs from NHP studies (Fig. 3 onwards) were characterized in the same fashion.

### mAb neutralization assays

Briefly, neutralization experiments were set up to measure the ability of mAbs to reduce luciferase gene expression in adherent TZM-bl cells. These cells have been modified to include firefly luciferase genes, which are under control of an HIV-1 promotor. Virus was incubated with serially diluted mAbs for 1 h at 37 °C. 400 nM saquinavir and 40 μg/mL DEAE were added to the cells before addition of the mAb/virus mixture. Cells were grown for three days at 37 °C, after which cells were lysed and luciferase activity was measured using Bright-Glo (Promega) substrate on a GloMax Discover machine. mAbs from NHP studies (Fig. 3 onwards) were characterized in the same fashion.

### Non-human primate immunizations

Eighteen healthy, uninfected (negative for simian immunodeficiency virus; simian retrovirus; simian T lymphotropic virus) male adult Indian rhesus macaques (*Macaca mulatta)* aged 4–7 years were used for these immunization studies in three groups of 6 male rhesus macaques. All animals were housed and maintained at the New Iberia Research Center (NIRC) of the University of Louisiana at Lafayette in accordance with the rules and regulations of the Committee on the Care and Use of Laboratory Animal Resources. The study protocol was reviewed and approved by the University of Louisiana at Lafayette Institutional Animal Care and Use Committee (Protocol #2016–8787-072). Group 1 (BG505 SOSIP) animals were immunized with BG505 SOSIP.664 (100 μg) at weeks 0, 8 and 25 adjuvanted with GLA-LSQ (25 μg GLA/10 μg QS21) delivered subcutaneously. Subsequent immunizations were performed with BG505 SOSIP.664 (100 μg) adjuvanted with TLR7/8 ligand 3M-052 (30 μg 3M052/500 μg alum) at weeks 43, 52 and 69 (Fig. 3A). Group 2 and 3 animals (GT1.1/BG505 SOSIP and GT1.1/ConM SOSIP) were immunized at weeks 0 and 8 with BG505 SOSIP GT1.1 (100 μg), adjuvanted with GLA-LSQ as outlined above, followed by boosting with BG505 SOSIP.664 or ConM SOSIP (100 μg) at the same time points as outlined above. Immunizations performed at weeks 43, 52 and 69 were adjuvanted with 3M052-AF/alum and delivered subcutaneously and bilaterally as two doses of 50 μg.

### Serum ELISAs

ELISA to detect anti-trimer antibodies was performed as described previously with minor modifications (54). His-tagged SOSIP trimer was coated onto Ni-NTA HisSorb Plates (Qiagen) at 1.5 μg/mL in TBS (150mM NaCl, 20 mM Tris). Background wells were incubated with TBS only. Wells were washed three times with TBS, blocked with TBS/2% non-fat milk powder (TBS-M), and washed again with TBS before addition of serial dilutions of macaque sera (or, as a positive control, the 2G12 bnAb) in TBS-M/20% sheep serum. The plates were washed with TBS and horseradish peroxidase-labeled goat anti-rhesus immunoglobulin G (IgG) (Southern Biotech) was added in TBS-M; the plates were washed five times with PBS/0.05% Tween-20. Finally, colorimetric detection was performed with the 1-Step Ultra TMB-ELISA substrate (Thermo Scientific). Background OD450 was subtracted from each of the replicate test OD450 values to yield net OD450. OD450 readings were plotted vs. log10 sample dilution and fitted by a four-parameter sigmoidal dose-response function, the lower constraint set to 0 (GraphPad Prism v9.4.1). Mean midpoint effective-dilution (ED50) values from at least two independent ELISAs per sample are reported.

### Serum neutralization

Neutralizing antibodies were measured as a function of reductions in luciferase (Luc) reporter gene expression after a single round of infection in TZM-bl cells (64, 65). TZM-bl cells (also called JC57BL-13) were obtained from the NIH AIDS Research and Reference Reagent Program, as contributed by John Kappes and Xiaoyun Wu. This cell line contains integrated reporter genes for firefly luciferase and E. coli beta-galactosidase under control of an HIV-1 LTR (65–67). Briefly, a pre-titrated dose of virus was incubated with serial 3-fold dilutions of heat-inactivated (30 min at 56 °C) serum samples in duplicate in a total volume of 150 μL for 1 h at 37 °C in 96-well flat-bottom culture plates. Freshly trypsinized cells (10,000 cells in 100 μL of growth medium containing 75 μg/mL DEAE dextran) were added to each well. After 48 hours of incubation, 100 μL of cells was transferred to a 96-well black solid plate (Costar) for measurements of luminescence using the Britelite Luminescence Reporter Gene Assay System (PerkinElmer Life Sciences). Neutralization titers (ID_50_s) are the dilution (serum/plasma samples) at which relative luminescence units (RLU) were reduced by 50% compared to virus control wells after subtraction of background RLUs. Assay stocks of molecularly cloned Env-pseudotyped viruses were prepared by transfection in 293T/17 cells (American Type Culture Collection) and titrated in TZM-bl cells as described in (64, 65). This assay has been formally optimized and validated (68) and was performed in compliance with Good Clinical Laboratory Practices, including participation in a formal proficiency testing program (69). The neutralization assays to determine the tier phenotype of GT1.1 pseudovirus were performed with purified IgG samples with a stock concentration of 12.5 mg/mL.

### Electron microscopy-based polyclonal epitope mapping (EMPEM)

The details of serum and sample preparation to obtain polyclonal Fabs for electron microscopy were previously described in (70). Briefly, IgG was isolated using Protein A (Cytiva) from 1–4 mL NHP sera (drawn at week 54 post first immunization). Papain (Sigma Aldrich) was used to digest IgG to antigen-binding fragments (Fab). Sera of animal A12N155 at week 10, 27, 45 and 54 were processed the same way as described above. Trimer-Fab complexes were prepared and incubated overnight by mixing 15 μg of BG505.664 SOSIP or BG505 GT1.1 trimer with 1 mg of Fab mixture (containing Fc and residual papain). On the next day, the complexes were purified using a Superdex 200 Increase 10/300 GL gel filtration column (Cytiva). Purified complexes were concentrated and diluted to a final concentration of 0.03 mg/mL, which were adsorbed on glow-discharged carbon coated copper mesh grids and stained with 2% (w/v) uranyl formate. Electron microscopy images were collected on an FEI Tecnai Spirit T12 equipped with an FEI Eagle 4k x 4k CCD camera (120 keV, 2.06 Å/pixel) or a n FEI Tecnai TF20 equipped with a TVIPS TemCam F416 CMOS camera (120 keV, 1.67 Å/pixel) and processed using Relion 3.0 (71) following standard 2D and 3D classification procedures. UCSF ChimeraX (72) was used to generate the composite maps, and the representative maps with identified epitopes have been deposited to the Electron Microscopy Data Bank.

### Flow cytometry on NHP cells

Cryopreserved PBMC samples from six animals (A11N160, A12N074, A12N115, A12N108, A13N146, A12N125) were thawed in 9 mL IMDM with 10% fetal calf serum (FCS) and 20 μL benzonase and washed twice with PBS. Cells were stained in 100 uL PBS with antibody mix, incubated in the dark for 30 mins at 4 °C and washed with PBS. Cells were then similarly stained with antigen probes and washed three times in IMDM with 10% FCS. Cells were strained through a 70 μm filter. 7-AAD live/dead stain was added before sorting. The antibodies used were: CD14 (Clone M5E2, Biolegend), CD16 (Clone 3G8, BD Biosciences), CD56 (B159, BD Biosciences), CD3 (Clone SP34–2, BD Biosciences), CD4 (Clone L200, BD Biosciences), CD19 (Clone J3–119, Beckman Coulter) CD20 (Clone 2H7 BD Biosciences), IgD (polyclonal, Southern Biotech, IgG (Clone G18–145). CD14, CD16, CD56 were on the same fluorophore and excluded as a dump channel. Identical biotinylated GT1.1 probes were conjugated to two different streptavidin fluorophores. IgG+ memory B cells which bound both GT1.1 probes were sorted for culture.

### NHP B cell microneutralization and sequencing

Single probe-positive B cells were sorted at 1 cell per well in 384-well cell culture plates (Thermo Fisher) and cultured as described in (73, 74). Briefly, each well contained 50 μL of culture medium (IMDM with GlutaMAX, Thermo Fisher) with 10% heat inactivated FCS (Thermo Fisher), 100 U/mL IL-2 (Millipore-Sigma), 50 ng/mL IL-21 (Thermo Fisher), 50 ng/mL IL-4 (Miltenyi), 50 ng/mL BAFF (Millipore-Sigma), 2 μg/mL CpG ODN2006 (Miltenyi), 1X MycoZap Plus-PR (Lonza), and 5000 3T3-msCD40L feeder cells per well. Cultures were incubated undisturbed for 14 days at 37 °C in 5% CO_2_ for cell expansion and antibody secretion. Culture supernatants were then harvested for microneutralization. Microneutralization assays were performed as described in (75) in duplicate using TZM-bl reporter cells and germline bnAb sensitive virus, 426c.TM.293S/GnT1^–^. Wells that showed neutralization were selected for RT-PCR. Additional wells were chosen based on factors including B cell growth by microscopy, strong *ex vivo* probe binding, or negative neutralization of 426c.TM.293S/GnT1^–^ pseudovirus. Multiplex RT-PCR was used to amplify heavy and light chain immunoglobulin variable regions (76). PCR products were sequenced by Azenta (Genewiz) and analyzed using Geneious Prime, IgBLAST, and the rhesus macaque immunoglobulin database published by Ramesh *et al.* (77) as well as the Karolinska immunoglobulin gene database (KIMDB) (78).

### Cryoelectron microscopy (cryo-EM) grid preparation

21N13, 21M20 and 12C11 IgG were digested into Fabs using papain (Sigma Aldrich). 0.2 mg of BG505 GT1.1 was incubated overnight at room temperature with either 0.3 mg each of 21N13 and 21M20, or 0.3 mg of 12C11 Fab. Additionally, 0.3 mg of RM20A3 Fab (an NHP antibody specific to the base of BG505-derived trimers) was added to each complex to help with orientation sampling. The trimer-Fab complexes were then purified using a HiLoad 16/600 Superdex 200 pg (Cytiva) gel filtration column. The complexes were then concentrated to between 4–8 mg/mL for application onto cryoEM grids. Cryo grids were prepared using a Vitrobot Mark IV (Thermo Fisher). The temperature was set to 4 °C and humidity was maintained at 100% during the freezing process. The blotting force was set to 1 and wait time was set to 10 s. Blotting time was varied from 3 to 6 s. Detergents lauryl maltose neopentyl glycol (LMNG; Anatrace) or n-Dodecyl-β-D-Maltoside (DDM; Anatrace) at final concentrations of 0.005 or 0.06 mM, respectively, were used for freezing. Quantifoil R 1.2/1.3 (Cu, 300-mesh; Quantifoil Micro Tools GmbH) grids were used and treated with Ar/O2 plasma (Solarus plasma cleaner, Gatan) for 8 s before sample application. 0.5 μL of detergent was mixed with 3.5 μL of samples and 3 μL of the mixture was immediately loaded onto the grid. Following blotting, the grids were plunge-frozen into liquid nitrogen-cooled liquid ethane.

### Cryo-EM data collection

Cryo grids were loaded into an FEI Glacios 2 electron microscope (Thermo Fisher) operating at 200 kV. Exposure magnification was set to 190,000x with a pixel size at the specimen plane of 0.725 Å. EPU software (Thermo Fisher) was used for automated data collection. Micrograph movie frames were motion and CTF corrected using cryoSPARC Live (79). The remaining data processing was performed in cryoSPARC. Particle picking was performed using blob picker initially followed by template picker. During extraction particles were downscaled to 1.044 Å/pix (21N13+21M20+RM20A3 complex) or 1.009 Å/pix (12C11+RM20A3 complex) to reduce box size and increase speed of downstream jobs. Multiple rounds of 2D classification and 3D ab-initio reconstruction were performed prior to 3D non-uniform refinement with global CTF refinement. A total of 242,098 particles went into the final, asymmetric 3D refinement of the 21N13+21M20+RM20A3 complex, with a Fourier Shell Correlation (FSC) resolution (0.143 threshold) of 2.9 Å. For the 12C11+RM20A3 complex, a total of 456,166 particles went into the final C3-symmetric 3D refinement with both global and local CTF refinements, and the global resolution estimated by FSC 0.143 was 2.8 Å. Final data collection and processing stats are summarized in Table S5. Model building was performing by docking homology models of trimer and Fab Fv in UCSF Chimera, manually building and refinement in Coot 0.9.8 (80) and real space refinement using Rosetta (81) and Phenix (82). Final models were validated using MolProbity and EMRinger in the Phenix suite, and statistics are summarized in Supplementary Table X. All maps and models have been deposited to the Electron Microscopy Data Bank and Protein Data Bank, respectively with accession codes summarized in Table S5.

### Crystallization and x-ray data collection

BG505 SOSIP was complexed with 21N13 Fab and the Fv domain of 35O22 (in a 1:3.5:3.5 molar ratio of Env trimer:Fab:scFv) for 30 mins at RT. The complex mixture was then digested with endoH glycosidase (New England Biolabs) at 37 °C for 30 mins to remove glycans accessible to endoH trimming. The digested complex was purified by size exclusion using a Superdex 200 16/600 column (GE Healthcare) and concentrated to 9.2 mg/mL before being subjected to crystallization screening at both 4 and 20 °C using a high-throughput CrystalMation robotic system (Rigaku). High quality crystals were obtained via sitting droplets in a precipitant condition of 0.1 M sodium acetate, pH 5.5, and 1.75 M ammonium sulfate at 20 °C. Crystals were harvested with 20% ethylene glycol as cryoprotectant and were immediately cryo-cooled by insertion into liquid nitrogen. The diffraction data were collected at SSRL beamline 12–1. For the unliganded structure of 21N13, the purified 21N13 Fab was concentrated to 10 mg/mL for crystallization screening as above. Crystals were obtained in sitting droplets in a precipitant condition of 0.237 M potassium thiocyanate, and 10% (w/v) polyethylene glycol 3350 at 20 °C. Crystals were harvested with 10% glycerol as cryoprotectant and were immediately cryo-cooled by insertion into liquid nitrogen. The diffraction data were collected at the Advanced Photon Source (APS) beamline 23ID-D.

### X-ray crystallographic structure determination and refinement

The crystals of BG505–21N13Fab-35O22scFv complex diffracted to 4.7 Å, while those of the unliganded 21N13 Fab diffracted to 2.5 Å. The diffraction data sets were indexed, integrated, and scaled using HKL2000 (83) in P2_1_3 and C121 space groups, respectively. The structure of 21N13 Fab was solved by molecular replacement with Phaser (84) using the PDB structure of F105 Fab (PDB 1U6A) as a search model. For the complex, the solved 21N13 Fab, BG505.664 gp140 (PDB 5CEZ) and 35O22 scFv (PDB 6MTJ) structures were used as search models. The two structures were then built and refined via different iterations of Coot (85) and Phenix (86). Env was numbered according to the HXB2 system. Final data collection and processing stats are summarized in Table S6.

### Analysis of the trimer-Ab interface

All analyses were done with UCSF ChimeraX (1.5) or PyMOL 2.4. The buried surface area was calculated in ChimeraX, using the measure_buriedarea command. The approach vectors as depicted in Fig. 6C were determined in ChimeraX by first generating a centroid of the Fv domain of the mAb. Then, we generated a centroid of the epitope of that particular mAb, defined as all Env residues within 5 Å. The approach vector was then determined by drawing an axis through these two centroids. The angle comparisons between 21N13, VRC01 and 1–18 in Fig. 5 as well as between 12C11 and CH103 were performed in PyMOL using the angle_between_domains command. The values reported in the figures relate to the rotation angle of the mAbs and the displacement of the center of mass, compared to 21N13 and 12C11, respectively.

### Statistical analysis

The statistical analyses performed are indicated in the respective figure legends.

### Data analysis and visualization

Analyses were performed in GraphPad Prism 9.1, RStudio (2022.07.2, build 576) using R4.2.2 or using the manufacturers’ software as indicated in the relevant paragraphs in the Methods section. Plots in RStudio were made using the ggplot2 package as part of the tidyverse package.

## Supplementary Material

Supplementary Figures and Tables

## Data Availability

The mAbs generated in this study will be available under an MTA with Amsterdam UMC and mAb sequences can be found in Table S3. This paper does not report original code. The cryo-EM structures presented in this manuscript can be found in the Protein Data Bank under accession codes codes 8SW3, 8SW4, and the Electron Microscopy Data Bank under accession codes EMD-40796 and EMD-40797. The X-ray crystal structures can be found in the Protein Data Bank under accession codes 8D01 and 8D0Y. All other data needed to support the conclusions of the paper are present in the paper or the Supplementary Materials.

## References

[1] Julg B, Liu P-T, Wagh K, Fischer WM, Abbink P, Mercado NB, Whitney JB, Nkolola JP, McMahan K, Tartaglia LJ, Borducchi EN, Khatiwada S, Kamath M, LeSuer JA, Seaman MS, Schmidt SD, Mascola JR, Burton DR, Korber BT, Barouch DH, Protection against a mixed SHIV challenge by a broadly neutralizing antibody cocktail. Sci. Transl. Med. 9, (2017). 10.1126/scitranslmed.aao4235 PMC5747528 28931655

[2] Sok D, Burton DR, Recent progress in broadly neutralizing antibodies to HIV. Nat. Immunol. 19, 1179–1188 (2018). 30333615 10.1038/s41590-018-0235-7 PMC6440471

[3] Corey L, Gilbert PB, Juraska M, Montefiori DC, Morris L, Karuna ST, Edupuganti S, Mgodi NM, deCamp AC, Rudnicki E, Huang Y, Gonzales P, Cabello R, Orrell C, Lama JR, Laher F, Lazarus EM, Sanchez J, Frank I, Hinojosa J, Sobieszczyk ME, Marshall KE, Mukwekwerere PG, Makhema J, Baden LR, Mullins JI, Williamson C, Hural J, McElrath MJ, Bentley C, Takuva S, Gomez Lorenzo MM, Burns DN, Espy N, Randhawa AK, Kochar N, Piwowar-Manning E, Donnell DJ, Sista N, Andrew P, Kublin JG, Gray G, Ledgerwood JE, Mascola JR, Cohen MS, Two Randomized Trials of Neutralizing Antibodies to Prevent HIV-1 Acquisition. N. Engl. J. Med. 384, 1003–1014 (2021). 33730454 10.1056/NEJMoa2031738 PMC8189692

[4] Hraber P, Seaman MS, Bailer RT, Mascola JR, Montefiori DC, Korber BT, Prevalence of broadly neutralizing antibody responses during chronic HIV-1 infection. AIDS 28, 163–169 (2014). 24361678 10.1097/QAD.0000000000000106 PMC4042313

[5] van Schooten J, van Gils MJ, HIV-1 immunogens and strategies to drive antibody responses towards neutralization breadth. Retrovirology 15, 74 (2018). 30477581 10.1186/s12977-018-0457-7 PMC6260891

[6] Burton DR, Hangartner L, Broadly Neutralizing Antibodies to HIV and Their Role in Vaccine Design. Annu. Rev. Immunol. 34, 635–659 (2016). 27168247 10.1146/annurev-immunol-041015-055515 PMC6034635

[7] Leggat DJ, Cohen KW, Willis JR, Fulp WJ, deCamp AC, Kalyuzhniy O, Cottrell CA, Menis S, Finak G, Ballweber-Fleming L, Srikanth A, Plyler JR, Schiffner T, Liguori A, Rahaman F, Lombardo A, Philiponis V, Whaley RE, Seese A, Brand J, Ruppel AM, Hoyland W, Yates NL, Williams LD, Greene K, Gao H, Mahoney CR, Corcoran MM, Cagigi A, Taylor A, Brown DM, Ambrozak DR, Sincomb T, Hu X, Tingle R, Georgeson E, Eskandarzadeh S, Alavi N, Lu D, Mullen T-M, Kubitz M, Groschel B, Maenza J, Kolokythas O, Khati N, Bethony J, Crotty S, Roederer M, Hedestam GBK, Tomaras GD, Montefiori D, Diemert D, Koup RA, Laufer DS, McElrath MJ, McDermott AB, Schief WR, Vaccination induces HIV broadly neutralizing antibody precursors in humans. Science 378, eadd6502 (2022). 36454825 10.1126/science.add6502 PMC11103259

[8] Haynes BF, Wiehe K, Borrow P, Saunders KO, Korber B, Wagh K, McMichael AJ, Kelsoe G, Hahn BH, Alt F, Shaw GM, Strategies for HIV-1 vaccines that induce broadly neutralizing antibodies. Nat. Rev. Immunol. 23, 142–158 (2023). 35962033 10.1038/s41577-022-00753-w PMC9372928

[9] Caniels TG, Medina-Ramírez M, Zhang J, Sarkar A, Kumar S, LaBranche A, Derking R, Allen JD, Snitselaar JL, Capella-Pujol J, Sánchez IDM, Yasmeen A, Diaz M, Aldon Y, Bijl TPL, Venkatayogi S, Martin Beem JS, Newman A, Jiang C, Lee W-H, Pater M, Burger JA, van Breemen MJ, de Taeye SW, Rantalainen K, LaBranche C, Saunders KO, Montefiori D, Ozorowski G, Ward AB, Crispin M, Moore JP, Klasse PJ, Haynes BF, Wilson IA, Wiehe K, Verkoczy L, Sanders RW, Germline-targeting HIV-1 Env vaccination induces VRC01-class antibodies with rare insertions. Cell Rep Med 4, 101003 (2023). 37044090 10.1016/j.xcrm.2023.101003 PMC10140475

[10] Zhou T, Georgiev I, Wu X, Yang Z-Y, Dai K, Finzi A, Kwon YD, Scheid JF, Shi W, Xu L, Yang Y, Zhu J, Nussenzweig MC, Sodroski J, Shapiro L, Nabel GJ, Mascola JR, Kwong PD, Structural basis for broad and potent neutralization of HIV-1 by antibody VRC01. Science 329, 811–817 (2010). 20616231 10.1126/science.1192819 PMC2981354

[11] Scheid JF, Mouquet H, Ueberheide B, Diskin R, Klein F, Oliveira TYK, Pietzsch J, Fenyo D, Abadir A, Velinzon K, Hurley A, Myung S, Boulad F, Poignard P, Burton DR, Pereyra F, Ho DD, Walker BD, Seaman MS, Bjorkman PJ, Chait BT, Nussenzweig MC, Sequence and structural convergence of broad and potent HIV antibodies that mimic CD4 binding. Science 333, 1633–1637 (2011). 21764753 10.1126/science.1207227 PMC3351836

[12] Huang J, Kang BH, Ishida E, Zhou T, Griesman T, Sheng Z, Wu F, Doria-Rose NA, Zhang B, McKee K, O’Dell S, Chuang G-Y, Druz A, Georgiev IS, Schramm CA, Zheng A, Joyce MG, Asokan M, Ransier A, Darko S, Migueles SA, Bailer RT, Louder MK, Alam SM, Parks R, Kelsoe G, Von Holle T, Haynes BF, Douek DC, Hirsch V, Seaman MS, Shapiro L, Mascola JR, Kwong PD, Connors M, Identification of a CD4-Binding-Site Antibody to HIV that Evolved Near-Pan Neutralization Breadth. Immunity 45, 1108–1121 (2016). 27851912 10.1016/j.immuni.2016.10.027 PMC5770152

[13] Schommers P, Gruell H, Abernathy ME, Tran M-K, Dingens AS, Gristick HB, Barnes CO, Schoofs T, Schlotz M, Vanshylla K, Kreer C, Weiland D, Holtick U, Scheid C, Valter MM, van Gils MJ, Sanders RW, Vehreschild JJ, Cornely OA, Lehmann C, Fätkenheuer G, Seaman MS, Bloom JD, Bjorkman PJ, Klein F, Restriction of HIV-1 Escape by a Highly Broad and Potent Neutralizing Antibody. Cell 180, 471–489.e422 (2020). 32004464 10.1016/j.cell.2020.01.010 PMC7042716

[14] Bonsignori M, Zhou T, Sheng Z, Chen L, Gao F, Joyce MG, Ozorowski G, Chuang G-Y, Schramm CA, Wiehe K, Alam SM, Bradley T, Gladden MA, Hwang K-K, Iyengar S, Kumar A, Lu X, Luo K, Mangiapani MC, Parks RJ, Song H, Acharya P, Bailer RT, Cao A, Druz A, Georgiev IS, Kwon YD, Louder MK, Zhang B, Zheng A, Hill BJ, Kong R, Soto C, Program NCS, Mullikin JC, Douek DC, Montefiori DC, Moody MA, Shaw GM, Hahn BH, Kelsoe G, Hraber PT, Korber BT, Boyd SD, Fire AZ, Kepler TB, Shapiro L, Ward AB, Mascola JR, Liao H-X, Kwong PD, Haynes BF, Maturation Pathway from Germline to Broad HIV-1 Neutralizer of a CD4-Mimic Antibody. Cell 165, 449–463 (2016). 26949186 10.1016/j.cell.2016.02.022 PMC4826291

[15] Zhou T, Lynch RM, Chen L, Acharya P, Wu X, Doria-Rose NA, Joyce MG, Lingwood D, Soto C, Bailer RT, Ernandes MJ, Kong R, Longo NS, Louder MK, McKee K, O’Dell S, Schmidt SD, Tran L, Yang Z, Druz A, Luongo TS, Moquin S, Srivatsan S, Yang Y, Zhang B, Zheng A, Pancera M, Kirys T, Georgiev IS, Gindin T, Peng H-P, Yang A-S, Program NCS, Mullikin JC, Gray MD, Stamatatos L, Burton DR, Koff WC, Cohen MS, Haynes BF, Casazza JP, Connors M, Corti D, Lanzavecchia A, Sattentau QJ, Weiss RA, West AP Jr., Bjorkman PJ, Scheid JF, Nussenzweig MC, Shapiro L, Mascola JR, Kwong PD, Structural Repertoire of HIV-1-Neutralizing Antibodies Targeting the CD4 Supersite in 14 Donors. Cell 161, 1280–1292 (2015). 26004070 10.1016/j.cell.2015.05.007 PMC4683157

[16] Liao H-X, Lynch R, Zhou T, Gao F, Alam SM, Boyd SD, Fire AZ, Roskin KM, Schramm CA, Zhang Z, Zhu J, Shapiro L, Mullikin JC, Gnanakaran S, Hraber P, Wiehe K, Kelsoe G, Yang G, Xia S-M, Montefiori DC, Parks R, Lloyd KE, Scearce RM, Soderberg KA, Cohen M, Kamanga G, Louder MK, Tran LM, Chen Y, Cai F, Chen S, Moquin S, Du X, Joyce MG, Srivatsan S, Zhang B, Zheng A, Shaw GM, Hahn BH, Kepler TB, Korber BTM, Kwong PD, Mascola JR, Haynes BF, Co-evolution of a broadly neutralizing HIV-1 antibody and founder virus. Nature 496, 469–476 (2013). 23552890 10.1038/nature12053 PMC3637846

[17] Sliepen K, Medina-Ramírez M, Yasmeen A, Moore JP, Klasse PJ, Sanders RW, Binding of inferred germline precursors of broadly neutralizing HIV-1 antibodies to native-like envelope trimers. Virology 486, 116–120 (2015). 26433050 10.1016/j.virol.2015.08.002 PMC4712445

[18] Hoot S, McGuire AT, Cohen KW, Strong RK, Hangartner L, Klein F, Diskin R, Scheid JF, Sather DN, Burton DR, Stamatatos L, Recombinant HIV envelope proteins fail to engage germline versions of anti-CD4bs bNAbs. PLoS Pathog. 9, e1003106 (2013). 23300456 10.1371/journal.ppat.1003106 PMC3536657

[19] Medina-Ramírez M, Garces F, Escolano A, Skog P, de Taeye SW, Del Moral-Sanchez I, McGuire AT, Yasmeen A, Behrens A-J, Ozorowski G, van den Kerkhof TLGM, Freund NT, Dosenovic P, Hua Y, Gitlin AD, Cupo A, van der Woude P, Golabek M, Sliepen K, Blane T, Kootstra N, van Breemen MJ, Pritchard LK, Stanfield RL, Crispin M, Ward AB, Stamatatos L, Klasse PJ, Moore JP, Nemazee D, Nussenzweig MC, Wilson IA, Sanders RW, Design and crystal structure of a native-like HIV-1 envelope trimer that engages multiple broadly neutralizing antibody precursors in vivo. J. Exp. Med. 214, 2573–2590 (2017). 28847869 10.1084/jem.20161160 PMC5584115

[20] Jardine J, Julien J-P, Menis S, Ota T, Kalyuzhniy O, McGuire A, Sok D, Huang P-S, MacPherson S, Jones M, Nieusma T, Mathison J, Baker D, Ward AB, Burton DR, Stamatatos L, Nemazee D, Wilson IA, Schief WR, Rational HIV immunogen design to target specific germline B cell receptors. Science 340, 711–716 (2013). 23539181 10.1126/science.1234150 PMC3689846

[21] Briney B, Sok D, Jardine JG, Kulp DW, Skog P, Menis S, Jacak R, Kalyuzhniy O, de Val N, Sesterhenn F, Le KM, Ramos A, Jones M, Saye-Francisco KL, Blane TR, Spencer S, Georgeson E, Hu X, Ozorowski G, Adachi Y, Kubitz M, Sarkar A, Wilson IA, Ward AB, Nemazee D, Burton DR, Schief WR, Tailored Immunogens Direct Affinity Maturation toward HIV Neutralizing Antibodies. Cell 166, 1459–1470.e1411 (2016). 27610570 10.1016/j.cell.2016.08.005 PMC5018249

[22] Rachael Parks K, MacCamy AJ, Trichka J, Gray M, Weidle C, Borst AJ, Khechaduri A, Takushi B, Agrawal P, Guenaga J, Wyatt RT, Coler R, Seaman M, LaBranche C, Montefiori DC, Veesler D, Pancera M, McGuire A, Stamatatos L, Overcoming Steric Restrictions of VRC01 HIV-1 Neutralizing Antibodies through Immunization. Cell Rep. 29, 3060–3072.e3067 (2019). 31801073 10.1016/j.celrep.2019.10.071 PMC6936959

[23] Borst AJ, Weidle CE, Gray MD, Frenz B, Snijder J, Joyce MG, Georgiev IS, Stewart-Jones GB, Kwong PD, McGuire AT, DiMaio F, Stamatatos L, Pancera M, Veesler D, Germline VRC01 antibody recognition of a modified clade C HIV-1 envelope trimer and a glycosylated HIV-1 gp120 core. Elife 7, (2018). 10.7554/eLife.37688 PMC6237438 30403372

[24] Whitaker N, Hickey JM, Kaur K, Xiong J, Sawant N, Cupo A, Lee W-H, Ozorowski G, Medina-Ramírez M, Ward AB, Sanders RW, Moore JP, Joshi SB, Volkin DB, Dey AK, Developability Assessment of Physicochemical Properties and Stability Profiles of HIV-1 BG505 SOSIP.664 and BG505 SOSIP.v4.1-GT1.1 gp140 Envelope Glycoprotein Trimers as Candidate Vaccine Antigens. J. Pharm. Sci. 108, 2264–2277 (2019). 30776383 10.1016/j.xphs.2019.01.033 PMC6595180

[25] Tian M, Cheng C, Chen X, Duan H, Cheng H-L, Dao M, Sheng Z, Kimble M, Wang L, Lin S, Schmidt SD, Du Z, Joyce MG, Chen Y, DeKosky BJ, Chen Y, Normandin E, Cantor E, Chen RE, Doria-Rose NA, Zhang Y, Shi W, Kong W-P, Choe M, Henry AR, Laboune F, Georgiev IS, Huang P-Y, Jain S, McGuire AT, Georgeson E, Menis S, Douek DC, Schief WR, Stamatatos L, Kwong PD, Shapiro L, Haynes BF, Mascola JR, Alt FW, Induction of HIV Neutralizing Antibody Lineages in Mice with Diverse Precursor Repertoires. Cell 166, 1471–1484.e1418 (2016). 27610571 10.1016/j.cell.2016.07.029 PMC5103708

[26] Jardine JG, Sok D, Julien J-P, Briney B, Sarkar A, Liang C-H, Scherer EA, Henry Dunand CJ, Adachi Y, Diwanji D, Hsueh J, Jones M, Kalyuzhniy O, Kubitz M, Spencer S, Pauthner M, Saye-Francisco KL, Sesterhenn F, Wilson PC, Galloway DM, Stanfield RL, Wilson IA, Burton DR, Schief WR, Minimally Mutated HIV-1 Broadly Neutralizing Antibodies to Guide Reductionist Vaccine Design. PLoS Pathog. 12, e1005815 (2016). 27560183 10.1371/journal.ppat.1005815 PMC4999182

[27] Bale JB, Gonen S, Liu Y, Sheffler W, Ellis D, Thomas C, Cascio D, Yeates TO, Gonen T, King NP, Baker D, Accurate design of megadalton-scale two-component icosahedral protein complexes. Science 353, 389–394 (2016). 27463675 10.1126/science.aaf8818 PMC5485857

[28] Brouwer PJM, Antanasijevic A, Berndsen Z, Yasmeen A, Fiala B, Bijl TPL, Bontjer I, Bale JB, Sheffler W, Allen JD, Schorcht A, Burger JA, Camacho M, Ellis D, Cottrell CA, Behrens A-J, Catalano M, del Moral-Sánchez I, Ketas TJ, LaBranche C, van Gils MJ, Sliepen K, Stewart LJ, Crispin M, Montefiori DC, Baker D, Moore JP, Klasse PJ, Ward AB, King NP, Sanders RW, Enhancing and shaping the immunogenicity of native-like HIV-1 envelope trimers with a two-component protein nanoparticle. Nat. Commun. 10, 1–17 (2019). 31537780 10.1038/s41467-019-12080-1 PMC6753213

[29] Sundling C, Li Y, Huynh N, Poulsen C, Wilson R, O’Dell S, Feng Y, Mascola JR, Wyatt RT, Karlsson Hedestam GB, High-resolution definition of vaccine-elicited B cell responses against the HIV primary receptor binding site. Sci. Transl. Med. 4, 142ra196 (2012). 10.1126/scitranslmed.3003752 PMC5378478 22786681

[30] Navis M, Tran K, Bale S, Phad GE, Guenaga J, Wilson R, Soldemo M, McKee K, Sundling C, Mascola J, Li Y, Wyatt RT, Karlsson Hedestam GB, HIV-1 receptor binding site-directed antibodies using a VH1–2 gene segment orthologue are activated by Env trimer immunization. PLoS Pathog. 10, e1004337 (2014). 25166308 10.1371/journal.ppat.1004337 PMC4148451

[31] Sliepen K, Han BW, Bontjer I, Mooij P, Garces F, Behrens A-J, Rantalainen K, Kumar S, Sarkar A, Brouwer PJM, Hua Y, Tolazzi M, Schermer E, Torres JL, Ozorowski G, van der Woude P, de la Peña AT, van Breemen MJ, Camacho-Sánchez JM, Burger JA, Medina-Ramírez M, González N, Alcami J, LaBranche C, Scarlatti G, van Gils MJ, Crispin M, Montefiori DC, Ward AB, Koopman G, Moore JP, Shattock RJ, Bogers WM, Wilson IA, Sanders RW, Structure and immunogenicity of a stabilized HIV-1 envelope trimer based on a group-M consensus sequence. Nat. Commun. 10, 1–16 (2019). 31142746 10.1038/s41467-019-10262-5 PMC6541627

[32] Baldwin SL, Roeffen W, Singh SK, Tiendrebeogo RW, Christiansen M, Beebe E, Carter D, Fox CB, Howard RF, Reed SG, Sauerwein R, Theisen M, Synthetic TLR4 agonists enhance functional antibodies and CD4+ T-cell responses against the Plasmodium falciparum GMZ2.6C multi-stage vaccine antigen. Vaccine 34, 2207–2215 (2016). 26994314 10.1016/j.vaccine.2016.03.016

[33] Saunders KO, Edwards RJ, Tilahun K, Manne K, Lu X, Cain DW, Wiehe K, Williams WB, Mansouri K, Hernandez GE, Sutherland L, Scearce R, Parks R, Barr M, DeMarco T, Eater CM, Eaton A, Morton G, Mildenberg B, Wang Y, Rountree RW, Tomai MA, Fox CB, Moody MA, Alam SM, Santra S, Lewis MG, Denny TN, Shaw GM, Montefiori DC, Acharya P, Haynes BF, Stabilized HIV-1 envelope immunization induces neutralizing antibodies to the CD4bs and protects macaques against mucosal infection. Sci. Transl. Med. 14, eabo5598 (2022). 36070369 10.1126/scitranslmed.abo5598 PMC10034035

[34] Kasturi SP, Rasheed MAU, Havenar-Daughton C, Pham M, Legere T, Sher ZJ, Kovalenkov Y, Gumber S, Huang JY, Gottardo R, Fulp W, Sato A, Sawant S, Stanfield-Oakley S, Yates N, LaBranche C, Alam SM, Tomaras G, Ferrari G, Montefiori D, Wrammert J, Villinger F, Tomai M, Vasilakos J, Fox CB, Reed SG, Haynes BF, Crotty S, Ahmed R, Pulendran B, 3M-052, a synthetic TLR-7/8 agonist, induces durable HIV-1 envelope-specific plasma cells and humoral immunity in nonhuman primates. Sci Immunol 5, (2020). 10.1126/sciimmunol.abb1025 PMC8109745 32561559

[35] Sliepen K, Schermer E, Bontjer I, Burger JA, Lévai RF, Mundsperger P, Brouwer PJM, Tolazzi M, Farsang A, Katinger D, Moore JP, Scarlatti G, Shattock RJ, Sattentau QJ, Sanders RW, Interplay of diverse adjuvants and nanoparticle presentation of native-like HIV-1 envelope trimers. NPJ Vaccines 6, 103 (2021). 34404812 10.1038/s41541-021-00364-x PMC8371121

[36] LaBranche CC, Henderson R, Hsu A, Behrens S, Chen X, Zhou T, Wiehe K, Saunders KO, Alam SM, Bonsignori M, Borgnia MJ, Sattentau QJ, Eaton A, Greene K, Gao H, Liao H-X, Williams WB, Peacock J, Tang H, Perez LG, Edwards RJ, Kepler TB, Korber BT, Kwong PD, Mascola JR, Acharya P, Haynes BF, Montefiori DC, Neutralization-guided design of HIV-1 envelope trimers with high affinity for the unmutated common ancestor of CH235 lineage CD4bs broadly neutralizing antibodies. PLoS Pathog. 15, e1008026 (2019). 31527908 10.1371/journal.ppat.1008026 PMC6764681

[37] Nogal B, Bianchi M, Cottrell CA, Kirchdoerfer RN, Sewall LM, Turner HL, Zhao F, Sok D, Burton DR, Hangartner L, Ward AB, Mapping Polyclonal Antibody Responses in Non-human Primates Vaccinated with HIV Env Trimer Subunit Vaccines. Cell Rep. 30, 3755–3765.e3757 (2020). 32187547 10.1016/j.celrep.2020.02.061 PMC7153566

[38] Antanasijevic A, Sewall LM, Cottrell CA, Carnathan DG, Jimenez LE, Ngo JT, Silverman JB, Groschel B, Georgeson E, Bhiman J, Bastidas R, LaBranche C, Allen JD, Copps J, Perrett HR, Rantalainen K, Cannac F, Yang YR, de la Peña AT, Rocha RF, Berndsen ZT, Baker D, King NP, Sanders RW, Moore JP, Crotty S, Crispin M, Montefiori DC, Burton DR, Schief WR, Silvestri G, Ward AB, Polyclonal antibody responses to HIV Env immunogens resolved using cryoEM. Nat. Commun. 12, 1–17 (2021). 34376662 10.1038/s41467-021-25087-4 PMC8355326

[39] Sanders RW, van Gils MJ, Derking R, Sok D, Ketas TJ, Burger JA, Ozorowski G, Cupo A, Simonich C, Goo L, Arendt H, Kim HJ, Lee JH, Pugach P, Williams M, Debnath G, Moldt B, van Breemen MJ, Isik G, Medina-Ramírez M, Back JW, Koff WC, Julien J-P, Rakasz EG, Seaman MS, Guttman M, Lee KK, Klasse PJ, LaBranche C, Schief WR, Wilson IA, Overbaugh J, Burton DR, Ward AB, Montefiori DC, Dean H, Moore JP, HIV-1 VACCINES. HIV-1 neutralizing antibodies induced by native-like envelope trimers. Science 349, aac4223 (2015). 26089353 10.1126/science.aac4223 PMC4498988

[40] McCoy LE, van Gils MJ, Ozorowski G, Messmer T, Briney B, Voss JE, Kulp DW, Macauley MS, Sok D, Pauthner M, Menis S, Cottrell CA, Torres JL, Hsueh J, Schief WR, Wilson IA, Ward AB, Sanders RW, Burton DR, Holes in the Glycan Shield of the Native HIV Envelope Are a Target of Trimer-Elicited Neutralizing Antibodies. Cell Rep. 16, 2327–2338 (2016). 27545891 10.1016/j.celrep.2016.07.074 PMC5007210

[41] Dingens AS, Pratap P, Malone K, Hilton SK, Ketas T, Cottrell CA, Overbaugh J, Moore JP, Klasse PJ, Ward AB, Bloom JD, High-resolution mapping of the neutralizing and binding specificities of polyclonal sera post-HIV Env trimer vaccination. Elife 10, (2021). 10.7554/eLife.64281 PMC7864656 33438580

[42] Zhao F, Joyce C, Burns A, Nogal B, Cottrell CA, Ramos A, Biddle T, Pauthner M, Nedellec R, Qureshi H, Mason R, Landais E, Briney B, Ward AB, Burton DR, Sok D, Mapping Neutralizing Antibody Epitope Specificities to an HIV Env Trimer in Immunized and in Infected Rhesus Macaques. Cell Rep. 32, 108122 (2020). 32905766 10.1016/j.celrep.2020.108122 PMC7487785

[43] Bianchi M, Turner HL, Nogal B, Cottrell CA, Oyen D, Pauthner M, Bastidas R, Nedellec R, McCoy LE, Wilson IA, Burton DR, Ward AB, Hangartner L, Electron-Microscopy-Based Epitope Mapping Defines Specificities of Polyclonal Antibodies Elicited during HIV-1 BG505 Envelope Trimer Immunization. Immunity 49, 288–300.e288 (2018). 30097292 10.1016/j.immuni.2018.07.009 PMC6104742

[44] Houser KV, Gaudinski MR, Happe M, Narpala S, Verardi R, Sarfo EK, Corrigan AR, Wu R, Rothwell RS, Novik L, Hendel CS, Gordon IJ, Berkowitz NM, Cartagena CT, Widge AT, Coates EE, Strom L, Hickman S, Conan-Cibotti M, Vazquez S, Trofymenko O, Plummer S, Stein J, Case CL, Nason M, Biju A, Parchment DK, Changela A, Cheng C, Duan H, Geng H, Teng IT, Zhou T, O’Connell S, Barry C, Carlton K, Gall JG, Flach B, Doria-Rose NA, Graham BS, Koup RA, McDermott AB, Mascola JR, Kwong PD, Ledgerwood JE, Team VRCS, Safety and immunogenicity of an HIV-1 prefusion-stabilized envelope trimer (Trimer 4571) vaccine in healthy adults: A first-in-human open-label, randomized, dose-escalation, phase 1 clinical trial. EClinicalMedicine 48, 101477 (2022). 35783486 10.1016/j.eclinm.2022.101477 PMC9249552

[45] deCamp A, Hraber P, Bailer RT, Seaman MS, Ochsenbauer C, Kappes J, Gottardo R, Edlefsen P, Self S, Tang H, Greene K, Gao H, Daniell X, Sarzotti-Kelsoe M, Gorny MK, Zolla-Pazner S, LaBranche CC, Mascola JR, Korber BT, Montefiori DC, Global panel of HIV-1 Env reference strains for standardized assessments of vaccine-elicited neutralizing antibodies. J. Virol. 88, 2489–2507 (2014). 24352443 10.1128/JVI.02853-13 PMC3958090

[46] Berndsen ZT, Chakraborty S, Wang X, Cottrell CA, Torres JL, Diedrich JK, López CA, Yates JR 3rd, van Gils MJ, Paulson JC, Gnanakaran S, Ward AB, Visualization of the HIV-1 Env glycan shield across scales. Proc. Natl. Acad. Sci. U. S. A. 117, 28014–28025 (2020). 33093196 10.1073/pnas.2000260117 PMC7668054

[47] Cottrell CA, van Schooten J, Bowman CA, M., Oyen D, Shin M, Morpurgo R, van der Woude P, van Breemen M, Torres JL, Patel R, Gross J, Sewall LM, Copps J, Ozorowski G, Nogal B, Sok D, Rakasz EG, Labranche C, Vigdorovich V, Christley S, Carnathan DG, Sather DN, Montefiori D, Silvestri G, Burton DR, Moore JP, Wilson IA, Sanders RW, Ward AB, van Gils MJ, Mapping the immunogenic landscape of near-native HIV-1 envelope trimers in non-human primates. PLoS Pathog. 16, e1008753 (2020). 32866207 10.1371/journal.ppat.1008753 PMC7485981

[48] Yasmeen A, Ringe R, Derking R, Cupo A, Julien J-P, Burton DR, Ward AB, Wilson IA, Sanders RW, Moore JP, Klasse PJ, Differential binding of neutralizing and non-neutralizing antibodies to native-like soluble HIV-1 Env trimers, uncleaved Env proteins, and monomeric subunits. Retrovirology 11, 41 (2014). 24884783 10.1186/1742-4690-11-41 PMC4067080

[49] Scharf L, West AP Jr., Gao H, Lee T, Scheid JF, Nussenzweig MC, Bjorkman PJ, Diskin R, Structural basis for HIV-1 gp120 recognition by a germ-line version of a broadly neutralizing antibody. Proc. Natl. Acad. Sci. U. S. A. 110, 6049–6054 (2013). 23524883 10.1073/pnas.1303682110 PMC3625305

[50] Kepler TB, Liao H-X, Alam SM, Bhaskarabhatla R, Zhang R, Yandava C, Stewart S, Anasti K, Kelsoe G, Parks R, Lloyd KE, Stolarchuk C, Pritchett J, Solomon E, Friberg E, Morris L, Karim SSA, Cohen MS, Walter E, Moody MA, Wu X, Altae-Tran HR, Georgiev IS, Kwong PD, Boyd SD, Fire AZ, Mascola JR, Haynes BF, Immunoglobulin gene insertions and deletions in the affinity maturation of HIV-1 broadly reactive neutralizing antibodies. Cell Host Microbe 16, 304–313 (2014). 25211073 10.1016/j.chom.2014.08.006 PMC4163498

[51] Plotkin SA, Vaccines: correlates of vaccine-induced immunity. Clin. Infect. Dis. 47, 401–409 (2008). 18558875 10.1086/589862

[52] Plotkin SA, Correlates of protection induced by vaccination. Clin. Vaccine Immunol. 17, 1055–1065 (2010). 20463105 10.1128/CVI.00131-10 PMC2897268

[53] Jardine J, Kulp DW, Havenar-Daughton C, Sarkar A, Briney B, Sok D, Sesterhenn F, Ereño-Orbea J, Kalyuzhniy O, Deresa I, Hu X, Spencer S, Jones M, Georgeson E, Adachi Y, Kubitz M, deCamp AC, Julien J-P, Wilson IA, Burton DR, Crotty S, Schief WR, HIV-1 broadly neutralizing antibody precursor B cells revealed by germline-targeting immunogen. Science 351, 1458–1463 (2016). 27013733 10.1126/science.aad9195 PMC4872700

[54] Sanders RW, Derking R, Cupo A, Julien J-P, Yasmeen A, de Val N, Kim HJ, Blattner C, de la Peña AT, Korzun J, Golabek M, de Los Reyes K, Ketas TJ, van Gils MJ, King CR, Wilson IA, Ward AB, Klasse PJ, Moore JP, A next-generation cleaved, soluble HIV-1 Env trimer, BG505 SOSIP.664 gp140, expresses multiple epitopes for broadly neutralizing but not non-neutralizing antibodies. PLoS Pathog. 9, e1003618 (2013). 24068931 10.1371/journal.ppat.1003618 PMC3777863

[55] de Taeye SW, Ozorowski G, Torrents de la Peña A, Guttman M, Julien J-P, van den Kerkhof TLGM, Burger JA, Pritchard LK, Pugach P, Yasmeen A, Crampton J, Hu J, Bontjer I, Torres JL, Arendt H, DeStefano J, Koff WC, Schuitemaker H, Eggink D, Berkhout B, Dean H, LaBranche C, Crotty S, Crispin M, Montefiori DC, Klasse PJ, Lee KK, Moore JP, Wilson IA, Ward AB, Sanders RW, Immunogenicity of Stabilized HIV-1 Envelope Trimers with Reduced Exposure of Non-neutralizing Epitopes. Cell 163, 1702–1715 (2015). 26687358 10.1016/j.cell.2015.11.056 PMC4732737

[56] Karlsson R, Ståhlberg R, Surface plasmon resonance detection and multispot sensing for direct monitoring of interactions involving low-molecular-weight analytes and for determination of low affinities. Anal. Biochem. 228, 274–280 (1995). 8572306 10.1006/abio.1995.1350

[57] Brouwer PJM, Brinkkemper M, Maisonnasse P, Dereuddre-Bosquet N, Grobben M, Claireaux M, de Gast M, Marlin R, Chesnais V, Diry S, Allen JD, Watanabe Y, Giezen JM, Kerster G, Turner HL, van der Straten K, van der Linden CA, Aldon Y, Naninck T, Bontjer I, Burger JA, Poniman M, Mykytyn AZ, Okba NMA, Schermer EE, van Breemen MJ, Ravichandran R, Caniels TG, van Schooten J, Kahlaoui N, Contreras V, Lemaître J, Chapon C, Fang RHT, Villaudy J, Sliepen K, van der Velden YU, Haagmans BL, de Bree GJ, Ginoux E, Ward AB, Crispin M, King NP, van der Werf S, van Gils MJ, Le Grand R, Sanders RW, Two-component spike nanoparticle vaccine protects macaques from SARS-CoV-2 infection. Cell 184, 1188–1200.e1119 (2021). 33577765 10.1016/j.cell.2021.01.035 PMC7834972

[58] Claireaux M, Caniels TG, de Gast M, Han J, Guerra D, Kerster G, van Schaik BDC, Jongejan A, Schriek AI, Grobben M, Brouwer PJM, van der Straten K, Aldon Y, Capella-Pujol J, Snitselaar JL, Olijhoek W, Aartse A, Brinkkemper M, Bontjer I, Burger JA, Poniman M, Bijl TPL, Torres JL, Copps J, Martin IC, de Taeye SW, de Bree GJ, Ward AB, Sliepen K, van Kampen AHC, Moerland PD, Sanders RW, van Gils MJ, A public antibody class recognizes an S2 epitope exposed on open conformations of SARS-CoV-2 spike. Nat. Commun. 13, 4539 (2022). 35927266 10.1038/s41467-022-32232-0 PMC9352689

[59] von Boehmer L, Liu C, Ackerman S, Gitlin AD, Wang Q, Gazumyan A, Nussenzweig MC, Sequencing and cloning of antigen-specific antibodies from mouse memory B cells. Nat. Protoc. 11, 1908–1923 (2016). 27658009 10.1038/nprot.2016.102

[60] Gupta NT, Vander Heiden JA, Uduman M, Gadala-Maria D, Yaari G, Kleinstein SH, Change-O: a toolkit for analyzing large-scale B cell immunoglobulin repertoire sequencing data. Bioinformatics 31, 3356–3358 (2015). 26069265 10.1093/bioinformatics/btv359 PMC4793929

[61] Brouwer PJM, Caniels TG, van der Straten K, Snitselaar JL, Aldon Y, Bangaru S, Torres JL, Okba NMA, Claireaux M, Kerster G, Bentlage AEH, van Haaren MM, Guerra D, Burger JA, Schermer EE, Verheul KD, van der Velde N, van der Kooi A, van Schooten J, van Breemen MJ, Bijl TPL, Sliepen K, Aartse A, Derking R, Bontjer I, Kootstra NA, Wiersinga WJ, Vidarsson G, Haagmans BL, Ward AB, de Bree GJ, Sanders RW, van Gils MJ, Potent neutralizing antibodies from COVID-19 patients define multiple targets of vulnerability. Science 369, 643–650 (2020). 32540902 10.1126/science.abc5902 PMC7299281

[62] Caniels TG, Bontjer I, van der Straten K, Poniman M, Burger JA, Appelman B, Lavell AHA, Oomen M, Godeke G-J, Valle C, Mögling R, van Willigen HDG, Wynberg E, Schinkel M, van Vught LA, Guerra D, Snitselaar JL, Chaturbhuj DN, Martin IC, Amsterdam U. M. C. C.-S. H. C. W. s. g., Moore JP, de Jong MD, Reusken C, Sikkens JJ, Bomers MK, de Bree GJ, van Gils MJ, Eggink D, Sanders RW, Emerging SARS-CoV-2 variants of concern evade humoral immune responses from infection and vaccination. medRxiv, (2021). 10.1126/sciadv.abj5365 PMC8442901 34516917

[63] Cupo A, Cruz Portillo VM, Gelfand P, Yasmeen A, Klasse PJ, Moore JP, Optimizing the production and affinity purification of HIV-1 envelope glycoprotein SOSIP trimers from transiently transfected CHO cells. PLoS One 14, e0215106 (2019). 30958859 10.1371/journal.pone.0215106 PMC6453562

[64] Montefiori DC, Measuring HIV neutralization in a luciferase reporter gene assay. Methods Mol. Biol. 485, 395–405 (2009). 19020839 10.1007/978-1-59745-170-3_26

[65] Li M, Gao F, Mascola JR, Stamatatos L, Polonis VR, Koutsoukos M, Voss G, Goepfert P, Gilbert P, Greene KM, Bilska M, Kothe DL, Salazar-Gonzalez JF, Wei X, Decker JM, Hahn BH, Montefiori DC, Human immunodeficiency virus type 1 env clones from acute and early subtype B infections for standardized assessments of vaccine-elicited neutralizing antibodies. J. Virol. 79, 10108–10125 (2005). 16051804 10.1128/JVI.79.16.10108-10125.2005 PMC1182643

[66] Platt EJ, Wehrly K, Kuhmann SE, Chesebro B, Kabat D, Effects of CCR5 and CD4 cell surface concentrations on infections by macrophagetropic isolates of human immunodeficiency virus type 1. J. Virol. 72, 2855–2864 (1998). 9525605 10.1128/jvi.72.4.2855-2864.1998 PMC109730

[67] Wei X, Decker JM, Liu H, Zhang Z, Arani RB, Kilby JM, Saag MS, Wu X, Shaw GM, Kappes JC, Emergence of resistant human immunodeficiency virus type 1 in patients receiving fusion inhibitor (T-20) monotherapy. Antimicrob. Agents Chemother. 46, 1896–1905 (2002). 12019106 10.1128/AAC.46.6.1896-1905.2002 PMC127242

[68] Sarzotti-Kelsoe M, Bailer RT, Turk E, Lin C-L, Bilska M, Greene KM, Gao H, Todd CA, Ozaki DA, Seaman MS, Mascola JR, Montefiori DC, Optimization and validation of the TZM-bl assay for standardized assessments of neutralizing antibodies against HIV-1. J. Immunol. Methods 409, 131–146 (2014). 24291345 10.1016/j.jim.2013.11.022 PMC4040342

[69] Todd CA, Greene KM, Yu X, Ozaki DA, Gao H, Huang Y, Wang M, Li G, Brown R, Wood B, D’Souza MP, Gilbert P, Montefiori DC, Sarzotti-Kelsoe M, Development and implementation of an international proficiency testing program for a neutralizing antibody assay for HIV-1 in TZM-bl cells. J. Immunol. Methods 375, 57–67 (2012). 21968254 10.1016/j.jim.2011.09.007 PMC3332116

[70] Lee JH, Sutton HJ, Cottrell CA, Phung I, Ozorowski G, Sewall LM, Nedellec R, Nakao C, Silva M, Richey ST, Torres JL, Lee W-H, Georgeson E, Kubitz M, Hodges S, Mullen T-M, Adachi Y, Cirelli KM, Kaur A, Allers C, Fahlberg M, Grasperge BF, Dufour JP, Schiro F, Aye PP, Kalyuzhniy O, Liguori A, Carnathan DG, Silvestri G, Shen X, Montefiori DC, Veazey RS, Ward AB, Hangartner L, Burton DR, Irvine DJ, Schief WR, Crotty S, Long-primed germinal centres with enduring affinity maturation and clonal migration. Nature 609, 998–1004 (2022). 36131022 10.1038/s41586-022-05216-9 PMC9491273

[71] Zivanov J, Nakane T, Forsberg BO, Kimanius D, Hagen WJH, Lindahl E, Scheres SHW, New tools for automated high-resolution cryo-EM structure determination in RELION-3. Elife 7, e42166 (2018). 30412051 10.7554/eLife.42166 PMC6250425

[72] Pettersen EF, Goddard TD, Huang CC, Couch GS, Greenblatt DM, Meng EC, Ferrin TE, UCSF Chimera--a visualization system for exploratory research and analysis. J. Comput. Chem. 25, 1605–1612 (2004). 15264254 10.1002/jcc.20084

[73] Roark RS, Li H, Williams WB, Chug H, Mason RD, Gorman J, Wang S, Lee F-H, Rando J, Bonsignori M, Hwang K-K, Saunders KO, Wiehe K, Moody MA, Hraber PT, Wagh K, Giorgi EE, Russell RM, Bibollet-Ruche F, Liu W, Connell J, Smith AG, DeVoto J, Murphy AI, Smith J, Ding W, Zhao C, Chohan N, Okumura M, Rosario C, Ding Y, Lindemuth E, Bauer AM, Bar KJ, Ambrozak D, Chao CW, Chuang G-Y, Geng H, Lin BC, Louder MK, Nguyen R, Zhang B, Lewis MG, Raymond DD, Doria-Rose NA, Schramm CA, Douek DC, Roederer M, Kepler TB, Kelsoe G, Mascola JR, Kwong PD, Korber BT, Harrison SC, Haynes BF, Hahn BH, Shaw GM, Recapitulation of HIV-1 Env-antibody coevolution in macaques leading to neutralization breadth. Science 371, (2021). 10.1126/science.abd2638 PMC8040783 33214287

[74] Huang J, Doria-Rose NA, Longo NS, Laub L, Lin C-L, Turk E, Kang BH, Migueles SA, Bailer RT, Mascola JR, Connors M, Isolation of human monoclonal antibodies from peripheral blood B cells. Nat. Protoc. 8, 1907–1915 (2013). 24030440 10.1038/nprot.2013.117 PMC4844175

[75] Doria-Rose NA, Schramm CA, Gorman J, Moore PL, Bhiman JN, DeKosky BJ, Ernandes MJ, Georgiev IS, Kim HJ, Pancera M, Staupe RP, Altae-Tran HR, Bailer RT, Crooks ET, Cupo A, Druz A, Garrett NJ, Hoi KH, Kong R, Louder MK, Longo NS, McKee K, Nonyane M, O’Dell S, Roark RS, Rudicell RS, Schmidt SD, Sheward DJ, Soto C, Wibmer CK, Yang Y, Zhang Z, Program NCS, Mullikin JC, Binley JM, Sanders RW, Wilson IA, Moore JP, Ward AB, Georgiou G, Williamson C, Abdool Karim SS, Morris L, Kwong PD, Shapiro L, Mascola JR, Developmental pathway for potent V1V2-directed HIV-neutralizing antibodies. Nature 509, 55–62 (2014). 24590074 10.1038/nature13036 PMC4395007

[76] Tiller T, Meffre E, Yurasov S, Tsuiji M, Nussenzweig MC, Wardemann H, Efficient generation of monoclonal antibodies from single human B cells by single cell RT-PCR and expression vector cloning. J. Immunol. Methods 329, 112–124 (2008). 17996249 10.1016/j.jim.2007.09.017 PMC2243222

[77] Ramesh A, Darko S, Hua A, Overman G, Ransier A, Francica JR, Trama A, Tomaras GD, Haynes BF, Douek DC, Kepler TB, Structure and Diversity of the Rhesus Macaque Immunoglobulin Loci through Multiple De Novo Genome Assemblies. Front. Immunol. 8, 1407 (2017). 29163486 10.3389/fimmu.2017.01407 PMC5663730

[78] Vázquez Bernat N, Corcoran M, Nowak I, Kaduk M, Castro Dopico X, Narang S, Maisonasse P, Dereuddre-Bosquet N, Murrell B, Karlsson Hedestam GB, Rhesus and cynomolgus macaque immunoglobulin heavy-chain genotyping yields comprehensive databases of germline VDJ alleles. Immunity 54, 355–366.e354 (2021). 33484642 10.1016/j.immuni.2020.12.018

[79] Punjani A, Rubinstein JL, Fleet DJ, Brubaker MA, cryoSPARC: algorithms for rapid unsupervised cryo-EM structure determination. Nat. Methods 14, 290–296 (2017). 28165473 10.1038/nmeth.4169

[80] Casañal A, Lohkamp B, Emsley P, Current developments in Coot for macromolecular model building of Electron Cryo-microscopy and Crystallographic Data. Protein Sci. 29, 1069–1078 (2020). 31730249 10.1002/pro.3791 PMC7096722

[81] Conway P, Tyka MD, DiMaio F, Konerding DE, Baker D, Relaxation of backbone bond geometry improves protein energy landscape modeling. Protein Sci. 23, 47–55 (2014). 24265211 10.1002/pro.2389 PMC3892298

[82] Afonine PV, Poon BK, Read RJ, Sobolev OV, Terwilliger TC, Urzhumtsev A, Adams PD, Real-space refinement in PHENIX for cryo-EM and crystallography. Acta Crystallogr D Struct Biol 74, 531–544 (2018). 29872004 10.1107/S2059798318006551 PMC6096492

[83] Otwinowski Z, Minor W, Processing of X-ray diffraction data collected in oscillation mode. Methods Enzymol. 276, 307–326 (1997). 27754618 10.1016/S0076-6879(97)76066-X

[84] McCoy AJ, Grosse-Kunstleve RW, Adams PD, Winn MD, Storoni LC, Read RJ, Phaser crystallographic software. J. Appl. Crystallogr. 40, 658–674 (2007). 19461840 10.1107/S0021889807021206 PMC2483472

[85] Emsley P, Lohkamp B, Scott WG, Cowtan K, Features and development of Coot. Acta Crystallogr. D Biol. Crystallogr. 66, 486–501 (2010). 20383002 10.1107/S0907444910007493 PMC2852313

[86] Adams PD, Afonine PV, Bunkóczi G, Chen VB, Davis IW, Echols N, Headd JJ, Hung L-W, Kapral GJ, Grosse-Kunstleve RW, McCoy AJ, Moriarty NW, Oeffner R, Read RJ, Richardson DC, Richardson JS, Terwilliger TC, Zwart PH, PHENIX: a comprehensive Python-based system for macromolecular structure solution. Acta Crystallogr. D Biol. Crystallogr. 66, 213–221 (2010). 20124702 10.1107/S0907444909052925 PMC2815670

