# Supplementary material for: Germline-targeting HIV vaccination induces neutralizing antibodies to the CD4 binding site
Source: Sci Immunol. Author manuscript; Available in PMC 2025 Jan 31. (PMC11783328; doi:10.1126/sciimmunol.adk9550)
Supplement: Supplementary Figures and Tables [file NIHMS2025519-supplement-Supplementary_Figures_and_Tables.docx]

**Figures S1-S9**

**Tables S1-S5**

**
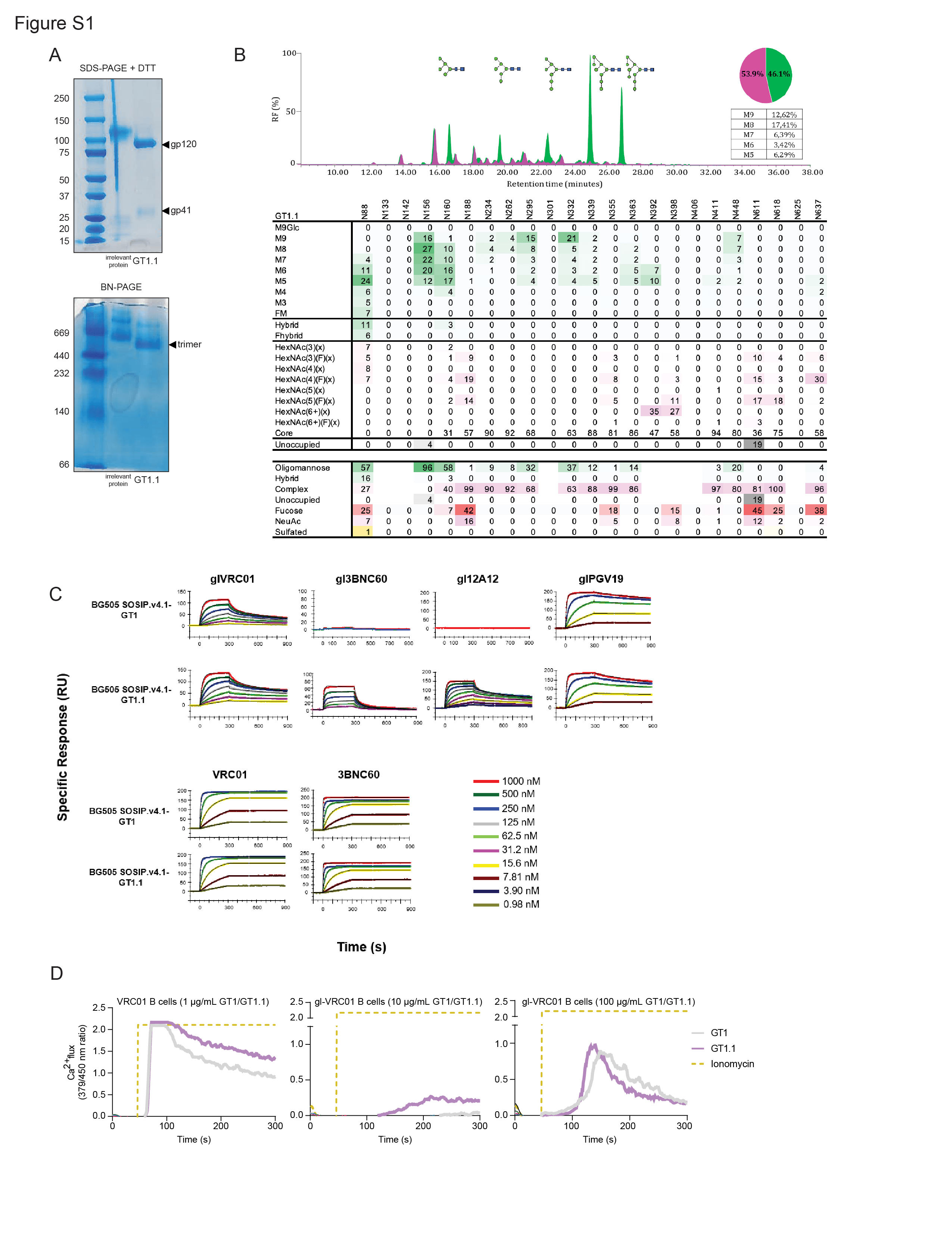
**

**Figure S1. Additional biochemical validation of GT1.1. Related to Figure 1.** (A) Sodium-dodecyl sulfate-polyacrylamide gel electrophoresis (SDS-PAGE) in reducing conditions (+DTT, left panel) and a blue-native PAGE of GT1.1. (B) HILIC-UPLC analysis of GT1.1. Peaks colored green represent endoH-cleavable glycans (oligomannose/hybrid). The spectrum shows the overall oligomannose (green) and complex/hybrid (magenta) content, as represented by a pie chart. (C) SPR analysis of MAbs binding to GT1.1. The sensorgrams show the specific binding signal in response units (RU) on the y axes as a function of time during association (300 s) and dissociation (600 s) on the x axes. The NAb concentrations for the empirical binding curves are given in the color-coded legend; the bivalent-model-fitted curves are black. Note that some scales on the y axes differ. (D) Calcium flux assay as a measure for B cell activation for Ramos B cell lines expressing two distinct B cell receptors: VRC01, left panel; gl-VRC01, middle and right panel. The concentration of trimer that was used is indicated on top of the panels.

**
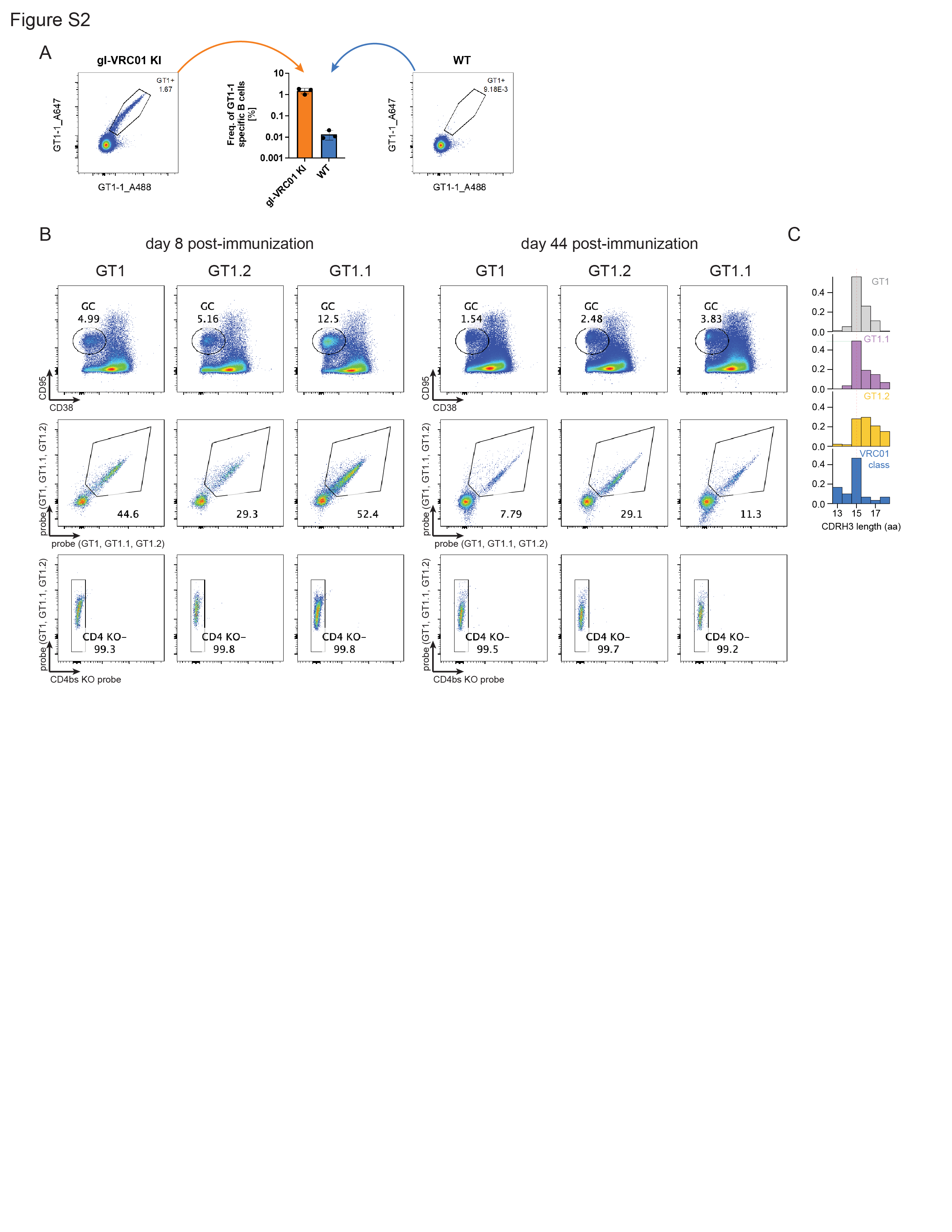
**

**Figure S2. Validation of a VRC01-class precursor knock-in mouse model. Related to Figure 2.** (A) Frequency of GT1.1-specific naive B cells in the knock-in mouse model (left panel) versus a WT C57/BL6 mouse. The bottom panel represents the mean frequency of GT1.1-specific B cells for three individual mice. (B) Example gating scheme for GT1.1-specific germinal center B cells, used for the sort in Fig. 2. (C) The distribution of CDRH3 lengths (aa) for B cells isolated at day 44 from each of the immunization groups, as well as VRC01-class bNAbs for comparison.

**
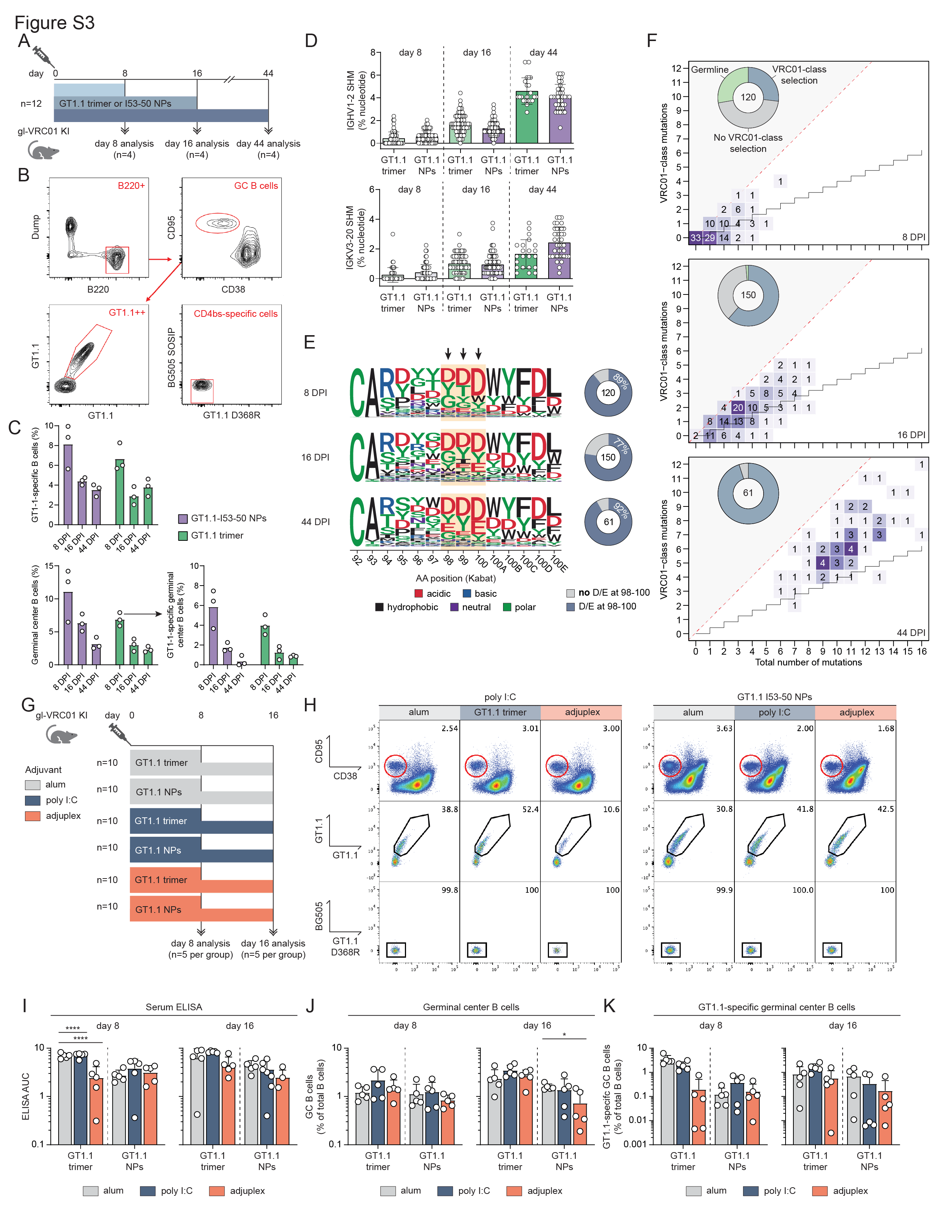
**

**Figure S3. Additional knock-in mouse experiments with GT1.1 and GT1.1 nanoparticles in different formulations.** (A) Schematic of the immunization schedule. Mice were immunized once at day 0 and sacrificed at day 8, 16 or 44 (n = 4 per time point) as indicated by the colored bars. (B) Example gating scheme for GT1.1-specific germinal center B cells, used for the sort in Fig. S3D-F and Fig. 2F-H. (C) The frequency of GT1.1-specific splenic B cells (top panel), germinal center B cells (bottom left panel) and GT1.1-specific germinal center B cells at days 8, 16 and 44 post-immunization for both immunization regimens. (C) The frequency of GT1.1-specific splenic B cells (top panel), germinal center B cells (bottom left panel) and GT1.1-specific germinal center B cells at days 8, 16 and 44 post-immunization for both immunization regimens. (D) Nucleotide somatic hypermutation (SHM) in GT1.1-specific B cells from the experiment in Fig. 2 for IGHV1-2 segments (left panel) and IGKV3-20 segments (right panel). Each dot represents one B cell. (E) Logo plot showing the CDRH3 sequence of knock-in BCRs isolated from each of the groups indicated. The donut plots on the right summarize the percentage of isolated knock-in BCRs with a negative charge at CDRH3 positions 98-100. (F) Total and VRC01-class amino acid mutations in the IGHV1-2 region for recovered knock-in BCR sequences for each time point. The staggered black line shows the expected level of VRC01-class mutations as expected to be introduced by random SHM in IGHV1-2^21^. (G) Schematic overview of the immunization scheme used to test different adjuvants (grey: alum, blue: poly I:C, red: Adjuplex). (H) Example gating scheme for the experiment as in (G). (I) Area under the curve (AUC) as a measure for serum antibody binding to GT1.1 for each of the immunization groups (x-axis) and immunization time points. ****, *p* < 0.001; unpaired, nonparametric Mann-Whitney test. (J) Frequency of germinal center (GC) B cells for each of the immunization groups (x-axis) and immunization time points. *, *p* < 0.05; unpaired, nonparametric Mann-Whitney test. (K) Frequency of GT1.1-specific germinal center (GC) B cells for each of the immunization groups (x-axis) and immunization time points.

**
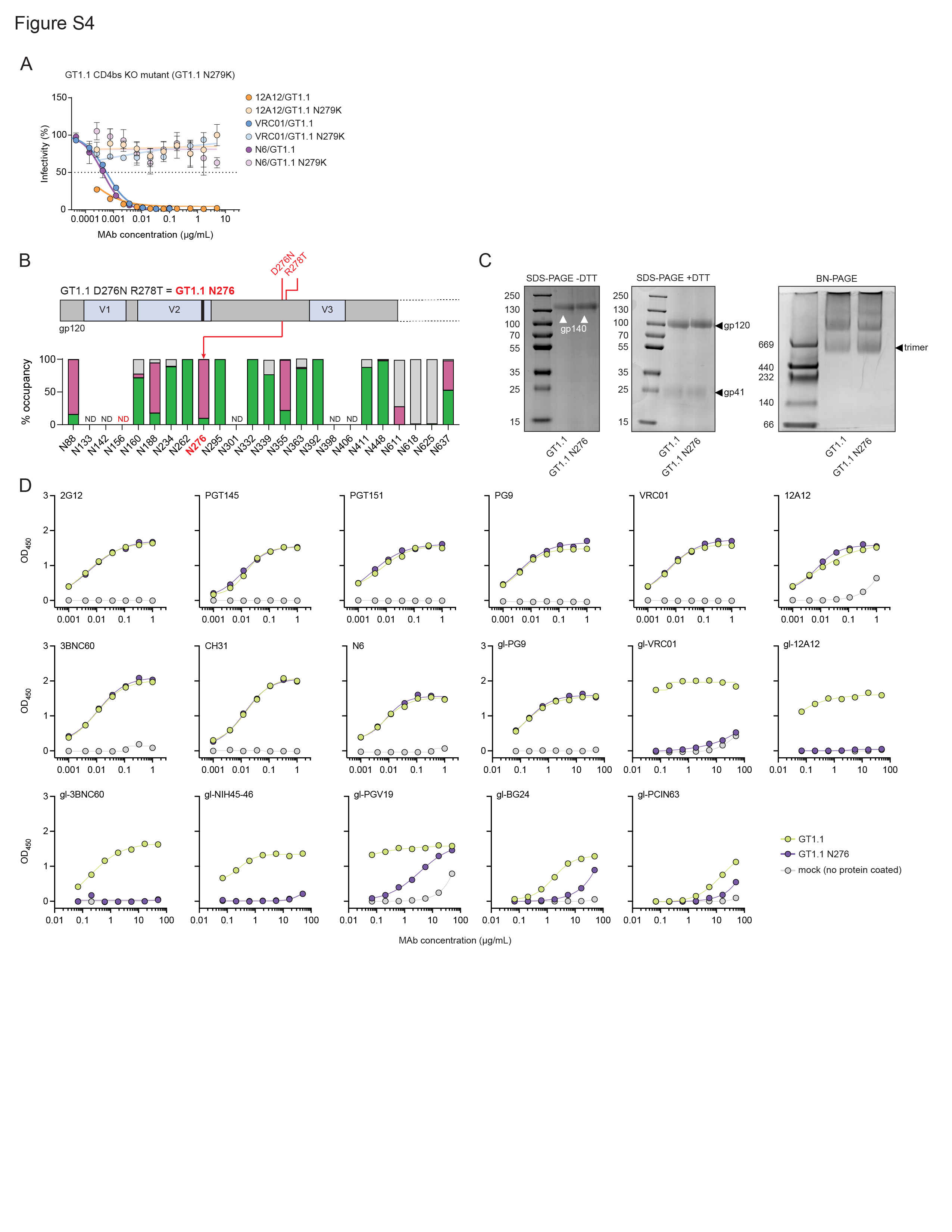
**

**Figure S4. Characterization of GT1.1 N276 trimers and GT1.1 and GT1.2-based pseudoviruses. Related to Figures 2-4.** (A) Neutralization of GT1.1 and GT1.1 N279K pseudoviruses. Neutralization of three known VRC01-class bNAbs is knocked out to the GT1.1 N279K (CD4bs KO) pseudovirus. (B) Schematic linear representation of GT1.1 N276 with glycan occupancy data below. The two amino acid mutations compared to GT1.1 are indicated in red, leading to occupancy of the N276 potential N-linked glycosylation site. (C) SDS-PAGE and BN-PAGE analysis of GT1.1 and GT1.1 N276. GT1.1 N276 forms well-ordered trimers. (D) ELISA antigenicity of GT1.1 N276.

**
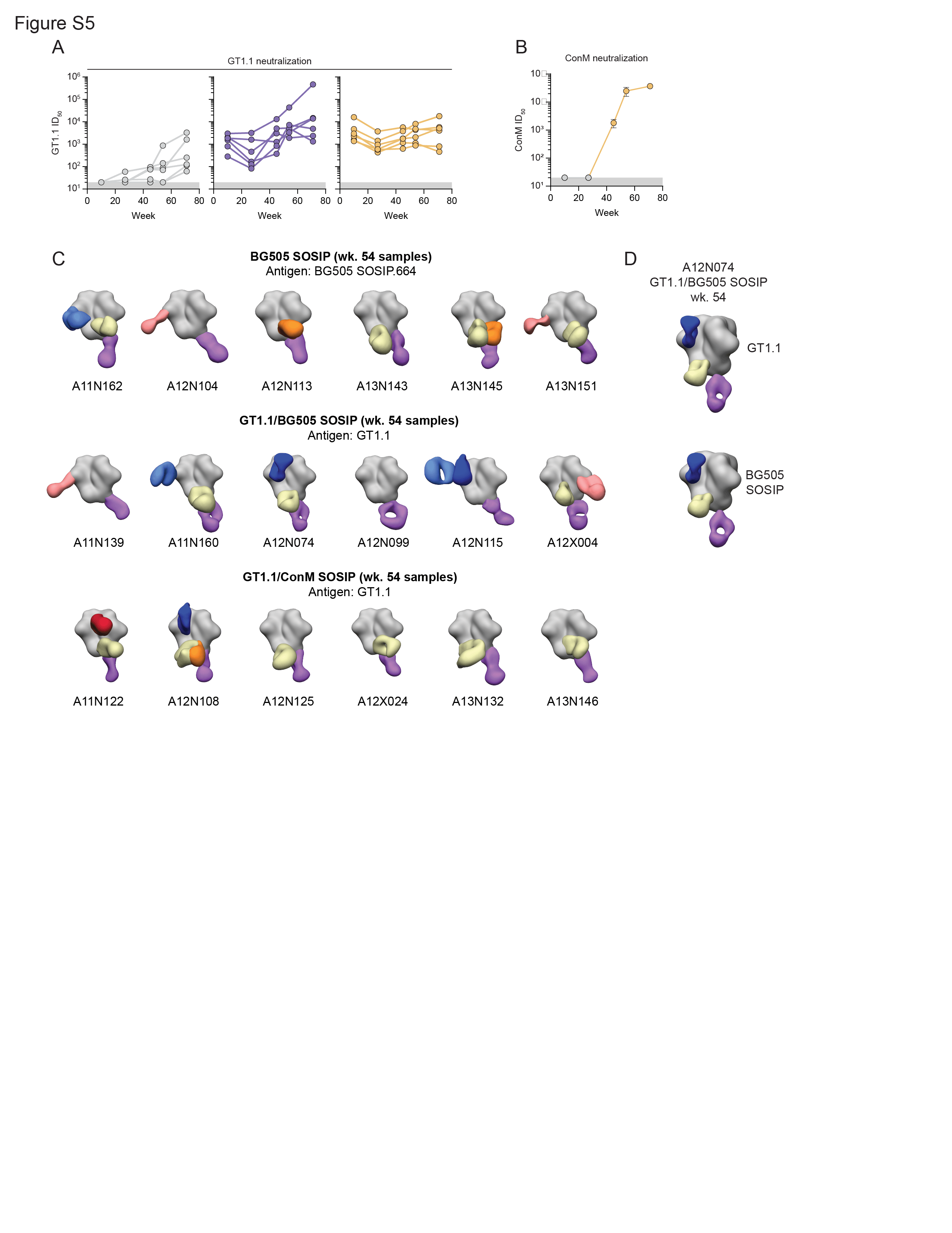
**

**Figure S5. Additional serum neutralization and EMPEM data. Related to Figure 3.** (A) Serum ID_50_ titers to GT1.1 for each of the immunization groups in Fig. 3. Each line represents one animal. (B) Serum ID_50_ titers to ConM pseudovirus for each of the immunization groups in Fig. 3. Each line represents one immunization group. (C) EMPEM 3D composite maps for each individual animal from the immunization groups in Fig. 3. (D) EMPEM 3D composite maps for animal A12N074 at week 54. The top panel represents Fabs derived from serum complexed with GT1.1, and the bottom panel represents serum Fabs complexed with BG505 SOSIP.

**
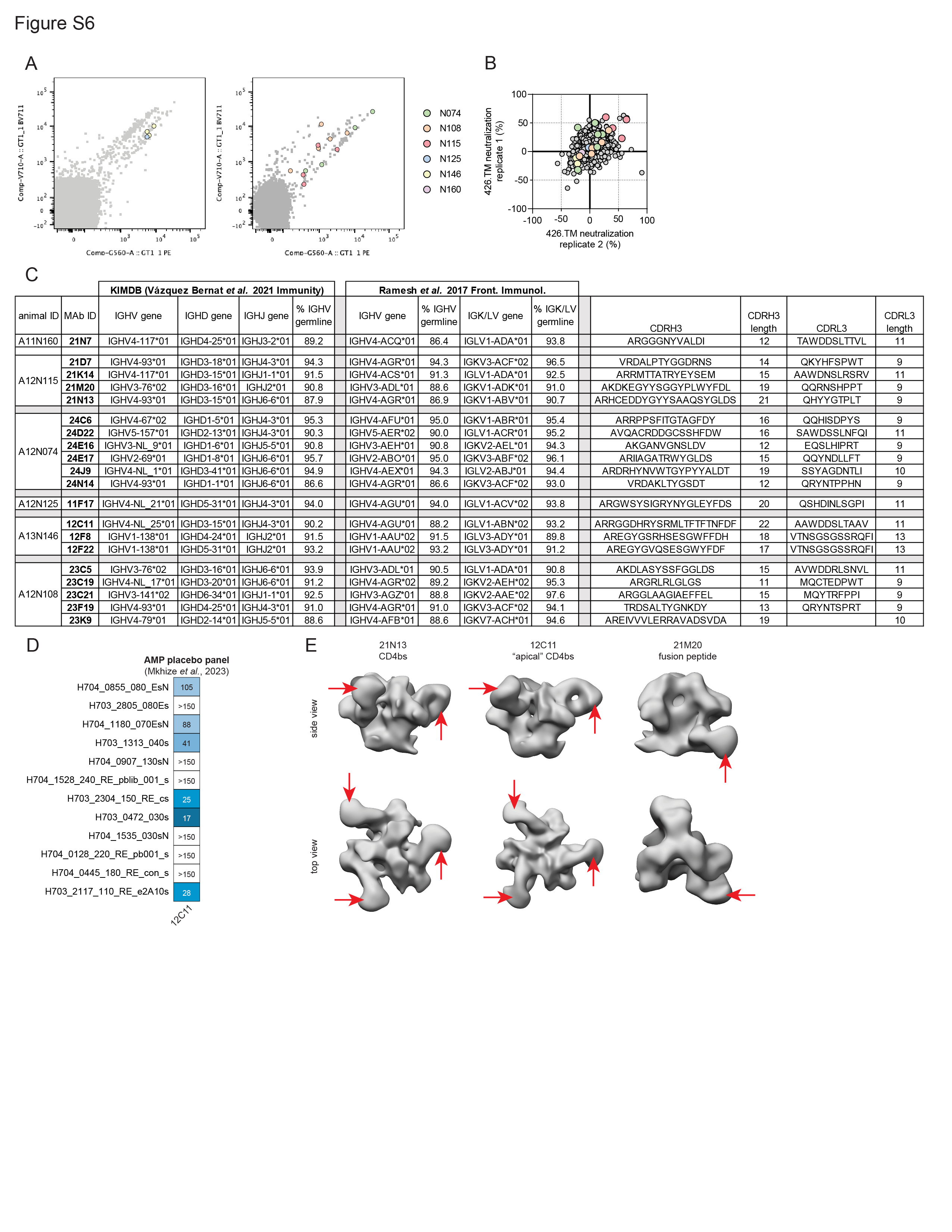
**

**Figure S6. Monoclonal antibody isolation from immunized non-human primates. Related to Figures 4-6.** (A) Index flow cytometry plots showing the relative binding of isolated monoclonal antibodies from each of the animals. Each dot represents a B cell, where colored dots highlight the animal the B cell originated from. (B) 426c.TM pseudovirus microneutralization for isolated monoclonal antibodies. Each dot represents a single B cell, with the colored dots corresponding to monoclonal antibodies from the indicated animals. (C) Table showing the basic genetic characteristics of the 20 selected monoclonal antibodies. (D) Table showing the IC_50_ titers of 12C11 against the indicated heterologous pseudoviruses, which are part of the AMP placebo panel. (E) Negative-stain electron microscopy 3D reconstructions showing the side view (top row) and top view (bottom row) for each indicated monoclonal antibody in complex with GT1.1. The red arrows point towards the densities that correspond to the monoclonal antibodies.

**
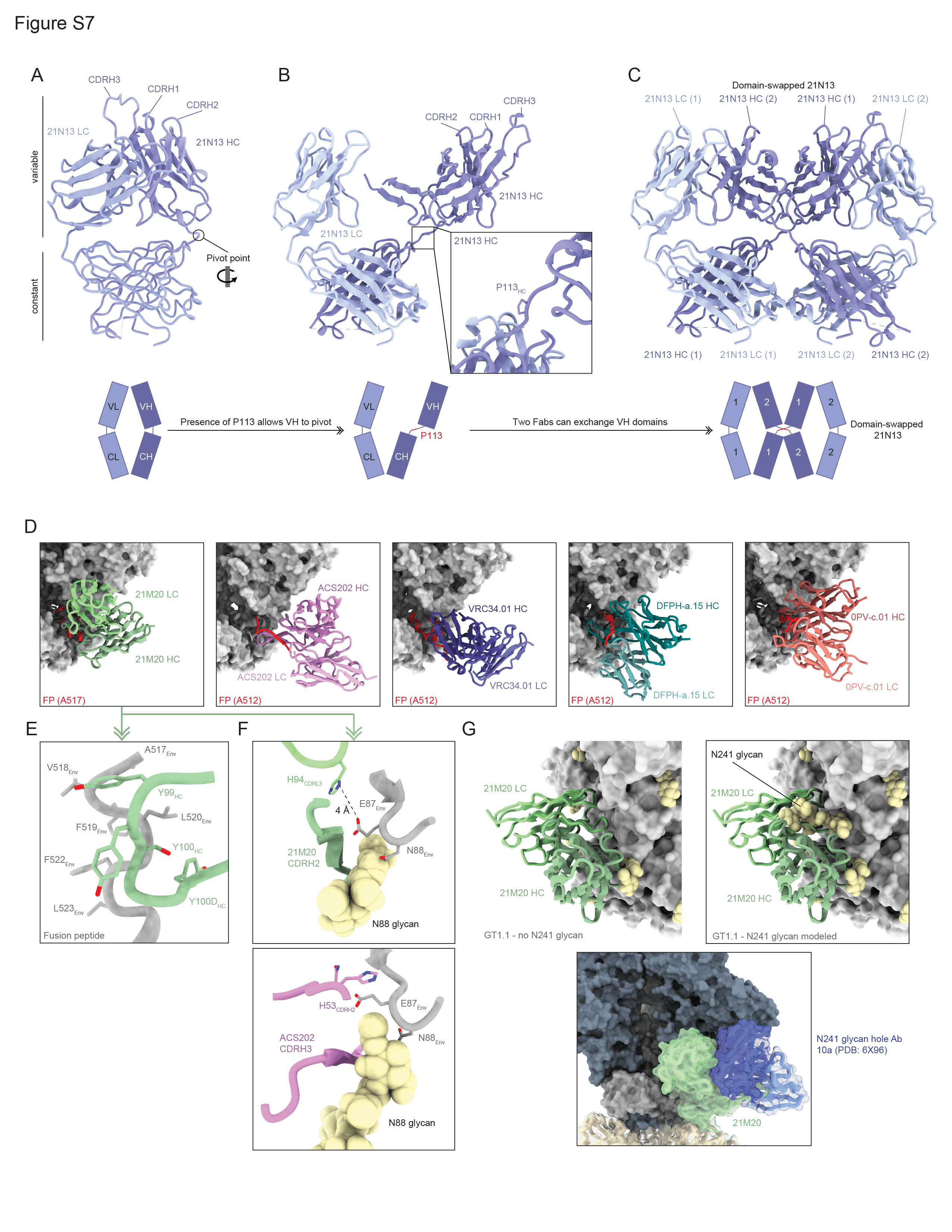
**

**Figure S7. NAb 21N13 forms domain-swapped Fab dimers and NAb 21M20 targets a fusion peptide-proximal epitope. Related to Figures 4 and 5.** (A) A 2.4 Å resolution crystal structure of unliganded 21N13 Fab (top panel) and a schematic representation of the VL, VH, CL and CH1 domains of the Fab. The CDRs are indicated, as well as the location of the P113 “pivot point”, used by 21N13 to form domain-swapped Fab dimers, similar to bNAb 2G12^59^. (B) Using the pivot point, the VH domain can change its configuration. The bottom panel depicts a schematic representation of the Fab domains as in (A). (C) With this “open” Fab conformation, two of these structures can dimerize, forming Fab dimers. The bottom panel depicts a schematic representation of a dimerized Fab. (D) 21M20 targets a fusion peptide (FP)-proximal epitope, but does not displace the FP as is commonly seen for FP bNAbs ACS202 and VRC34 as well as non-human primate-derived FP NAbs DFPH-a.15 and 0PV-c.01. The 1:1 stoichiometry is unusual compared to human gp120-gp41 interface bnAbs, which all bind with a 3:1 stoichiometry, with the exception of PGT151 that binds with a 2:1 stoichiometry (also see Fig. 4D). The HC contributes most of the BSA to the GT1.1 trimer (985 Å^2^ and 310 Å^2^ for HC and LC, respectively) (also see Fig. 5B). Furthermore, the 21M20 epitope spans across Env subunits and across protomers, with its HC contacting two gp41 protomers (377 and 225 Å^2^ BSA) and one gp120 protomer (382 Å^2^ BSA), implying that trimer-based immunization strategies will be necessary to induce such specificities. (E) Detailed interactions of 21M20 with the fusion peptide. It targets the hydrophobic fusion peptide using hydrophobic interactions, similar to FP bNAbs. (F) The CDRH2 of 21M20 accommodates the N88 glycan and its CDRL3 is positioned to contact E87_Env_ (top panel). This interaction is similar to FP-targeting bNAb ACS202, which also contacts/accommodates the N88 glycan and uses a histidine residue to contact the negatively charged E87_Env_ residue. (G) The limited breadth of 21M20 can be explained by its dependency on the absence of the highly conserved N241 glycan. When modeled onto GT1.1, the N241 glycan clashes with both heavy and light chains of 21M20. Although 21M20 cannot accommodate this glycan, it is not an N241 glycan hole antibody, as a typical N241 glycan hole antibody (10a, PDB 6X96) targets a different epitope.

**
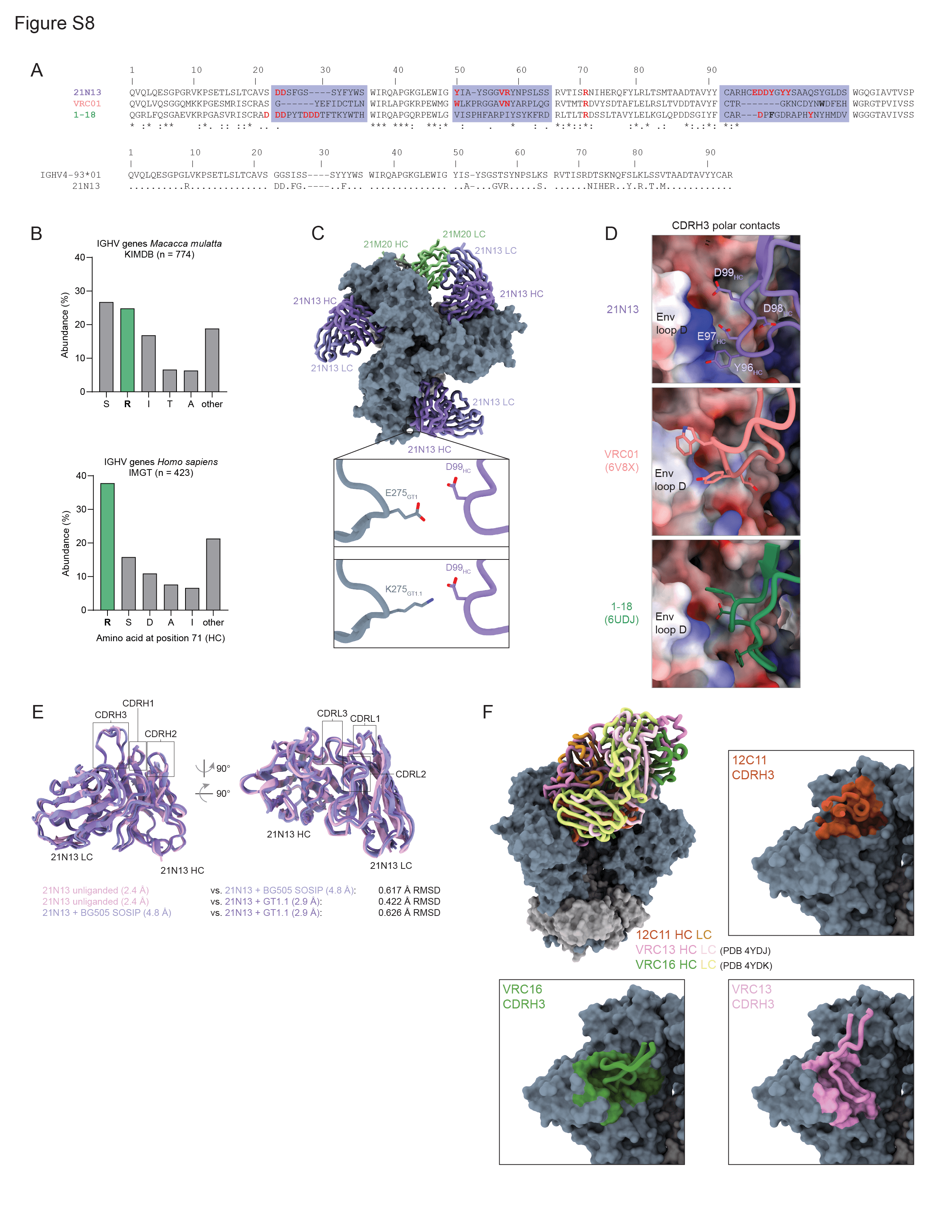
**

**Figure S8. Additional cryo-EM details of 21N13 and 12C11.** (A) Amino acid alignment of 21N13 with human bNAbs VRC01 and 1-18 (top) and with its predicted germline gene (bottom). (B) Presence of R71_HC_ in germline IGHV genes in macaques (top panel) and humans (bottom panel). While R71_HC_ is the most predominant residue in human germline IGHVs (37.8%), in NHPs the most common amino acid found at this position in NHPs is a serine followed by an arginine at 24.8%. (C) The negatively charged D99_HC_ residue extends towards the positively charged K275_Env_ residue in GT1.1. GT1.1 is different from its predecessor, GT1, only by the E275K mutation, which might have selected for a negatively charged residue in the CDRH3 of NAb 21N13. (D) Inset of the negatively charged CDRH3 of 21N13 (top panel) contacting a positively charged region on GT1.1, mostly mediated by the R278 and K275 residues present in GT1.1. These positively charged residues are not present in BG505 SOSIP but are incorporated in GT1.1 to make additional contacts with negatively charged conserved residues in VRC01-class precursors. (E) Overlay of the variable fragment of unliganded 21N13 (pink), 21N13 in complex with GT1.1 (dark purple) and 21N13 in complex with BG505 SOSIP (light purple). There are no distinct morphological differences between liganded and unliganded 21N13, as represented by the low root mean square deviations (RMSDs), indicating that the Fab does not substantially change conformation when bound. (F) Overlay of CDRH3-dominated CD4bs bNAbs VRC13 (PDB 4YDJ) and VRC16 (PDB 4YDK) with the 12C11-GT1.1 cryo-EM structure as described in Fig. 6. The insets show the CDRH3s of each NAb contacting the GT1.1 trimer. 12C11 contacts a more apical epitope than CDRH3-dominated bNAbs VRC13 and VRC16.

**
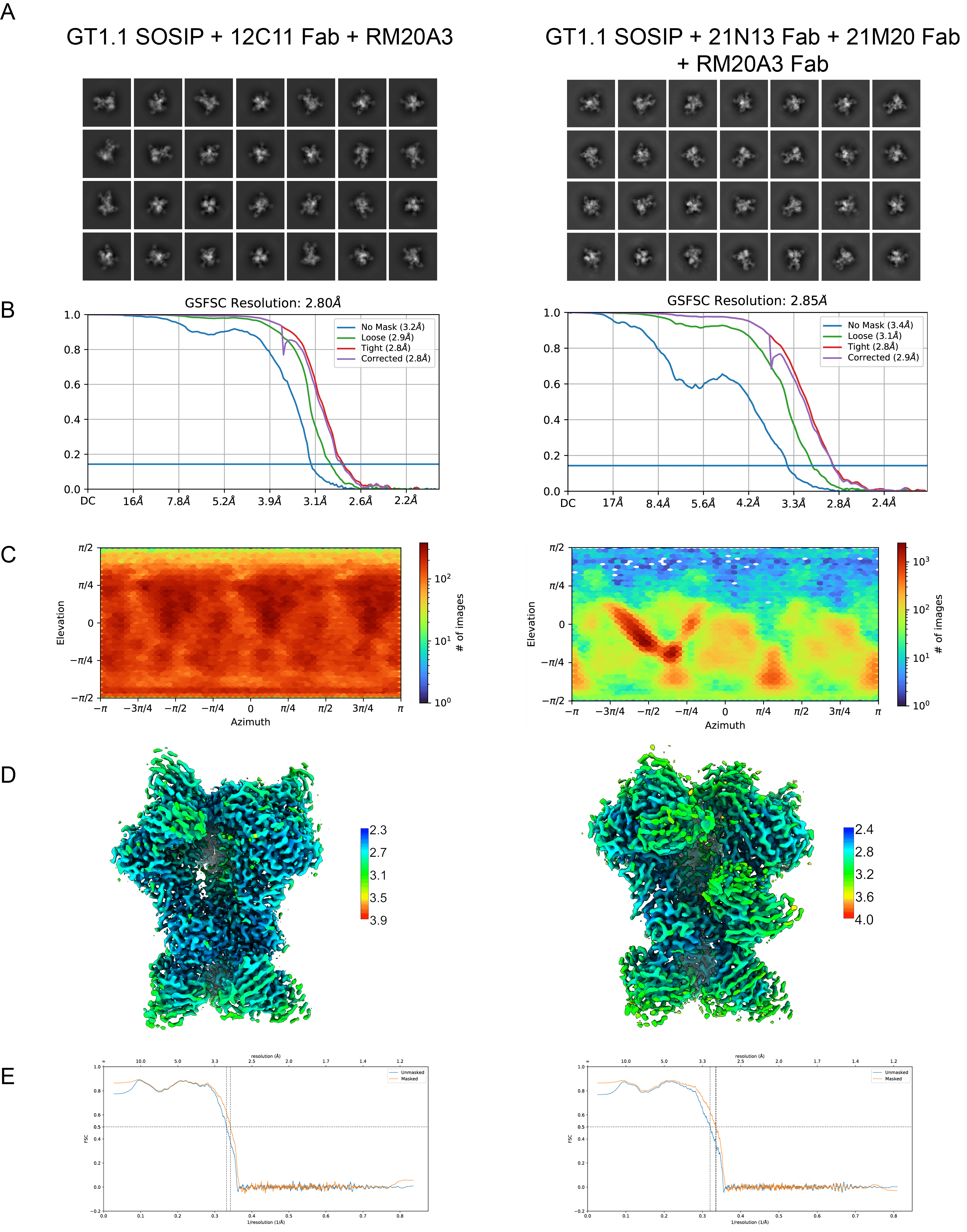
Figure S9. CryoEM reconstruction statistics. (**A) Representative 2D class averages. (B) Fourier shell correlation resolution estimate. (C) Angular distribution of observations. (D) Local resolution estimation (in Å). (E) Map to atomic model Fourier shell correlation.

**Table S1. SPR analysis of antibody binding to BG505 GT1 and GT1.1.**

|  | | | | | |  |  |  |  |
| --- | --- | --- | --- | --- | --- | --- | --- | --- | --- |
| **NAb (IgG)** | | **Env trimer** | ***k_on1_*** | ***k_off1_*** | ***K_D1_*** | ***k_on2_*** | ***k_off2_*** | ***K_D2_*** | ***S_m_*** |
|  |  |  | [1/Ms] | [1/s] | **[**nM] | [1/Ms] | [1/s] | [nM] |  |
| **VRC01** | *mature* | BG505 SOSIP.v4.1-GT1 | 2.2 **^.^** 10^5^ | < 10^-5^ | < 0.1 | 1.6 **^.^** 10 ^3^ | 1.8 **^.^** 10 ^-3^ | 1.3 **^.^** 10^3^ | 2.3 |
|  |  | (n=2) | ± 2.5 **^.^** 10^4^ |  |  | ± 4.6 **^.^** 10^2^ | ± 9.2 **^.^** 10 ^-4^ | ± 9.4 **^.^** 10^2^ | ± 5.5 **^.^** 10^-2^ |
|  |  | BG505 SOSIP.v4.1-GT1.1 | 2.2 **^.^** 10^5^ | < 10^-5^ | < 0.1 | 1.4 **^.^** 10^3^ | 2.5 **^.^** 10^-3^ | 1.8 **^.^** 10^3^ | 2 |
|  |  | (n=2) | ± 5.0 **^.^** 10^3^ |  |  | ± 53 | ± 1.5 **^.^** 10 ^-4^ | ± 1.8 **^.^** 10^2^ | ± 4.6 **^.^** 10^-3^ |
|  | *germline* | BG505 SOSIP.v4.1-GT1 | 9.4 **^.^** 10^3^ | 1.7 **^.^** 10^-2^ | 1.8 **^.^** 10^3^ | 8.6 **^.^** 10^2^ | 8.5 **^.^** 10 ^-4^ | 1.0 **^.^** 10 ^3^ | 2.3 |
|  |  | (n=2) | ± 1.7 **^.^** 10^2^ | ± 7.5 **^.^** 10^-4^ | ± 1.1 **^.^** 10^2^ | ± 2.1 **^.^** 10^2^ | ± 8.2 **^.^** 10 ^-5^ | ± 1.6 **^.^** 10 ^2^ | ± 5.1 **^.^** 10^-2^ |
|  |  | BG505 SOSIP.v4.1-GT1.1 | 1.2 **^.^** 10^4^ | 4.2 **^.^** 10^-3^ | 3.6 **^.^** 10^2^ | 4.1 **^.^** 10^3^ | 1.9 **^.^** 10 ^-3^ | 4.6 **^.^** 10^2^ | 2.1 |
|  |  | (n=2) | ± 4.0 **^.^** 10^2^ | ± 6.0 **^.^** 10^-5^ | ± 18 | ± 1.5 **^.^** 10^2^ | ± 5.0 **^.^** 10 ^-5^ | ± 4.6 | ± 1.4 **^.^** 10^-2^ |
| **3BNC60** | *mature* | BG505 SOSIP.v4.1-GT1 | 2.5 **^.^** 10^5^ | < 10^-5^ | < 0.1 | 2.8 **^.^** 10 ^3^ | 1.4 **^.^** 10 ^-3^ | 5.3 **^.^** 10^2^ | 2.4 |
|  |  | (n=3) | ± 4.8 **^.^** 10^4^ |  |  | ± 2.0 **^.^** 10^2^ | ± 6.7 **^.^** 10 ^-4^ | ± 3.0 **^.^** 10 ^2^ | ± 6.9 **^.^** 10^-3^ |
|  |  | BG505 SOSIP.v4.1-GT1.1 | 2.1 **^.^** 10^5^ | < 10^-5^ | < 0.1 | 3.2 **^.^** 10^3^ | 9.3 **^.^** 10^-4^ | 3.1 **^.^** 10^2^ | 2.2 |
|  |  | (n=3) | ± 1.9 **^.^** 10^4^ |  |  | ± 3.1 **^.^** 10^2^ | ± 1.8 **^.^** 10^-4^ | ± 72 | ± 4.2 **^.^** 10^-2^ |
|  | *germline* | BG505 SOSIP.v4.1-GT1 | Minimal binding: <10 RU | | | | | | |
|  |  | (n=2) |  |  |  |  |  |  |  |
|  |  | BG505 SOSIP.v4.1-GT1.1 | 3.9 **^.^** 10^4^ | 3.0 **^.^** 10^-2^ | 7.8 **^.^** 10^2^ | 6.4 **^.^** 10^2^ | 2.1 **^.^** 10^-3^ | 3.3 **^.^** 10^3^ | 1.1 |
|  |  | (n=2) | ± 2.5 **^.^** 10^3^ | ± 6.5 **^.^** 10^-4^ | ± 67 | ± 26 | ± 8.0 **^.^** 10^-5^ | ± 2.6 **^.^** 10^2^ | ± 3.7 **^.^** 10^-2^ |
| **12A12** | *germline* | BG505 SOSIP.v4.1-GT1 | Minimal binding: <10 RU | | | | | | |
|  |  | (n=2) |  |  |  |  |  |  |  |
|  |  | BG505 SOSIP.v4.1-GT1.1 | 4.2 **^.^** 10^4^ | 8.5 **^.^** 10^-3^ | 2.0 **^.^** 10^2^ | 5.7 **^.^** 10^8^ | 1.1 **^.^** 10^2^ | 1.9 **^.^** 10^2^ | 1.6 |
|  |  | (n=2) | ± 1.0 **^.^** 10^3^ | ± 2.0 **^.^** 10^-4^ | ± 9.6 | ± 1.4 **^.^** 10^8^ | ± 30 | ± 7.4 | ± 0.15 |
| **PGV19** | *germline* | BG505 SOSIP.v4.1-GT1 | 4.1 **^.^** 10^4^ | 3.7 **^.^** 10^-4^ | 9.1 | 2.1 **^.^** 10^3^ | 1.7 **^.^** 10^-3^ | 8.1 **^.^** 10^2^ | 2.3 |
|  |  | (n=2) | ± 3.5 **^.^** 10^2^ | ± 1.0 **^.^** 10^-6^ | ± 0.10 | ± 15 | ± 1.5 **^.^** 10^-5^ | ± 13 | ± 5.4 **^.^** 10^-3^ |
|  |  | BG505 SOSIP.v4.1-GT1.1 | 5.3 **^.^** 10^4^ | 1.3 **^.^** 10^-3^ | 24 | 1.6 **^.^** 10^4^ | 2.2 **^.^** 10^-3^ | 4.6 **^.^** 10^2^ | 2.6 |
|  |  | (n=2) | ± 1.5 **^.^** 10^2^ | ± 6.7 **^.^** 10^-4^ | ± 12 | ± 1.4 **^.^** 10^4^ | ± 3.3 **^.^** 10^-4^ | ± 3.8 **^.^** 10^2^ | ± 0.32 |

| ***^a^*** A bivalent model was fitted to the binding data for the specific response in response units [RU] over time [s]: ***k_on1_*** [1/Ms] = on-rate constant for the first-point interaction; ***k_off1_*** [1/s] = off-rate constant for the first-point interaction, 10^-5^ [1/s] being the limit of detection; ***K_D1_*  [**nM] = equilibrium-dissociation constant for the first-point interaction; ***k_on2_*** [1/Ms] = on-rate constant for the second-point interaction converted from [1/RUs]; ***k_off2_*** [1/s] = off-rate constant for the second-point interaction; ***K_D2_*  [**nM] = equilibrium-dissociation constant for the second-point interaction converted from [RU]; ***S_m_*** = the stoichiometric value of paratopes per trimer; n signifies the number of replicates. |
| --- |

**Table S2. GT1.1 and GT1.2 pseudovirus characterization and tier phenotyping.**

**Table S3. Nucleotide sequences of monoclonal antibodies isolated from immunized NHPs.**

| Ab ID | Animal | HC sequence (nt) | LC sequence (nt) |
| --- | --- | --- | --- |
| 11F17 | A12N125 | CAGGTGCAGCTGCAGGAGTCGGGCCCAGGACTGGTGAAGCCTTCGGAGACCCTGTCCCTCAGTTGCGTTGTCTCTGATTACTCCATCAGCAGTGCTTTTGGCTGGAGCTGGATCCGCCAGCCCCCAGGGAAGGGGCTGGAGTGGATTGCTCATATCGGTGGTAGTAGTGATGACACGAGCTACAATCCCTCCCTCAAGAGTCGAGTCGCCATTTCAAAAGACACGTCCAAGAATCAGTTCTCCCTGAAGCTGAGCTCTGTGACCGCCGCGGACACGGCCGTATATTACTGTGCGAGAGGGTGGAGTTATTCTATTGGACGTTATAACTACGGTCTAGAGTACTTTGACTCCTGGGGCCAGGGAGTCCTGGTCACCGTCTCTTCA | CAGTCTGTGCTGACACAGCCGCCCTCAGTGTCTGGGGCCCCAGGACAGAGGGTCACCATCTCCTGCACTGGGACTAGTTCCAACATTGGACGTCATTATGTATACTGGTACCAGCACCTCCCAGGAGTGGCCCCCAAAGTCCTCATCTATGACACTAATAAGCGACCATCAGGGGTTTCTGACCGATTCTCTGGCTCCAAGTCTGGTTCCTCAGCCTCCCTGACCATCACTGGGCTCCAGACTGAGGATGAGGCTGATTATCACTGCCAGTCCCATGACATCAACCTGAGTGGCCCCATCTTCGGTGCTGGGACCCGGCTCACCGTCCTA |
| 12F22 | A12N146 | CAGGTCCAGCTGGTGCAGTCCGGGGCTGAGGTGAAGAAGCCTGGGGCCTCAATGCAGCTCTCCTGCAAGGCTTCTGGATACAACTTCGGCCTCTATGCCATCAATTGGGTGCGACAGGCCCCTGGACAAGGCCTTGAGTGGATGGGAGGGATCATCCCTCTTATTGGTTTAACATACTATGCACAGAAGTTCCAGGGCAGAGTCACAATTAGCGCGGACACGTCCACAAACACAGCCCACATGGAGCTGAGCAGCCTGGGATCTGAAGACACCGCCGTCTATTTCTGTGCGAGAGAGGGCTACGGTGTCCAGTCCGAGTCGGGCTGGTATTTCGATTTCTGGGGCCCTGGCACCCCAGTCACCATCTCCTCA | TCCTATGAGGTGACTCAGTCTCCCTCAGTGTCGGTGTCCCCCGGACAGACAGCCAGCATCACCTGCTCTGGAGATGGGTTTGGGACTGCATATGGTGACTGGTACCAGCAGAAGCCAGGCCAGGTCCCTGTTGCGGTCATCTATAAAAATACTAACCGGCCCTCAGGGATCCCTGAGCGATTCTCTGGCTCCACCTCGGGGAACACGGCCACCCTGACCATCAGCGGGGTCGAGGCTGGGGATGAGGCCGACTATTATTGTGTCACAAATTCTGGCTCCGGGAGCAGCAGGCAGTTCATCTTCGGTCCTGGGACCCGGCTCACCGTCCTA |
| 12C11 | A12N146 | CAGGTCCAGCTGCAGGAGTCGGGCCCAGGACTGGTGAAGCCTTCAGAGACCCTGTCCCTCACCTGCGCTGTCTCTGGCAACCCTGTCACCGCTGGTTTTGACTGGACCTGGATCCGCCAGTCCCCGGGGAAGGGGCTGGAGTGGATTGGCCATATCTATGGTGCTAGTGGGAGCACCAATTACAATCCGTCCCTCGAGAGTCGAGTCACCATTTCAAGGGACGCGTCCAGGAACCAGTTCTCCCTGAAACTGACCACTGTGACCGCCGCGGACACGGCCGTCTATTACTGCGCGAGACGAGGGGGGGATCATCGGTATTCGAGGATGCTTACATTTACTTTTACAAACTTCGACTTTTGGGGCCAGGGAGTCCTGGTCACCGTCTCCTCA | CAGTCTGTGCTGACTCAGCCTCCCTCAGTGTCTGAGGCCGCCAGGAAGAGTGTCTCCATCTCCTGTTCTGGTAGCGACTCCAACATCGGCAGTAATAGTGTCTCCTGGTTCCAGCAGTTCCCAGGAACAGCTCCCAAACTCCTCATTCATTTTAATAATCAACGAGCCTCAGGTGTTTCTGACCGATTCTCTGGCTCCAAGTCTGGCACGTCTGCCTCCCTGGCCATCAGTGGACTCCAGACCGACGATGAGGCTGATTATTACTGCGCAGCTTGGGATGATAGCCTGACCGCTGCCGTCTTCGGTACTGGGACCCGGCTCACCGTCCTA |
| 12F8 | A12N146 | CAGGTCCAGCTGGTGCAGTCCGGGGCTGAGGTGAAGAAGCCTGGGGCCTCAATGCAGCTCTCCTGCAAGGCTTCTGGATACAACTTCGGCCTCTATGCCATCAATTGGGTGCGACAGGCCCCTGGACAAGGCCTTGAGTGGATGGGAGGGATCATCCCTCTCAATGGATTAACATATTATGTACAGAAGTTCCAGGGCAGACTCACCATGACCGCGGACACGTCCACAAATACAGCCCACATGGAGCTGAGCAGCCTGAGATCTGAGGACACCGCCGTTTATTTCTGTGCGAGAGAGGGCTACGGTAGTAGACACTCCGAGTCGGGCTGGTTTTTCGATCACTGGGGCCCTGGCACCCCAGTCACCATCTCCTCA | TCCTATGAGGTGACTCAGTCTCCCTCAGTGTCGGTGTCCCCCGGACAGACAGCCACCATCACCTGCTCTGGAGATGGGTTTGGGACTACATATGGTGACTGGTACCAACAGAAGCCAGGCCAGGTCCCTGTTGCGGTCATCTATAAAAATACTCACCGGCCCTCAGGGATCCCTGAGCGATTCTCTGGCTCCACCTCGGGGAACACGGCCACCCTGACCATCAGCGGGGTCGAGGCTGGGGATGAGGCCGACTATTATTGTGTCACAAATTCTGGCTCCGGGAGCAGCAGGCAGTTCATCTTCGGTCCTGGGACCCGGCTCACCGTCCTA |
| 21N7 | A11N160 | CAGGTGCAGCTGCAGGAGTCGGGCCCAGGACTGGTGAAGCCTTCGGAGACCCTGTCCCTCACCTGCGCTGTCTCCGGTGGCCCCATCAGCAGTGGTCACTACTACTGGACCTGGATCCGCCAACCCCCAGGGAAGGGACTGGAGTGGATAGGGTTCATCACTTATGGTGGGACCACCGACTACAACCCGTCCCTCAAGAGTCGAGTCACCTTTTCAACAGACACGTCCAAGAACCAGTTCTCCCTGAAGCTGAATTCTGTGACCGCCGCGGACACGGCCGTGTATTATTGTGTGAGAGATGCCCTCCCCACCTATGGTGGTGATAGAAACTCGTGGGGCCGGGGAGTCCTGGTCACCGTCTCCTCA | CAAGTTATATTGACTCAGTCTCCGGCCACCCTGTCTTTGTCACCGGGGGAAAGAGCCACCCTCTCCTGCAGGGCCAGTCAGAGTATTGGCAGCAGCTTAGCCTGGTACCAGCAGAAACCTGGGCAGGCCCCCAGGCTCCTCATCTATGGTTCATCCAGCAGGGCCACTGGCATCCCAGACAGGTTCAGTGGCAGTGGGTCTGGGACAGAGTTCACTCTCACCATCAGCAGCCTGGAGCCTGAAGATTTTGCTGTGTATTACTGTCAGAAGTATCATTTCTCCCCGTGGACGTTCGGCCAAGGGACCAAGGTGGAAATCAAA |
| 21D7 | A12N115 | CAGGTGCAGGTGCAGGAGTCGGGCCCAGGACTGGTGAGGCCTTCGGAGACCCTGTCCCTCACCTGCGTTGTCTCTGGTGGCTCCATCGGCACTAACTACTGGAGCTGGGTCCGCCAGTCCCCAGTGAAGGGACTGGAATGGATTGGACAAATCTATCTTAATTTTGGGACTACCAGTTATAATCCCTCCCTCACAAGTCGAGCCACCATTTCAGCGGACATGTCCAAGAACCACTTCTCCCTGATACTGAGTTCTGTGACCGCCGCGGACACGGCCGTCTATTATTGTGCGAGAGGGGGCGGTAACTACGTTGCTCTTGATATCTGGGGCCCAGGGCTCAGGGTCACAGTCTCTTCA | CAGTCTGTGCTGACTCAGCCACCCTCAGCGTCTGGGGCTCCCGGGCAGAGTGTCACCATCTCTTGTTCTGGAGGCAGATCCAACATTGGAAATAATTATGTCTACTGGTACCAACACCTCCCAGGGACGGCCCCCAAAGTCGTCATCTATTATACTAATCAGCGACCCTCAGGGGTCCCTGACCGATTCTCTGGCTCTAAGTCCGGCACCTCAGCCTCCCTGGCCATCACTGGTGTCCGATCTGAGGATGAGGCTGATTATTACTGTACAGCCTGGGATGACAGTCTGACCACTGTTTTATTCGGAGGAGGGACCCGGTTGACCGTCCTG |
| 21M20 | A12N115 | GAGGTGCAGTTGGTGGAGTCTGGGGGCGGCTTGGCAAAGCCTGGGGGATCCCTGAGACTCTCCTGTGCAGCCTCAGGATTCACCTTCAGTAGGAGTATTATGCACTGGGTCCGCCAGGCTCCAGGGAAGGGGCTGGAATGGGTTTCAACTATTAATTATGCTGGTAGTACATACTACGCCGACTCCGTGAGGGGCCGATTCGTCATCTCCAGGGACAACTCAAAGAACACGCTCTCCCTACAAATGAACAGCGTGCGACCTGAGGACAGGGCCGTGTATCACTGTGCGAAGGATAAAGAGGGTTACTATAGTGGTGGTTATCCCTTATGGTACTTCGATCTCTGGGGCCCTGGCACCCCTGTCACCATCTCCTCA | GACGTCCAGATGACCCAGTCTCCTTCCTCCCTGTCTGCCTCTGTTGGTGACAAAGTCAGCATCACTTGCCGGGCCAGTCAGGGCATTGCCGATGCTTTAGCCTGGTATCAGCAGAAACCAGGGAAAGCCCCTAAACTCCTGATCTATGGTGCATCCAACCTGGAAAGTGGGGTCCCATCAAGATTCAGCGGCAGTGGGTCTGGGACAGATTTCAGTCTCACCATCAGTAGCCTGCAGGCTGAAGATTTTGCAGTCTATTACTGTCAACAGCGCAACAGTCACCCTCCGACGTTCGGCCAAGGGACCAGGGTGGAAGTCAAA |
| 21N13 | A12N115 | CAGGTGCAGCTTCAGGAGTCGGGCCCAGGGCGGGTGAAGCCTTCGGAGACCCTGTCCCTCACCTGCGCTGTCTCTGATGACTCTTTCGGTAGTAGTTATTTCTACTGGAGTTGGATCCGCCAGGCCCCAGGGAAGGGACTGGAGTGGATTGGGTACATCGCTTACAGTGGGGGCGTCAGATACAACCCGTCCCTGTCGAGTCGAGTCACCATTTCAAGAAACATACACGAGAGACAGTTCTACCTGCGTCTGACCTCTATGACCGCCGCGGACACGGCCGTCTACTACTGTGCGAGACATTGCGAAGATGATTACGGTTACTATTCCGCCGCCCAGTCATATGGTTTGGATTCCTGGGGCCAAGGGATCGCCGTCACCGTCTCCCCA | GACATCCAGATGACCCAGTCTCCATCCTCCCTGTCTGCATCTGTAGGAGACAGAGTCACCATCACTTGCCGGACAAGTGAGAACGTTAACAACTGTTTAAATTGGTATCAACAAAAACCAGGGAAAGCCCCTAAGCTCCTGATCTATAGGACATCCACTTTGCAGAGAGGGGTCCCATCAAGGTTCAGCGGCACTGGATCTGGGACAGATTACACTCTCACCATCAGCAGCCTGCAGTCTGAGGACTTTGGCACTTACTACTGTCAACATTATTATGGTACCCCGCTCACTTTCGGCGGAGGGACCATGGTGGACATCAAA |
| 21K14 | A12N115 | CAGCTGCAGCTGCAGGAGTCGGGCCCAGGACTGGTGAAGCCTTCGGAGACCCTGTCCCTCACCTGCGTTGTCTCTGGTGGCTCCATCAGCAGTGGCTACTGTGCCTGGATCCGCCAGCCCCCCGGGAAGGGACTCGAGTGGATTGGACGTATCTCGGATGTTACTGGAACCGCCGACTATAATCCCTCCCTCAAGAGTCGAGTCACCATTTCAATAGACACGTCCAAGAACCAGTTCTCCCTGACGTTGAGGTCTCTGACCGCCGCGGACACGGCCGTATATTACTGTGCGAGAAGGATGACTACAGCGACCAGATATGAATACTCCGAAATGTGGGGCCAGGGCGCCCTGGTCACCGTCTCCTCA | CAGTATGTGCTGACTCAGCCACCCTCAGTGTCTGGGGCTCCCGGTCAGAGTGTCACCATCTCTTGCTCTGGAAGCAGCTCCAACATTGGAAGTTATAATGTGTACTGGTATCAGCAGTTCCCAGGAGCGGCCCCCAGAGTCCTCATCTGGGAGAATATTCATCGACCCTCAGGGGTCCCTGACCGATTCTCTGGCTCTAAGTCTGGCACCTCAGCCTCCCTGGCCATCACTGGTCTCCGATCTGAGGATGAGGCTGATTATTATTGTGCAGCATGGGACAACAGTCTGAGGAGTCGGGTTTTCGGCGGAGGGACCCGGCTGACCGTCCTA |
| 23F19 | A12N108 | CAGGTGCAGTTACAGGAGTCGGGCCCAGGGCTGGTGAAGCCCTCGGAGACCCTGTCCCTCACCTGCGCTGTCTCTGGTGGTTCCATCAGCAGTGGTTTTTACTACTGGACCTGGATCCGCCAGCCCCCCGGGAAGGGACTGGAATGGATTGGATACATCACTCATACTGGGAATGCCGACTACAGCCCGTCCCTCGAGAGTCGAGTCACCATTTCCAGAGACACGTCGAAGAACCAGTTCTCCCTGACGCTGACGTCTGTGACCGCCGCGGACACGGCCGTATATTACTGTACGAGAGACTCCGCCCTGACCTACGGCAACAAAGATTATTGGGGCCAGGGAGTCCTGGTCACCGTCTCCTCA | CAAGTTATATTGACTCAGTCTCCAGCCACCCTGTCTTTGTCTCCAGGGGAAAGAGCCACCCTCTCTTGCAGGGCCAGTCAGAGTGTTGGCAGTGCCTTAGCCTGGTATCAGCAGAAACCTGGGCAGGCCCCCAGGCTCCTCGTCTATGGTGCTTCCAGCAGGGCCACTGACATCCCAGACAGGTTCAGTGGCAGTGGGTCTGGGACAGACTTCACTCTCACCATCAGCAGTCTAGAGCCTGAGGATTTTGCAGTGTATTATTGTCAGAGATATAATACCTCCCCTCGGACGTTCGGCCAAGGGACCAAGGTGGAAATCAAA |
| 23K9 | A12N108 | CAGGTGCAGCTGCAGGAATCGGGCCCAGGACTGGTGAAGCCTTCGGAGACCCTGTTCCTCACCTGCGCTGTCTCTGGTGGCTCCATGAGTGATGATTACTACTGGACCTGGATCCGCCAGTCCCCAGGGAAGGGTCTGGAATGGATTGGGTACATCTATGACGGAAGTGGGGCCACCAGTTACAACCCCTCATTCAAGAGTCGCGTCGCCATCTCAATTGACACGTCCAAGAACCAGTTCTCCCTGACCCTGAAGTCGGTCACCGCCGCGGACACGGCCACATATTTCTGTGCGAGAGAGATTGTAGTCGTCCTCGAGAGAAGGGCGGTGGCGGACTCAGTGGATGCCTGGGGCCGGGGAATTGCGGTCACCGTCTCCTCA | GACATTGTGCTGACCCAGTCTCCAGCCTCTTTGGCCGTGTCTCCAGGGCAGAGGGCCACCATCACCTGCAGAGCCAGCGAGAGTGTCAGTTCGTTTGGAGTATACCTCATTCACTGGTATCAGCAGAAACCAGGACAATCTCCTAGACTCCTCATTTACCAGGCAACCAATAAAGACACTGGGGTCCCAGTCAGGTTCAGCGGCAGTGGGTCTGGGACCGATTTCACCCTCACAATTGATCCTGTGGAGCCTGACGATGCTGCAGATTATTATTGTCTGCAGACTAAGAATTCTCCTCGGACGTTCGGCCAAGGGACCAAGGTGGACATCAAA |
| 23C5 | A12N108 | GAGGTGCAACTGGTGGAGTCTGGGGGCGGCTTGGCAAAGCCCGGGGGATCCCTGAGACTCTCCTGTGAAGCCTCTGGATTCATCTTCAGTAGTTATGCTATGCACTGGGTCCGCCAGGCTCCGGGGAAGGGGCTGGTGTGGGTCTCAGGTGTCAATAGTAGTGGTGGCACATACTACGGAGACTCCGTGAAGGGCCGATTTACCATCTCCAGAGACAACTCAAAGAACACGCTCTCCCTGGAAATGAACAGCCTGAGAATTGAGGACACGGCCGTGTATTACTGTGCGAAAGATCTTGCTAGTTATTCCTCGTTTGGCGGTTTGGATTCCTGGGGCCAAGGGGTCGTCGTCACCGTCTCCTCA | CACTCTGTGCTGACTCAGCCACCCTCAGCGTCTGGGACTCCCGGCCAGAGTGTCTCCATCTCTTGCTCTGGAAGCTACTCCAATATTGGAGGTTATTTTGTATATTGGTACCAGCAGTTGCCAGGGGCGGCCCCCAAACTCCTCATCCATTATAATGATCAGCGACCCTCAGGGGTCCCTGACCGATTCTCTGGCTCCAAGTCTGGCAGGTCAGCCTCCCTGGCCATCACTGGTCTCCGGTCTGAAGATGAGGCTGATTATTACTGTGCAGTTTGGGATGACAGACTGAGCAATGTGTTATTCGGAGGAGGGACCCGGCTGACCGTCCTA |
| 23C19 | A12N108 | CGGATGCAGCTGCAGGAGTCGGGCCCAGGAATGGTGAAGCCTTCGGAGACCCTGTCCCTCACCTGCGTTGTCTCTACTGGCTCCATCAGCAGTGGTTTCTACTACTGGACCTGGATCCGCCAGCCCCCGGGGAAGGGACTGGAGTGGATTGGTCTTATCCACAGTAATAATCAGAATACCAATTACAATCCCTCCCTCAAGAGTCGAGTCACCATTTCAAAAGACACGTCCAAAAATCTGTTCTCCCTGGAACTGCGCTCTGTGACCGCCGCGGACACGGCCATGTATTTCTGTGCGAGAGGTCGCCTACGATTGGGTTTGGGTTCCTGGGGCCAGGGGGTCGTCGTCACCGTCACCTCA | GATATTGTGTTGACCCAGACTCCACCCTCGTTGCCCGTCACCCCTGGAGAGCCGGCCTCCATCTCCTGCAGGTCTAGTCAAACCCTTCTACATAGTAATGGAATCACCTATTTGCATTGGTACCTCCAGAAGCCAGGCCAGTCTCCACGGCTCCTGATCTATAAAGTTACCAGTCGGGAATTTGGGGTCCCAGACAGGTTCAGTGGCAGTGGGTCAGGCACAGAGTTCACACTGAAAATCAGCAGGGTGGAGCCTGAGGATGTTGGTGTTTATTACTGCATGCAGTGTACAGAGGATCCGTGGACGTTCGGCCAAGGGACTAAGGTGGAAATCAAA |
| 23C21 | A12N108 | GAAGTGCGCCTGGTGGAGTCTGGGGGAGGCGTGGTACAGCCTGGGGGGTCCCTGAGACTCTTGTGTACAGCCTCTGGATTCACCTTTGATGATCATGCCATGCACTGGGTCCGCCAAGCTCCAGGGAAGGGTCTGGAGTGGATCTCTGATATTACCTGGAATGGTGGTAAGAAACACTATGGAGACTCCGTGAAGGGCCGATTCACCATTTCCAGAGACAACGGCAAGAACTCCCTGTATCTGGACATGAACAGACTGAGGCCTGAAGACACAGCCTTCTATTACTGTGCGCGAGGGGGTCTAGCAGCTGGGATCGCTGAGTTCTTCGAATTGTGGGGCCAGGGCGCCCTGGTCACCGTCTCCTCA | GATATTGTGATGACCCAGACTCCACTCTCCCTGCCCGTCACCCCTGGAGAGCCGGCCTCCATCTCCTGCAGGTCTAGTCAGAGCCTCTTGGATTCTGAAGATGGAAAGACCTATTTGGATTGGTACCTCCAGAAGCCGGGCCAGTCTCCACAGGCCTTGATTTATGAGGTTTCCAATCGGGCCTCTGGAGTCCCAGACAGGTTCAGTGGCAGTGGGTCAGACACTGATTTCACACTGAAAATCAGCAGAGTGGAGGCTGAGGATGTTGGGGTTTATTACTGCATGCAATATACACGTTTTCCTCCCATTTTCGGCGGAGGGACCAAGGTGGAGATCAAG |
| 24N14 | A12N074 | CAGGTGCAGTTGCAGGAGTCGGGCCCAGGACTGGTGAAGCCATCGGAGACCCTGTCCCTCACCTGCGATATTTCTGGTGACTCCTTCAGTCGTAGTATGTACTTCTGGAGTTGGATCCGCCAGGCCCCAGGGAAGGGACTGGAGTGGATTGGACACCTCACCTATGGAGGGACCACTAGTTACAATCCGTCCCTCGAGAGTCGAGTCACCATTTCAAGAGACACGTCCAAGAACCAGGTCTTCCTGAAGTTAAAGTCTGTGAGCGCCGCGGACGCGGCCATATATTTCTGTGTAAGAGATGCGAAGCTGACCTACGGTTCGGATACCTGGGGCCGAGGGGTCCGCGTCATCGTCTCCTCA | CAAGTTGTGTTGACTCAGTCTCCAGCCACCCTGTCTTTGTCTCCAGGGGAAAGAGCCACCCTCTCCTGCAGGGCCAGTCAGAGTGTTGGCAGCGCCTTAGCCTGGTACCAGCAGAAACCTGGACAGGCCCCCAGGCTCGTCATCTATGGTGCCTCTACTACGGCCCCTGGCATCCCTGACAGGTTCCGTGGCAGTGGGTCTGGGACAGAGTTCACTCTCACCATCAACAGCCTGGAGCCTGAAGATTTTGGACTATATTACTGTCAGAGGTACAACACCCCCCCGCACAATTTTGGCCAGGGGACCAGAGTAGACATGAAA |
| 24E16 | A12N074 | GAGGTGCAGCTGGTGGAGTCTGGGGGCGGCTTGGCGCAGCCTGGGGGGTCCCTGGGACTCTCCTGTGCAGCCTCTGGATTCAGGTTCACTAGATACTACATGTATTGGGTCCGCCAGACTCCAGGGAAGGGGCTGGAGTGGATCTCATCTCTTGATGCTGGTGGGGAAACCACATACTACTCAGACTCCGTGAAGGGCCGATTCACCATTTCCAGAGACAACTCAAAGAATACGGTCTCCCTACTAATGGTCAGCCTGAGACCTGAGGACACGGCCGTATATTACTGTGCGAAAGGCGCGAATGTTGGTAACTCATTGGATGTCTGGGGCCGGGGGGTTCTGGTCACCGTCTCCTCA | GATATTGTGATGACCCAGACTCCACTCTCCCTATCCGTCACCACTGGAGAGCCGGCTTCCATGTCCTGCAGGTCCAGTCAGAGACTCCTGCATAGTAATGGGAACACCTATTTGCATTGGTACTTGCAGAAGCCGGGCCAGTCTCCACAGCTCCTGATCTATGAGGTTTCCAACCGGGCCTCTGGAGTCCCTGACAGGTTCAGTGGCAGTGGGTCAGGCACTGATTTCACCCTGAGAATCAGCCGGGTGGAGGCTGAGGATGTTGGGATTTATTACTGCGAACAAAGTCTACACATTCCGCGCACTTTCGGCGGAGGGACCAAGGTGGATATCAAA |
| 24J9 | A12N074 | CAGGTGCAGCTGCAGGAGTCGGGCCCAGGACTAGTGAAGCCTTTGGAGACCCTGTCCCTCACCTGCACTGTCTCTGGTGGCTCTATCAGCAGTAACTACTGGAGTTGGATCCGCCAGCCCCCAGGAAAGGGACTGGAATGGATTGGATATATCTATGGCAGTGGTGGGAGCACCGACAGCAACCCCTCCCTCAAGAGTCGAGTCACCCTGTCAGTGGACACGTCCAAGAACCAGTTCTCCCTGAGGTTGAGTTCTGTGACCGCCGCGGACACGGCCGTCTATTACTGTGCGAGAGATCGCCATTACAATGTCTGGACTGGGTACCCATATTACGCTCTGGATACCTGGGGCCAAGGGGTCGTCGTCACCGTCTCATCA | CAGGCTGCCCCGACTCAGCCTCCCTCTGTGTCCGGGCCTCCTGGACAGTCGGTCACCATCTCCTGTACTGGAACCAACAGTGATATTGGGTATTTTAATGCTGTGTCTTGGTACCAGCAGCGTCCAGGCGAAGCCCCCAAACTCATGATTCATGAGGTCACTAAGCGGCCCTCAGGGGTCTCTGGTCGCTTCTCTGGCTCCAAGTCTGGCAACACGGCCTCCCTGACCATCTCTGGGCTCCAGAATGAGGATGAGGTTGATTATTACTGCAGCTCATATGCAGGCGACAATACTCTCATATTCGGCGGAGGGACCCGGCTGACCGTCCTG |
| 24C6 | A12N074 | CAGGTGCAGCTACAGGAGTCGGGCCCAGCAGTGGTGAAGCCTTCGGAGACCCTGTCCCTCACCTGCGCTGTCTCTGGTGGCTCCATCAGCAGTAGTAGTTGGTGGAACTGGATCCGCCAGTCCCCAGGGCAGGGGCTGGAATGGATTGGGGGTATCCATGGTAGTGCTGGGAACACCCAATACAACCCCTCCCTCAAGAGTCGAGCCACCATTTCAAGAGACGCGTCCAAGAACCAGTTCTCCCTGAAGGTGAACTATGTGACCGCCGCGGACACCGCCGTGTATTACTGTGCGAGACGCCCCCCCAGTTTTATAACTGGAACTGCGGGCTTTGACTACTGGGGCCAGGGAGTCCTGATCACCGTCTCCTCA | GACATCCAGATGACCCAGTCTCCATCCTCCCTGTCTGCATCTGTGGGAGACAGAGTCACCATCACTTGTCGGGCAAGTCAGGGCATTAGAAGTTATTTAGCCTGGTATCAGCAGAAACCAGGGAAAGCCCCTAACCTCCTGATCTATAAGGCATCCACTTTGCAAAGTGGGGTCCCATCAAGGTTCAGCGGCAGTGGATCTGGGACAGATTTCACTCTCACCATCAGTAGCCTGCAGCCTGAAGATTTTGCAACTTATTACTGTCAACAGCATATTAGTGACCCGTACAGTTTTGGCCAGGGGACCAAAGTGGAGATCAAA |
| 24E17 | A12N074 | CAGGTCACCTTGAAGGAGTCTGGTCCTGCGCTCGTGAAACCCACACAGACCCTCACGCTGACCTGCACCTTCTCTGGGTTCTCACTCACCACTTCTGCAACAGGTGTGGGCTGGATCCGTCAGCCCCCAGGGAAGGCCCTGGAATGGCTTGCCAGCGTTTTTTGGAGTGATAATAAATACTACATCACATCGATGAAGAGCAGGCTCACCATCTCCAAGGACACCTCCAAAAACCAGGTGGTTCTAAGAATGACAAACATGGACCCTATGGACACAGCCACATATTACTGTGCACGGATAATAGCGGGAGCTACGCGATGGTACGGTTTGGATTCCTGGGGCCAAGGGGTCGTCGTCATTGTCTCCTCA | GAAGTTGTAATGACGCAGTCTCCAGCCACCCTGTCTTTGTCTCCAGGGGAAACAGCCACCCTCTCCTGCAGGGCCAGTGAGAGTATTGGCAGCGACTTAGCCTGGTTCCAGCAGAAACCTGGGCAGGCTCCCAAGCTCCTTGTCCGTGGTACATACTTCAGGGCCACTGGCATCCCAGACAGGTTCAGTGGCAGCGGGTCTGGGACAGAGTTCACTCTCACCATTAACAGCCTGGAGCCTGAAGATGTTGGAGTTTATCACTGTCAGCAGTATAATGACTTGCTGTTCACTTTCGGCGGAGGGACCAGGGTGGAGCTCAAA |
| 24D22 | A12N074 | GAAGTGCGACTGGTGCAGTCTGGATCAGAGGTGAAAAGGCCCGGGGAGTCTCTGACGATCTCCTGCCAGACTTCTGGATACACCTTCACCGACAATTGGATCAATTGGGTGCGCCAGCTGCCCGGGAGAGGCCTAGAGTGGATGGGGAGCATCTATCCTGGTGATTCTGATACGAGATACAACCCGTCCTTCCAAGGCCACGTCACTATCTCAGCCGACAAGTCCATCGGCGCCACCTACCTGCAGTGGGACCGCCTGAGGGCCTCGGACACCGCCACATACTATTGTGCTGTCCAGGCCTGTCGTGATGATGGCTGTTCTTCACACTTTGACTGGTGGGGCCAGGGAGTCCTGGTCACCGTCTCCCCA | CAGTCTGTGCTGACGCAGCCGCCCTCAACATCTGGGGCCCCCGGGCAGAGGGTCACCATCTCCTGCACTGGGAGTAGTTCCAACATCGGGGCGAATCATTATGTTTCCTGGTACCAACAGTTCCCAGGAACAGCCCCCAAAGTCCTCATCTTTGAAAATACTGAGCGACCCTCAGGGGTCTCTGGCCGATTCTCTGGCTCCAAGTCTGGTACCTCAGCCTCCCTGACCATCACTGGACTCCAATCTGAGGATGAGGCTGATTATTACTGCTCAGCATGGGATAGCAGCCTCAATTTTCAGATATTCGGCGGAGGGACCCGGCTGACCGTCCTA |

**Table S4. Additional extended multiclade virus panel neutralization for NAb 12C11.**

|  |  | **Titer in TZM.bl cells (ug/ml)** | |
| --- | --- | --- | --- |
|  |  | **12C11** | |
| **Virus ID** | **Clade*** | **IC50** | **IC80** |
| 6535.3 | B | >100 | >100 |
| QH0692.42 | B | >100 | >100 |
| SC422661.8 | B | >100 | >100 |
| PVO.4 | B | >100 | >100 |
| TRO.11 | B | >100 | >100 |
| AC10.0.29 | B | >100 | >100 |
| RHPA4259.7 | B | >100 | >100 |
| THRO4156.18 | B | >100 | >100 |
| REJO4541.67 | B | >100 | >100 |
| TRJO4551.58 | B | >100 | >100 |
| WITO4160.33 | B | >100 | >100 |
| CAAN5342.A2 | B | >100 | >100 |
|  |  |  |  |
| WEAU_d15_410_787 | B (T/F) | >100 | >100 |
| 1006_11_C3_1601 | B (T/F) | >100 | >100 |
| 1054_07_TC4_1499 | B (T/F) | >100 | >100 |
| 1056_10_TA11_1826 | B (T/F) | >100 | >100 |
| 1012_11_TC21_3257 | B (T/F) | >100 | >100 |
| 6240_08_TA5_4622 | B (T/F) | >100 | >100 |
| 6244_13_B5_4576 | B (T/F) | >100 | >100 |
| 62357_14_D3_4589 | B (T/F) | >100 | >100 |
| SC05_8C11_2344 | B (T/F) | >100 | >100 |
|  |  |  |  |
| Du156.12 | C | >100 | >100 |
| Du172.17 | C | >100 | >100 |
| Du422.1 | C | >100 | >100 |
| ZM197M.PB7 | C | >100 | >100 |
| ZM214M.PL15 | C | >100 | >100 |
| ZM233M.PB6 | C | >100 | >100 |
| ZM249M.PL1 | C | >100 | >100 |
| ZM53M.PB12 | C | >100 | >100 |
| ZM109F.PB4 | C | **42.850** | >100 |
| ZM135M.PL10a | C | >100 | >100 |
| CAP45.2.00.G3 | C | >100 | >100 |
| CAP210.2.00.E8 | C | >100 | >100 |
| HIV-001428-2.42 | C | >100 | >100 |
| HIV-0013095-2.11 | C | >100 | >100 |
| HIV-16055-2.3 | C | **56.950** | >100 |
| HIV-16845-2.22 | C | >100 | >100 |
|  |  |  |  |
| Ce1086_B2 | C (T/F) | **65.038** | >100 |
| Ce0393_C3 | C (T/F) | **58.964** | >100 |
| Ce1176_A3 | C (T/F) | >100 | >100 |
| Ce2010_F5 | C (T/F) | >100 | >100 |
| Ce0682_E4 | C (T/F) | >100 | >100 |
| Ce1172_H1 | C (T/F) | >100 | >100 |
| Ce2060_G9 | C (T/F) | >100 | >100 |
| Ce703010054_2A2 | C (T/F) | >100 | >100 |
| BF1266.431a | C (T/F) | >100 | >100 |
| 246F C1G | C (T/F) | >100 | >100 |
| 249M B10 | C (T/F) | **90.726** | >100 |
| ZM247v1(Rev-) | C (T/F) | >100 | >100 |
| 7030102001E5(Rev-) | C (T/F) | >100 | >100 |
| 1394C9G1(Rev-) | C (T/F) | **29.523** | >100 |
| Ce704809221_1B3 | C (T/F) | >100 | >100 |
|  |  |  |  |
| CNE19 | BC | **22.815** | >100 |
| CNE20 | BC | >100 | >100 |
| CNE21 | BC | >100 | >100 |
| CNE17 | BC | >100 | >100 |
| CNE30 | BC | >100 | >100 |
| CNE52 | BC | >100 | >100 |
| CNE53 | BC | >100 | >100 |
| CNE58 | BC | **2.605** | **13.351** |
|  |  |  |  |
| MS208.A1 | A | >100 | >100 |
| Q23.17 | A | >100 | >100 |
| Q461.e2 | A | >100 | >100 |
| Q769.d22 | A | **1.786** | **6.399** |
| Q259.d2.17 | A | >100 | >100 |
| Q842.d12 | A | **52.758** | >100 |
| 0260.v5.c36 | A | >100 | >100 |
| 3415.v1.c1 | A | >100 | >100 |
| 3365.v2.c2 | A | >100 | >100 |
|  |  |  |  |
| 191955_A11 | A (T/F) | >100 | >100 |
| 191084 B7-19 | A (T/F) | **20.210** | **80.854** |
| 9004SS_A3_4 | A (T/F) | >100 | >100 |
|  |  |  |  |
| T257-31 | CRF02_AG | >100 | >100 |
| 928-28 | CRF02_AG | >100 | >100 |
| 263-8 | CRF02_AG | >100 | >100 |
| T250-4 | CRF02_AG | >100 | >100 |
| T251-18 | CRF02_AG | >100 | >100 |
| T278-50 | CRF02_AG | >100 | >100 |
| T255-34 | CRF02_AG | >100 | >100 |
| 211-9 | CRF02_AG | >100 | >100 |
| 235-47 | CRF02_AG | >100 | >100 |
|  |  |  |  |
| 620345.c01 | CRF01_AE | >100 | >100 |
| CNE8 | CRF01_AE | >100 | >100 |
| C1080.c03 | CRF01_AE | >100 | >100 |
| R2184.c04 | CRF01_AE | >100 | >100 |
| R1166.c01 | CRF01_AE | >100 | >100 |
| R3265.c06 | CRF01_AE | >100 | >100 |
| C2101.c01 | CRF01_AE | >100 | >100 |
| C3347.c11 | CRF01_AE | >100 | >100 |
| C4118.c09 | CRF01_AE | >100 | >100 |
| CNE5 | CRF01_AE | >100 | >100 |
| BJOX009000.02.4 | CRF01_AE | >100 | >100 |
|  |  |  |  |
| BJOX015000.11.5 | CRF01_AE (T/F) | >100 | >100 |
| BJOX010000.06.2 | CRF01_AE (T/F) | >100 | >100 |
| BJOX025000.01.1 | CRF01_AE (T/F) | >100 | >100 |
| BJOX028000.10.3 | CRF01_AE (T/F) | >100 | >100 |
|  |  |  |  |
| X1193_c1 | G | >100 | >100 |
| P0402_c2_11 | G | **29.342** | **87.411** |
| X1254_c3 | G | >100 | >100 |
| X2088_c9 | G | >100 | >100 |
| X2131_C1_B5 | G | >100 | >100 |
| P1981_C5_3 | G | >100 | >100 |
| X1632_S2_B10 | G | **27.006** | >100 |
|  |  |  |  |
| 3016.v5.c45 | D | >100 | >100 |
| A07412M1.vrc12 | D | >100 | >100 |
| 231965.c01 | D | >100 | >100 |
| 231966.c02 | D | >100 | >100 |
| 6405.v4.c34 | D | >100 | >100 |
|  |  |  |  |
| 3817.v2.c59 | CD | >100 | >100 |
| 6480.v4.c25 | CD | **9.025** | **53.006** |
| 6952.v1.c20 | CD | >100 | >100 |
| 6811.v7.c18 | CD | **32.440** | >100 |
| 89-F1_2_25 | CD | >100 | >100 |
|  |  |  |  |
| 3301.v1.c24 | AC | >100 | >100 |
| 6041.v3.c23 | AC | >100 | >100 |
| 6540.v4.c1 | AC | >100 | >100 |
| 6545.v4.c1 | AC | >100 | >100 |
|  |  |  |  |
| 0815.v3.c3 | ACD | >100 | >100 |
| 3103.v3.c10 | ACD | >100 | >100 |
|  |  |  |  |
| MuLV | Neg. Control | >100 | >100 |

**Table S5. Cryo-EM data collection, refinement and validation statistics.**

|  | GT1.1 + 12C11 + RM20A3  (EMD-40796)  (PDB 8SW3) | GT1.1 + 21N13 + 21M20 + RM20A3  (EMD-40797) (PDB 8SW4) |
| --- | --- | --- |
| **Data collection and processing** |  |  |
| Microscope | TFS Glacios | TFS Glacios |
| Voltage (keV) | 200 | 200 |
| Camera  Collection mode  Magnification  Pixel size at detector (Å) | TFS Falcon 4  Counting  190,000x  0.725 | TFS Falcon 4  Counting  190,000x  0.725 |
| Total electron exposure (e–/Å^2^) | 40 | 40 |
| Exposure rate (e-/pixel/sec)  Number of EER frames  Defocus range (μm) | 5.30  40  -0.5 to -1.8 | 5.30  40  -0.5 to -1.8 |
| Automation software | EPU | EPU |
| Micrographs collected (no.) | 5,627 | 6,506 |
| Micrographs used (no.) | 5,608 | 6,260 |
| Initial particle images (no.) | 1,115,637 | 1,034,294 |
| Final particle images (no.) | 456,166 | 242,098 |
| Symmetry  Map resolution (masked/unmasked Å)  FSC threshold | C3  2.8/3.2  0.143 | C1  2.9/3.4  0.143 |
| Map sharpening *B* factor (Å^2^) | -104.1 | -70.7 |
| Map pixel size (Å) | 1.009 | 1.044 |
| Map resolution range (Å) | 2.4-4.6 | 2.4-4.1 |
|  |  |  |
| **Refinement** |  |  |
| Initial model used (PDB code) | 6X9R | 6X9R |
| Refinement package | Phenix real space refine | Phenix real space refine |
| Model resolution (Å)  FSC threshold | 2.9  0.5 | 3.0  0.5 |
| EMRinger score | 4.35 | 4.31 |
| CC (mask) | 0.75 | 0.82 |
| Model composition  Non-hydrogen atoms  Protein residues  Ligands | 24,819  3,099  60 | 26,323  3,303  47 |
| Mean *B* factors (Å^2^)  Protein  Ligand | 64.02  71.08 | 57.07  62.86 |
| R.m.s. deviations  Bond lengths (Å)  Bond angles (°) | 0.007  1.356 | 0.007  1.165 |
| Validation  MolProbity score  Clashscore  Poor rotamers (%) | 1.00  1.84  0.19 | 1.08  2.93  0.14 |
| Ramachandran plot  Favored (%)  Allowed (%)  Disallowed (%) | 97.73  2.27  0.00 | 98.08  1.92  0.00 |
| Cβ outliers (%) | 0.00 | 0.00 |
| CaBLAM outliers (%) | 1.71 | 1.45 |
|  |  |  |
|  |  |  |

**Table S6. X-ray data collection and reﬁnement statistics**

|  | **21N13 Fab** | **21N13 Fab +BG505 + 35O22 scFv** |
| --- | --- | --- |
| **Data Collection** | | |
| Beamline | APS-23ID-D | SSRL12-1 |
| **Resolution (Å)** | 47.68-2.50 (2.54-2.50) | 43.63-4.70 (4.78-4.70) |
| Wavelength, Å | 1.0332 | 0.9795 |
| Space group | C 1 2 1 | P2_1_3 |
| Unit cell a, b, c (Å) | 260.5 45.2 84.6 | 265.4 265.4 265.4 |
| α, β, γ (°) | 90 98.5 90 | 90 90 90 |
| Copies per ASU | 2 | 1 |
| Completeness | 99.9 (99.9) | 100.0 (100.0) |
| Redundancy | 6.1 (5.8) | 40.3 (35.3) |
| No. total reflections | 213,542 | 1,319,858 |
| No. unique reflections | 34,904 (1698) | 32,727(1623) |
| I/σ | 8.9 (3.2) | 14.2 (1.0) |
| Rsym | 0.34 (1.7) | 0.35 (4.2) |
| Rpim | 0.15 (0.75) | 0.06 (0.71) |
| CC1/2 | 0.83 (0.35) | 0.99 (0.30) |
| **Refinement Statistics** | | |
| Resolution (Å) | 47.68-2.46 (2.53-2.50) | 43.63-4.69 (4.83-4.70) |
| No. reflections total/Rfree | 33,176 / 1711 | 31,038 /1733 |
| Rcryst | 18.2 (23.8) | 28.4 (36.9) |
| Rfree | 22.0 (29.6) | 30.5 (37.1) |
| RMSD bond length (Å) | 0.007 | 0.04 |
| RMSD bond angles (°) | 0.96 | 0.61 |
| Number of atoms | | |
| All proteins | 6544 | 11435 |
| 21N13 Fab | 6544 | 3358 |
| 35O22 scFv | / | 1839 |
| gp120 | / | 3587 |
| gp41 | / | 1166 |
| Water | 383 | / |
| Glycan | / | 705 |
| Average B-value (Å^2^) | | |
| All proteins | 27 | 302 |
| 21N13 Fab | 27 | 287 |
| 35O22 scFv | / | 322 |
| gp120 | / | 303 |
| gp41 | / | 306 |
| Water | 29 | / |
| Glycan | / | 373 |
| Wilson B-value (Å^2^) | 25 | 249 |
| Ramachandran Favored% | 98.1 | 95.1 |
| Ramachandran Outliers% | 0 | 0 |
| PDB | 8D01 | 8D0Y |
